# The Role and Mechanism of SIRT1 in Resveratrol-regulated Osteoblast Autophagy in Osteoporosis Rats

**DOI:** 10.1038/s41598-019-44766-3

**Published:** 2019-12-05

**Authors:** Xuhao Yang, Tianlong Jiang, Yu Wang, Lei Guo

**Affiliations:** Department of Orthopedic Surgery, First Affiliated Hospital, China Medical University, Shenyang, Liaoning 110001 P.R. China

**Keywords:** Molecular medicine, Osteoporosis, Molecular medicine, Osteoporosis, Molecular medicine

## Abstract

Osteoporosis is widely regarded as one of the typical aging-related diseases due to the impairment of bone remodeling. The silent information regulator of transcription1 (SIRT1) is a vital regulator of cell survival and life-span. SIRT1 has been shown to be activated by resveratrol treatment, and also has been proved to prevent aging-related diseases such as osteoporosis. However, the role of SIRT1 about autophagy or mitophagy of osteoblasts in resveratrol-regulated osteoporotic rats remains unclear. This study seeks to investigate the role of SIRT1 about autophagy or mitophagy in osteoblasts through PI3K/Akt signaling pathway in resveratrol-regulated osteoporotic rats. The vivo experiment results have revealed that resveratrol treatment significantly improved bone quality and reduced the levels of serum alkaline phosphatase and osteocalcin in osteoporotic rats. Moreover, Western bolt analysis showed that expression of SIRT1, LC3, and Beclin-1 in osteoblasts increased, while *p*-AKT and *p*-mTOR were downregulated in osteoporosis rats with high dose resveratrol treatment. On the other hand, resveratrol treatment increased the SIRT1 activity, LC3 and Beclin-1 mRNA expression in the dexamethasone (DEX)-treated osteoblasts. More mitophagosomes were observed in the DEX-treated osteoblasts with resveratrol. Meanwhile, the TOM20, Hsp60, *p*-Akt and *p*-mTOR activities were decreased in the DEX-treated osteoblasts with resveratrol. Resveratrol treatment did not change the *p*-p38 and *p*-JNK activities in the osteoblasts. These results revealed that resveratrol treatment protected osteoblasts in osteoporosis rats by enhancing mitophagy by mediating SIRT1 and PI3K/AKT/mTOR signaling pathway.

## Introduction

Nowadays osteoporosis has become a primary public health concern in many countries, because of both the risk of potentially serious fractures and its prevalence is increasing as the population ages^1^. Osteoporosis is seen as a typical aging-related disease caused by the impairment of bone remodeling, which accompanied with an imbalance in the number and activity of osteoblasts and osteoclasts^2–4^. Osteoblasts are functional cells of osteogenesis and responsible for bone formation^5,6^. This may explain that hypoactivation of osteoblasts leads to aging-related osteoporosis. The increase in the number and function of osteoblasts can promote bone formation, which seems to be a primary strategy for osteoporosis prevention. Furthermore, a growing number of studies have observed that osteoblast dysfunction is seen as the primary reason for bone loss^7^. Currently the drugs of prevention and treatment of osteoporosis are primarily focused on promoting the proliferation and differentiation of osteoblasts.

Autophagy is the major catabolic process by which eukaryotic cells degrade and recycle the impaired macromolecules or organelles. The degraded material is isolated in a double-membrane vesicle termed autophagosome, and then delivers to the lysosomes^8,9^. After fusion with lysosomes, the autophagosomes and their contents are cleared up, and then these products can recycle for the bioenergetics metabolites^10–12^. Under normal condition, autophagy usually occurs at basal level in most cells, but it can be activated under stress in an attempt to survival^13–15^. Recently a strong body of studies claims that autophagy, as a cell survival pathway, plays a vital role in maintaining bone homeostasis and changes in this pathway are to some extent associated with osteoporosis^16–21^. In addition, despite the deficiency of age-related estrogen has been widely recognized as a primary cause of osteoporosis^22^, now the increased oxidative stress in bone tissue associated with aging is considered to be one of the major contributing factors in osteoporosis^23–25^.

The silent information regulator of transcription1 (SIRT1) is one of the sirtuin family of NAD+-dependent Class III histone deacetylases, which affects the deacetylation of protein substrates in the enzymatic cleavage of NAD. It is noticeable that SIRT1 takes an important part in the regulation of many biological functions including tumor inhibition, mitochondrial homeostasis, energy metabolism, cellular ageing and cell death. SIRT1 overexpression or deletion increases or decreases bone mass, respectively^26–28^. Activation of SIRT1 in mice is correlated with delayed onset of multiple related diseases such as osteoporosis^29–32^. A body of studies has confirmed that SIRT1 is activated by oxidative stress and resveratrol treatment^33,34^. Resveratrol (RES) is one of the main focuses of studied polyphenolic compounds which are enriched in red wines, grapes and other various food sources. Several scholars observe that the women preferring to drink red wine may have a low risk of hip fracture^35^. Recently, resveratrol has attracted much attention because of its beneficial roles in antioxidant, and antitumor effects and longevity. Moreover, resveratrol is reported to act as an activator of SIRT1 both *in vitro* and *in vivo*. Although it is still not completely clear about the ways in which resveratrol performs its biological function, its activation of SIRT1 has been considered as an important mechanism for improving the cellular function and organismal health. It is noticeable that resveratrol through activating SIRT1 have played a crucial role in the balance between the osteoblastic and osteoclastic activity, which affects bone formation *in vitro*^36,37^. In addition, resveratrol is proved to maintain bone mineral density in elderly mice *in vivo*^38^. The findings of the existing studies indicate that resveratrol inhibits the hydrogen dioxide-induced apoptosis by activation of SIRT1 in osteoblasts^39^. However, the mechanism through which resveratrol activates SIRT1 acts on osteoblasts remains to be elucidated. It is noticeable that SIRT1 activators, such as resveratrol, stimulate bone formation and prevent the loss of bone mass, which may provide a new strategy for the therapy for osteoporosis.

This study seeks to provide in-depth understanding of the mechanism behind the autophagy effect of SIRT1 in the resveratrol-treated osteoblasts in osteoporotic rats. We herein demonstrate that resveratrol mediates PI3K/AKT/mTOR signaling pathway by SIRT1 activation and enhances mitophagy in osteoblasts in osteoporotic rats.

## Materials and Methods

### Animals and osteoporosis model

All animal procedures were carried out in line with the protocols approved by the Institutional Animal Care and Use Committee of China Medical University. The male Sprague-Dawley (SD) rats (3-month old, weighing 280–340 g) were purchased from Beijing Vital River Laboratory Animal Technology Co., Ltd.). Animals were housed in the specific pathogen-free conditions (temperature at 20 ± 1 °C, 12-hour light/dark cycles and 50 ± 5% humidity) with food and water. The rats were randomized into the following groups: control group (n = 5), osteoporosis group (n = 5) and osteoporosis + resveratrol group (n = 10). Osteoporosis model groups performed by injected intramuscular injection of dexamethasone 5 mg/kg twice a week for 6 weeks to induce osteoporosis model while the control group was injected with normal saline. Osteoporosis + resveratrol groups were randomly divided into two experimental groups: low-dose group and high-dose group. Seven days after successful modeling, resveratrol (Sigma, USA) was given orally at 5 mg/kg/d and 45 mg/kg/d (low-dose group and high-dose group) for 8 weeks, respectively.

### Bone mineral density measurement

Bone mineral density (BMD) was measured using dual-energy X-ray absorption assay (DEXA) and relevant evaluation software. Briefly, the BMD value was measured for the right femur of each rat, and put them in the position tray and scanned. All the samples were placed in a similar direction and the proximal epiphysis of the femur was used for X-ray analysis.

### ELISA assay

Blood samples (2 ml) were collected from the right common carotid artery of rats in each group and centrifuged at a speed of 3000 × g at 4 °C for 20 minutes. The serum of ALP and OC were measured by colorimetric analysis at 450 nm, according to the manufacturer’s instruction (Elabscience Biotechnology Co., Wuhan, China).

### Immunohistochemistry staining (IHC)

The bone samples were obtained from the right femur of the rats. Subsequently, the samples were fixed in paraformaldehyde (4%) for 24 h and decalcified in EDTA solution (10%) for 2 weeks. After decalcification, the samples were embedded with paraffin and cut into slices. The slices were dewaxed in xylene for 30 min, and placed in enthanol (100%) for 5 min, enthanol (90%) for 2 min, enthanol (80%) for 1 min. Then the slices were washed with distilled water and PBS buffer. The goat serum (10%) was used to block the slices for 30 min, and first antibody was incubated overnight. The next day the slices were washed with PBS and incubated with secondary antibody for 1 h. After washed with PBS, DAB was added for 2 min and terminated with water. The slices were subjected to enthanol (80%) for 3 min, enthanol (90%) for 3 min, enthanol (100%) for 5 min, xylene for 20 min, then were sealed with resin.

### Chemicals and reagents

Cell culture media, minimum essential medium-alpha (α-MEM), and fetal bovine serum (FBS) were purchased from Gibco (Grand Island, USA). Resveratrol was obtained from Aldrich (Sigma, USA). The PI3K inhibitor (LY294002), mTOR inhibitor (S1842), p38 inhibitor (SB203580), JNK inhibitor (SP600125), and 4,6-diamidino-2-phenylindole (DAPI) were purchased from Beyotime Biotechnology (Jiangsu, China). The SIRT1 inhibitor NAM was purchased from Sigma (USA). The polyclonal rabbit anti-mouse primary translocase of outer membrane (TOM)20 antibody (sc-11415) (1: 200 for Western blot), the monoclonal mouse anti-mouse microtubule-associated protein 1A/1B-light chain 3 (LC3) antibody (sc-376404) (1: 100 for Western blot), and the polyclonal, goat anti-mouse heat shock protein 60 (Hsp60) antibody (sc-1052) (1: 200 for Western blot) were all obtained from Santa Cruz Biotechnology (California, USA). The total/phosphor-AKT polyclonal antibody and total/phosphor-p38 antibody were purchased from Abcam (Cambridge, USA). The total/phosphor-mTOR antibody was purchased from Sigma-Aldrich (Sigma, USA). The total/phosphor-JNK antibody was purchased from ImmunoWay (Newark, USA). Trizol reagent was purchased Life Technologies Co. (Carlsbad, USA). TaqMan reagents were purchased from Takara (Otsu, Japan). The immunofluorescence staining kit was purchased from Beyotime Biotechnology (Jiangsu, China).

### Cell culture of MC3T3-E1 murine osteoblasts

MC3T3-E1 cells were purchased from the XIEHE Cell Repository in Beijing, China. Cells were subcultured in α-MEM with 10% FBS and grown under the humidified atmosphere containing 5% CO_2_ at 37 °C. The cell culture medium was replaced every two days. Cells were subjected to different concentrations of resveratrol (0 M, 10^−8^ M, 10^−7^ M and 10^−6^ M) for 24 hours. SIRT1 inhibitor (NAM), PI3K inhibitor (LY294002), mTOR inhibitor (S1842), p38 inhibitor (SB203580), and JNK inhibitor (SP600125) were also used to treat the osteoblast cells after exposure or non-exposure to 10^−6^ M dexamethasone.

### Protein preparation and western blot analysis

In the vivo experiment, the bone samples were obtained from the left femur of the rats. Then the samples were homogenized and lysed in radio-immunoprecipitation assay (RIPA) buffer containing protease inhibitors which including phosphatase inhibitors and phenylmethylsulfonyl fluoride. *In vitro* part, the cells were washed with PBS and then all the protein was harvested in RIPA buffer. We collected the cell and their lysates and centrifuged them at 12000 g for ten minutes at 4 °C. Then we collected the supernatants and mixed them with 5× loading buffer, and denatured them by boiling for ten minutes. We separated the samples by sodium dodecyl sulfate-polyacrylamide gel electrophoresis (SDS-PAGE). Then the samples were transferred to polyvinylidene fluoride (PVDF) membranes using a transfer buffer at 70 V for 1.5 hours. We incubated the membranes with Tris-buffered saline (TBS) containing Tween 20 (TBST) and 5% bovine serum albumin for 120 minutes. Then we washed them 3 times with TBST for 30 minutes. We incubated the membranes with the related primary antibody overnight at 4 °C, and then followed by horseradish peroxidase (HRP)-labeled secondary antibody for 1.5 hours. After washed the membranes three times with TBST for 30 minutes, we used the BeyoECL plus kit (Beyotime, China) to visualize the proteins.

### Real-time polymerase chain reaction (RT-PCR)

The RT-PCR method was adopted to extract RNA with trizol, reverse-transcribed mRNA to cDNA, amplified cDNA with PCR amplifications. Total RNA was extracted from MC3T3-E1 cells using trizol reagent. Then the expression of SIRT1, LC3 and Beclin-1 mRNA were detected by real-time PCR using TaqMan reagents. The specific primers were used as followed:

SIRT1 forward: 5′-GTTGTGTGCCTTCGTTTTGGA-3′

SIRT1 reverse: 5′-AGGCCGGTTTGGCTTATACA-3′

LC3 forward: 5′-CTCTCTGAGCCTTAGGTGCC-3′

LC3 reverse: 5′-ACTCGTGGGGTGACCATTTC-3′

Beclin-1 forward: 5′-GAATGGAGGGGTCTAAGGCG-3′

Beclin-1 reverse: 5′-CCTCTTCCTCCTGGCTCTCT-3′

GAPDH forward: 5′-AGTCTACTGGCGTCTTCACC-3′

GAPDH reverse: 5′-CCACGATGCCAAAGTTGTCA-3′

The PCR reactions were performed with the following conditions: incubated at 95 °C for 30 s, degenerated at 95 °C for 5 s, annealed at 55 °C for 10 s, and extended at 72 °C for 15 s, then cycled 40 times, and finally extended at 72 °C for 6 min. After amplification, 5 µl of PCR product and 1 µl 6× DNA loading buffer was added and then used for electrophoresis at 60 V. We used Primer Premier 5.0 software (Premier Biosoft International, USA) to design all the primers.

### Confocal immunofluorescence microscopy

The cells were cultured in six-well plates. After incubation for 24 hours, the cells were fixed with 4% paraformaldehyde for 30 minutes at 4 °C. The cells were blocked at nonspecific antibody sites by 5% BSA in TBST for 30 minutes after washing with PBS three times. The cells were then incubated with specific primary rabbit anti-SIRT1 antibodies (1: 1000) or primary antibody anti-LC3B (1:200) overnight at 4 °C. Then, the cells were washed with PBS again, and followed by the secondary antibody using a goat anti-rabbit IgG (1: 3000) or the secondary antibody anti-DyLight 594 (1:200) for 1 h. Subsequently, these cells were stained with DAPI for 5 minutes, and followed by washed with PBS for 15 minutes. The immunofluorescence-stained cells were observed with an Olympus FV1000 confocal laser-scanning microscope with a peak emission wavelength of 518 nm (green) and 565 nm (red).

### Transmission electron microscopy (TEM)

The cells were harvested and fixed in 2.5% glutaraldehyde PBS for 2 hours at indoor temperature. After being post-fixed in 1% osmium tetroxide in water for 1 hour, the cells were then stained in 2% uranyl acetate in water for 1 hour in the dark. After being subjected to gradient ethanol dehydration, the cells were embedded and sectioned. Subsequently, the samples were double-stained with uranyl acetate and lead citrate. The samples were viewed with using a JEM-1200EX transmission electron microscope (TEM) (Tokyo, Japan).

### Cell proliferation assay

The cells were seeded in 96-well plates. After cultured for 24 hours, the cells were treated with or without 10^−6^ M dexamethasone, with or without resveratrol, with or without NAM. Then we added 10 mM BrdU solution into the culture medium, and further incubated for 2.5 hours. After the cells were fixed in 4% paraformaldehyde, we used the Cell Proliferation ELISA BrdU colorimetric kit (Roche Diagnostics) to measure the BrdU incorporation.

### Cell viability assay

In brief, after various treatments to the cells, 10 µl of MTT reagent (5 mg/ml) were added to the cells, and the cells were incubated at 37 °C for 4 hours. Then, we removed the culture medium and dissolved the formazan crystals with 100 µl of dimethyl sulfoxide. The optical density (OD) was measured by using a Spectra Max Plus 384 Microplate Reader (Molecular Devices, Germany) at 570 nm wavelength.

### Statistical analysis

All data were represented as the mean ± standard deviations (SD). The homogeneity test of variance test was performed by Leven test. Differences between specific groups were determined by one-way analysis of variance (ANOVA) and the least significant difference (LSD) test. P < 0.05 was considered to represent a statistically significant difference. Statistical analysis was conducted with the assistance of SPSS 15.0 software for Windows (SPSS, Inc., Chicago, IL, USA).

## Results

### Resveratrol treatment improved bone quality in osteoporotic rats

**Figure 1:**
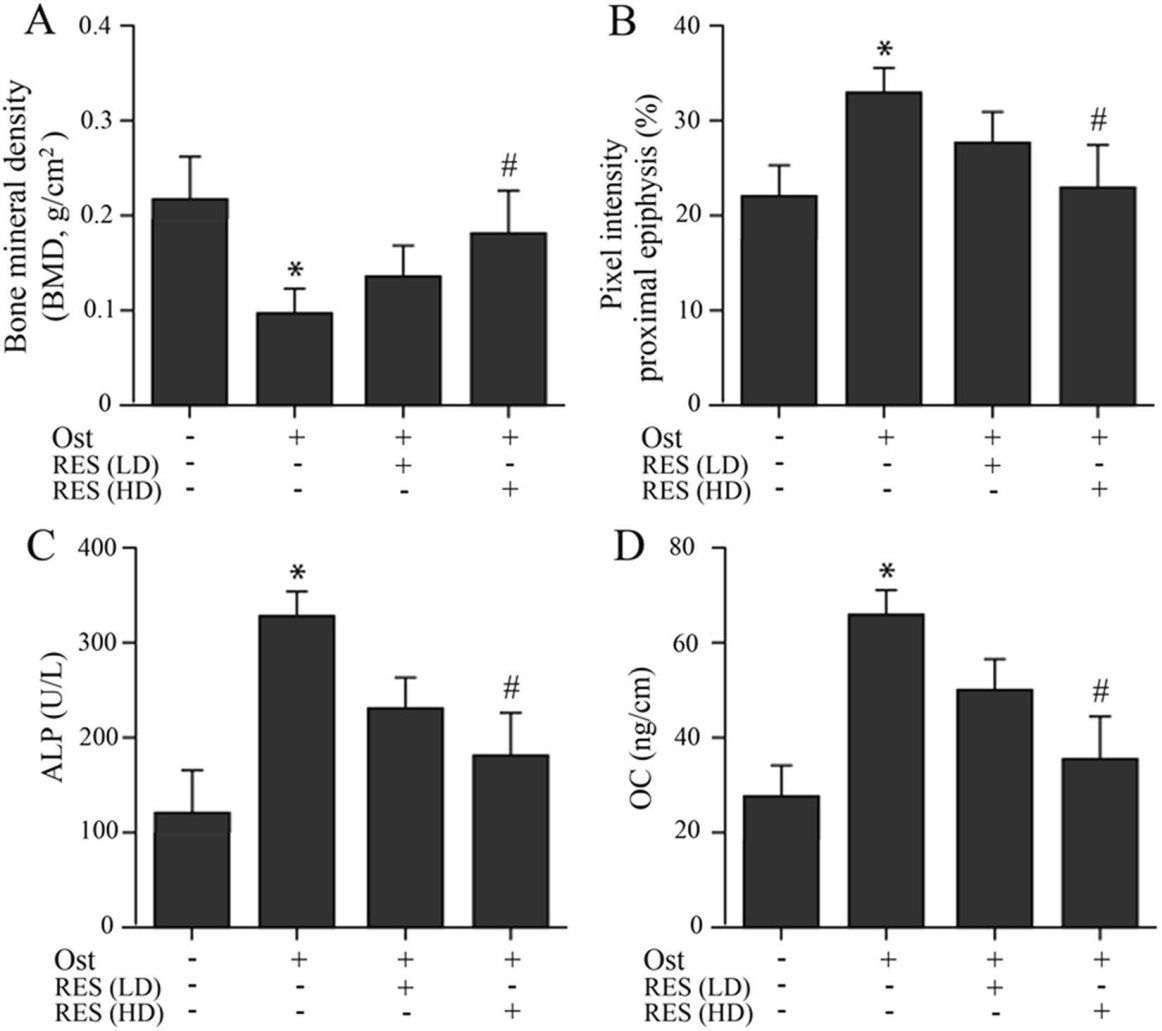
Bone mineral density (BMD) and femoral porosity based on the pixel intensity of proximal epiphysis and serum bio-marker [alkaline phosphatase (ALP) and osteocalcin (OC)] changes in osteoporosis and resveratrol treated groups. (**A)** Resveratrol treatment restored BMD reduction in osteoporosis rats. **(B)** Femoral porosity based on the pixel intensity of proximal epiphysis changes in control, osteoporosis and resveratrol treated groups. **(C)** ALP changes in control, osteoporosis and resveratrol treated groups. **(D)** OC changes in control, osteoporosis and resveratrol treated groups. LD, low-dose; HD, high-dose. *P < 0.05 vs. control group; ^#^*P* < 0.05 vs. Ost group.

In the osteoporosis group, the bone mineral density (BMD) value was evidently decreased when compared to the control group (Fig. 1A). BMD was elevated in resveratrol treatment groups compared to osteoporosis group, whether in low-dose or high-dose. And the BMD of the osteoporosis rats was significantly increased in the treatment of high-dose resveratrol. Furthermore, as the Fig. 1B shown, the treatment with high-dose resveratrol group remarkably reduced the femoral porosity based on the pixel intensity of proximal epiphysis.

From the blood sample analysis results, the levels of serum ALP and OC in the osteoporosis group were obviously higher when compared with those of the control group (Fig. 1C,D). However, whether with low- or high-dose resveratrol treatment, both ALP and OC were decreased in the osteoporosis rats, and the treatment with high-dose resveratrol obviously reduced the serum levels of ALP and OC in the osteoporosis rats.

**Figure 2:**
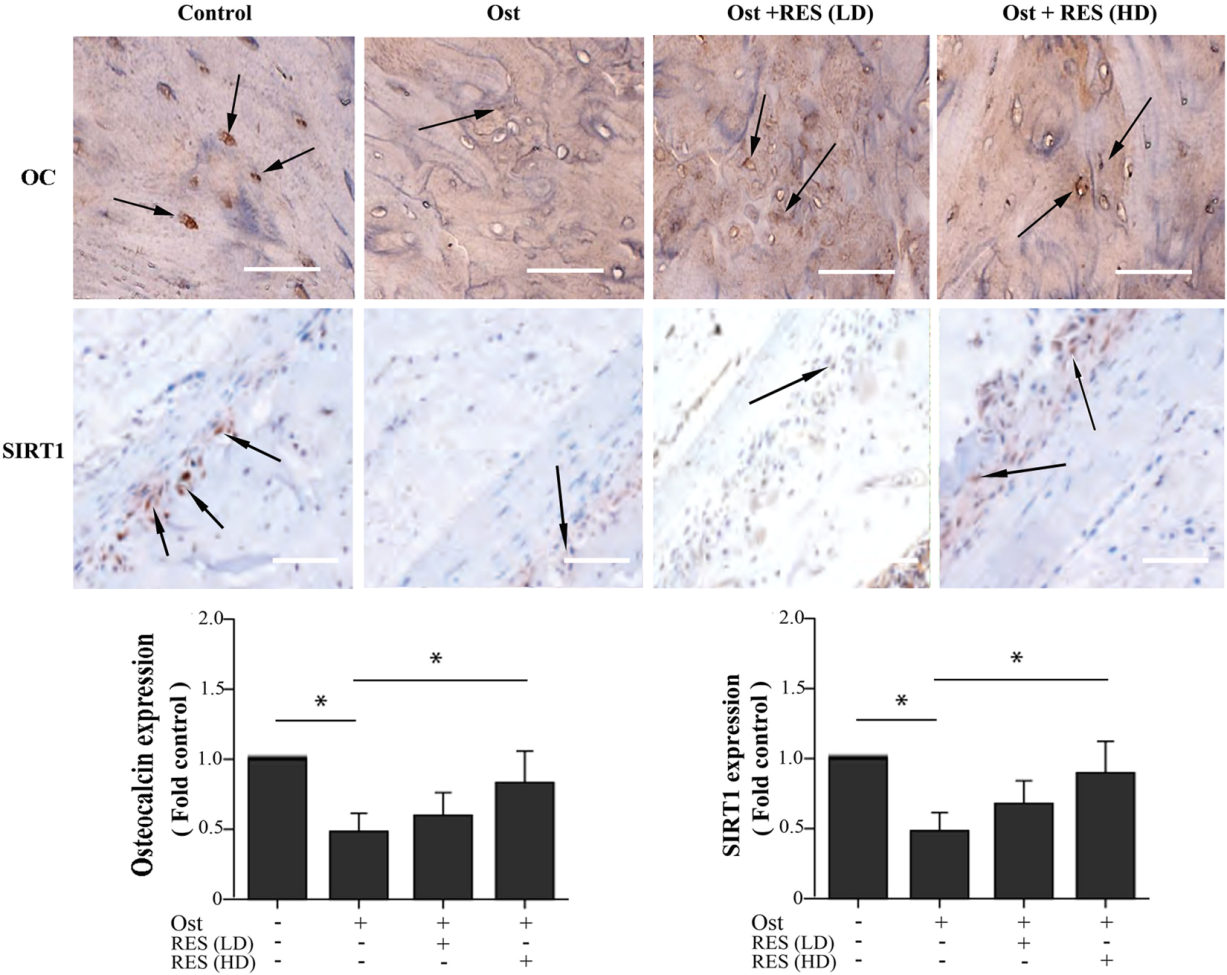
The expression of osteocalcin and SIRT1 in the rats’ femur of different groups by using immunohistochemical staining. Ost, osteoporosis; RES, resveratrol; LD, low-dose; HD, high-dose.

As the IHC results shown (Fig. 2), the expression of osteocalcin and SIRT1 in the osteoporosis group was decreased significantly, while the high-dose resveratrol group significantly increased their expressions when compared with osteoporosis group. Moreover, the structure of the trabecular bone in the osteoporosis group was sparse, and the trabecular structure was improved after receiving high-dose resveratrol treatment. These results indicated that resveratrol treatment improved bone quality in osteoporotic rats.

### Resveratrol enhanced SIRT1 expression and induced autophagy via Akt/mTOR pathway in bone cells of osteoporosis rats

**Figure 3:**
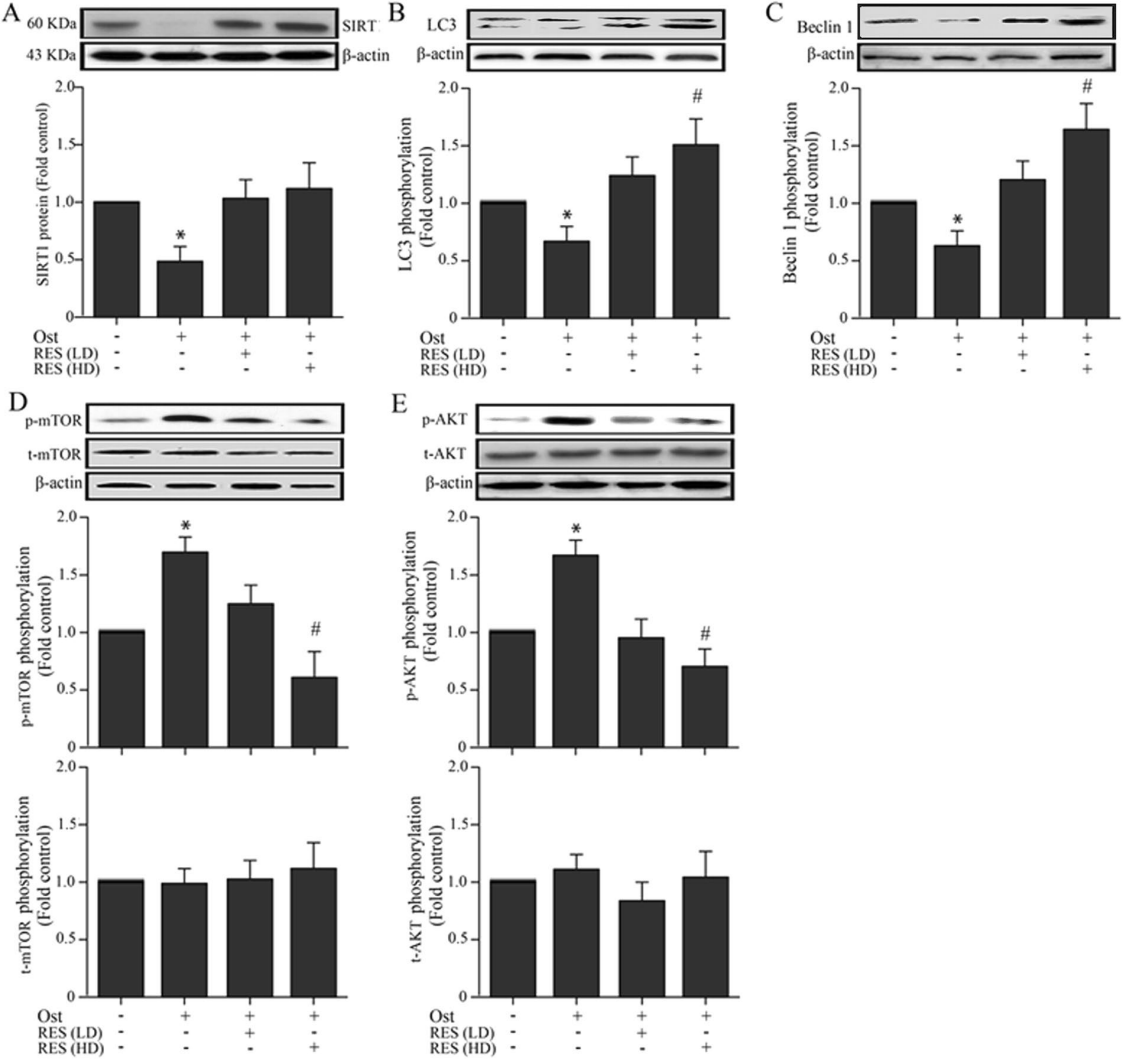
Western blot showing expression of the SIRT1, LC3, Beclin-1, *p*-mTOR and *p*-AKT in osteoblasts among the groups. **(A)** Western blot shows the expression of SIRT1 expression in osteoblasts of different groups. **(B)** Western blot shows the expression of LC-3 in osteoblasts of different groups. **(C)** Western blot shows the expression of Beclin-1 in osteoblasts of different groups. **(D)** Western blot shows the expression of *p*-mTOR in osteoblasts of different groups. (**E**) Western blot shows the expression of *p*-AKT in osteoblasts of different groups. *P < 0.05 vs. control group; ^#^*P* < 0.05 vs. Ost group. Ost, osteoporosis; RES, resveratrol; LD, low-dose; HD, high-dose.

Western blot assay was performed to measure expression of SIRT1 protein in bone cells of osteoporotic rats. As shown by Fig. 3A, and the supplementary file, the osteoporosis group significantly reduced the expression of SIRT1 in cells compared to the normal control group. Moreover, treatment with resveratrol with low-dose or high-dose both increased the protein expression of SIRT1 in the osteoporosis rats.

Revealed by the western blot results (Fig. 3B,C), there was a significant decreased expression of LC3 and Beclin-1 in osteoporosis group when compared with the normal control group. While in osteoporosis with high dose resveratrol treatment, the expression of LC3 and Beclin-1 protein were significantly increased, indicating that resveratrol may induce autophagy of bone cells. We have placed a focus on the Akt/mTOR signaling pathway as it played important role in regulating autophagy in order to unpack which pathway mediated resveratrol-induced cell autophagy activation in osteoporotic rats. We tested the expression level of phospho-mTOR (*p*-mTOR), total mTOR (*t-*mTOR), phospho-Akt (*p*-Akt) and total Akt (*t*-Akt) in osteoblasts of all the groups. The western blot results illustrated that Akt phosphorylation and mTOR phosphorylation was down-regulated in the osteoporotic rats with high-dose resveratrol treatment group when compared with the osteoporotic rats group. However, the expression of *t*-Akt and *t*-mTOR had no significantly changes among the groups (Fig. 3D,E). It is found that the Akt/mTOR pathway may mediate resveratrol-induced bone cells autophagy activation in osteoporotic rats.

### Resveratrol enhanced SIRT1 expression and protect DEX-treated osteoblasts by autophagy

**Figure 4:**
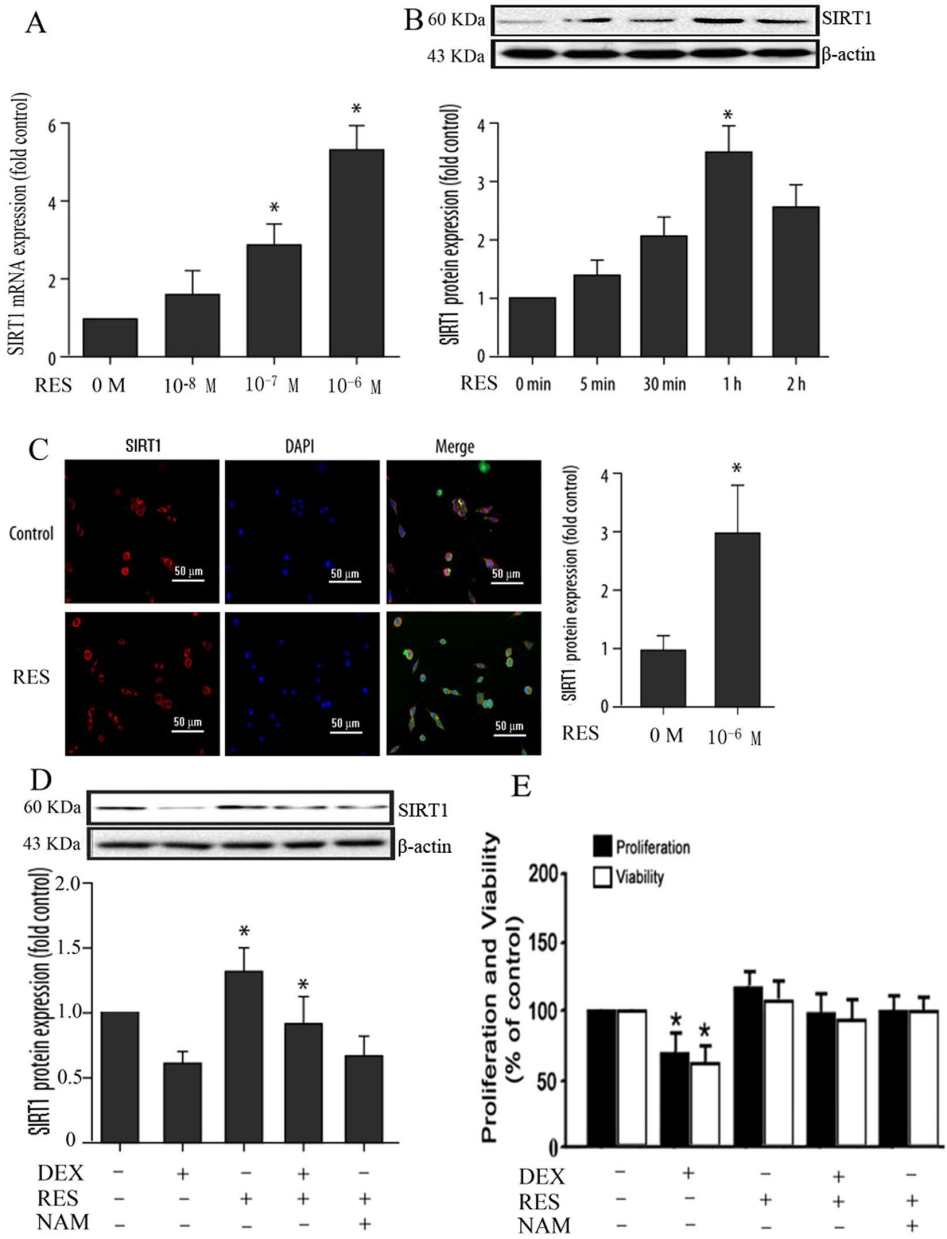
Expression of the SIRT1 in osteoblasts of different groups. **(A)** Expression of SIRT1 mRNA in osteoblasts treated with increasing concentrations of resveratrol (0 M, 10^−8^ M, 10^−7^ M, 10^−6^ M), as assessed with quantitative real-time polymerase chain reaction (RT-PCR). **(B)** Expression of SIRT1 in osteoblasts treated with resveratrol (50 µM) for 0, 5, 30, 60, 120 min, as assessed with Western blot. **(C)** Confocal immunofluorescence (IF) staining of osteoblasts with or without resveratrol stained for SIRT1 (red). Nuclei were stained with 4′,6-diamidino-2-phenylindole (DAPI) (blue). The intensity of SIRT1 was higher in serum-starved osteoblasts cells cultured in serum-free medium with resveratrol. **(D)** Expression of SIRT1 protein in osteoblasts among different groups, as assessed with Western blot. **(E)** BrdU and MTT assays for proliferation and viability of osteoblasts that were treated with or without dexamethasone, with or without resveratrol and NAM. Analysis of the proliferation and viability of osteoblasts with different treatments as assessed with the BrdU assays and the MTT assay. The figures represent three independent experiments. Asterisks indicate significant differences compared to the control group (*P < 0.05).

To investigate the effect of resveratrol on the expression of SIRT1 in osteoblasts, we examined the SIRT1 mRNA expression in osteoblasts treated with various concentrations of resveratrol (10^−8^ to 10^−6^ M) and SIRT1 protein expression in osteoblasts when serum-starved osteoblasts were treated with resveratrol (50 µM) for 5, 30, 60, 120 mins. It is found that resveratrol enhanced SIRT1 expression in osteoblasts in a dose-dependent and time-dependent way (Fig. 4A,B). Then we further utilized immunofluorescence to detect the expression of SIRT1 in osteoblasts with or without resveratrol treatment, and the result indicated that the SIRT1 expression in osteoblasts with resveratrol treatment was significantly higher than the without resveratrol group (Fig. 4C).

It has been reported that excess glucocorticoids do harm to osteoblasts. However, whether resveratrol can improve the proliferation and viability of DEX-treated osteoblasts by modulating autophagy is unclear. We exposed osteoblasts treated with DEX, with or without RES, or with NAM treatment. The western blot results showed that the dexamethasone reduced the SIRT1 expression in osteoblasts and the treatment of resveratrol obviously increased the SIRT1 expression in osteoblasts, while the NAM abolished the effect of resveratrol on SIRT1 expression in osteoblasts (Fig. 4D). The BrdU and MTT assay result indicated that with resveratrol treatment significantly improved the proliferation and viability of osteoblasts when compared to the DEX-treated group (Fig. 4E).

**Figure 5:**
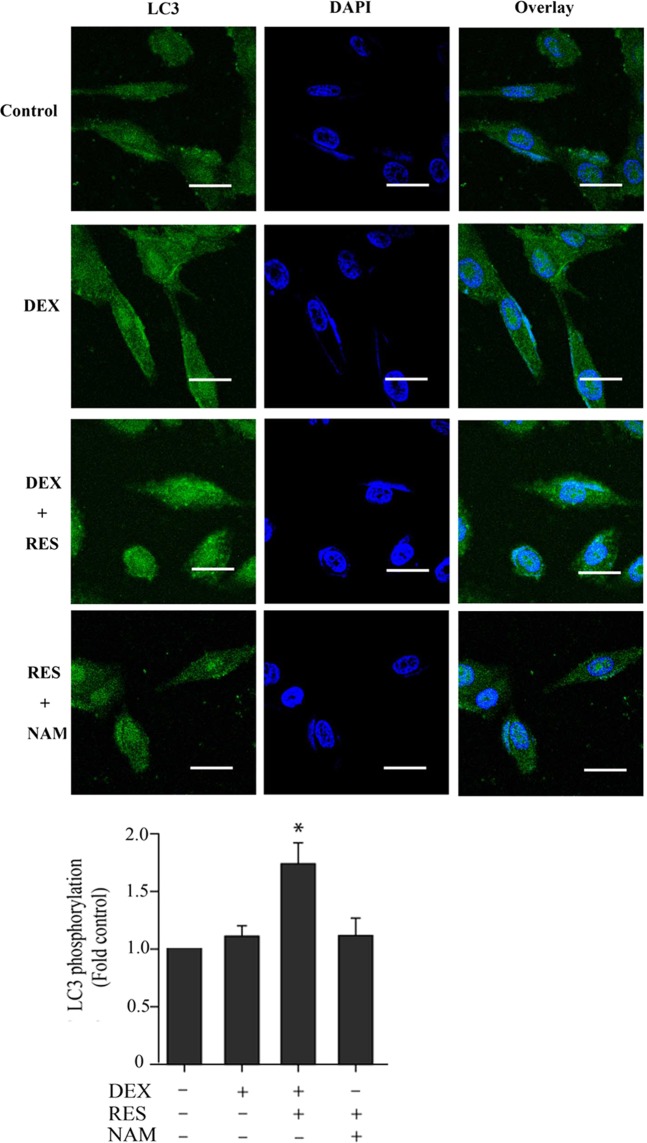
Confocal microscope analysis was performed to evaluate autophagy in cells treated with DEX, pre-incubated with RES or pre-incubated with NAM. Representative images of fluorescent LC3 puncta are shown.

**Figure 6:**
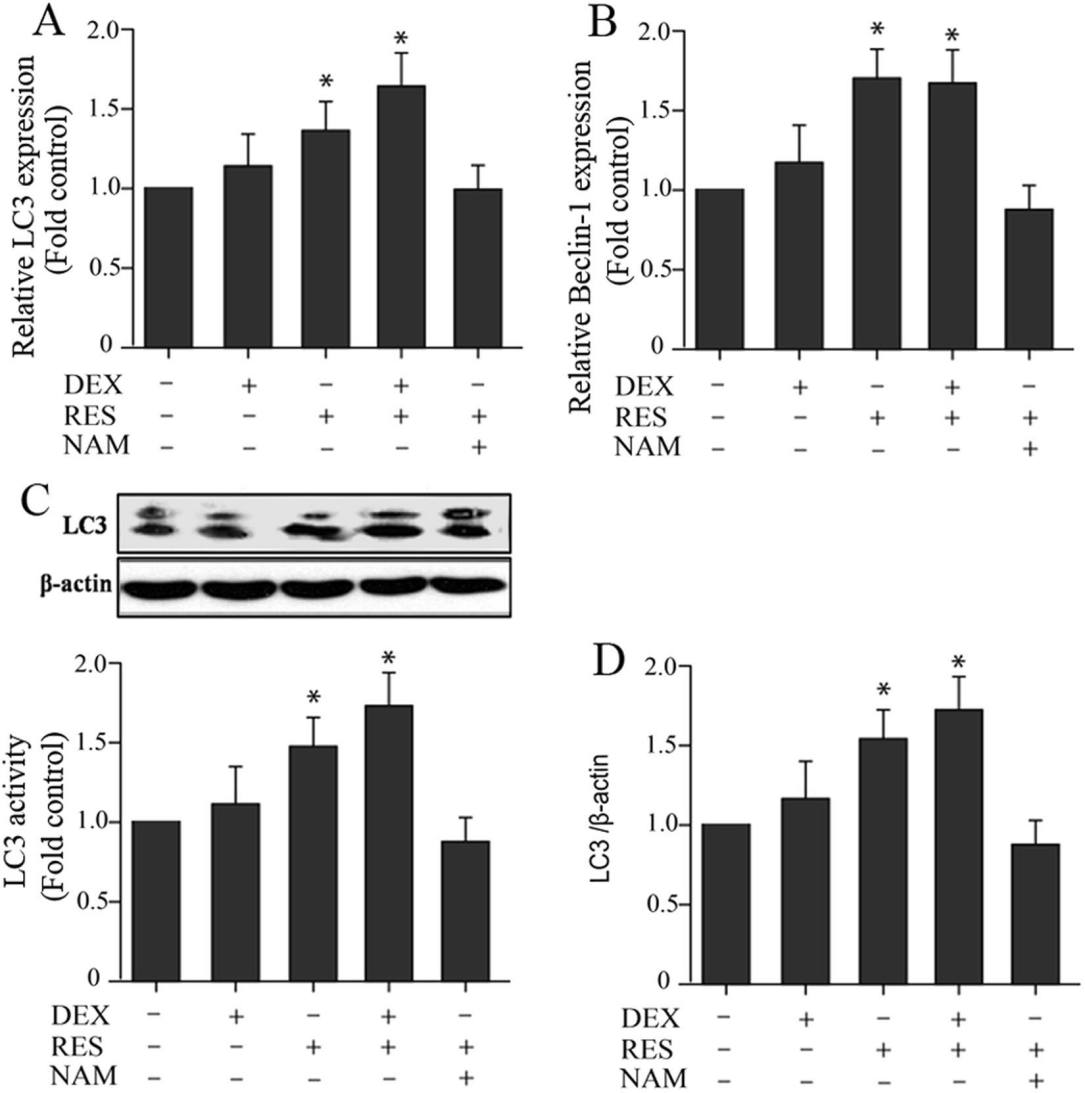
Assessment of autophagy in osteoblast induced by DEX and RES. (**A**) The LC3 mRNA levels were detected using RT-PCR assay. (**B**) The Beclin-1 mRNA levels were detected using RT-PCR assay. (**C**) The LC3 activity of treated cells were detected using Western blot assay. β-actin was used as an reference gene. (**D**) Results of densitometric analysis for Western blot.

Confocal microscope analysis showed that DEX + RES group has more the percentage of LC3-positive cells when compared to the DEX group (Fig. 5). The RT-PCR assay analysis illustrated that there was a remarkably increased expression of LC3 mRNA and Beclin1 mRNA levels in the RES group with or without exposure to DEX (Fig. 6A,B). Western blot analysis of LC3 activity and protein levels were consistent with the above results (Fig. 6C,D). These results demonstrated that resveratrol increased SIRT1 expression and may protect the viability of DEX-treated osteoblasts by inducing autophagy.

### Resveratrol up-regulated mitophagy through mediate the PI3K/Akt/mTOR pathway in DEX-treated osteoblasts

**Figure 7:**
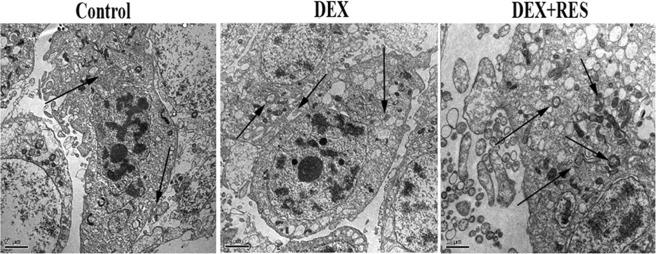
Transmission electron microscopy (TEM) showing mitophagosomes in osteoblasts. Transmission electron microscopy images indicating double membrane vacuoles in osteoblasts treated with10^−6^ M DEX with or without the presence of RES for 24 h. The vacuoles are indicated with arrows.

**Figure 8:**
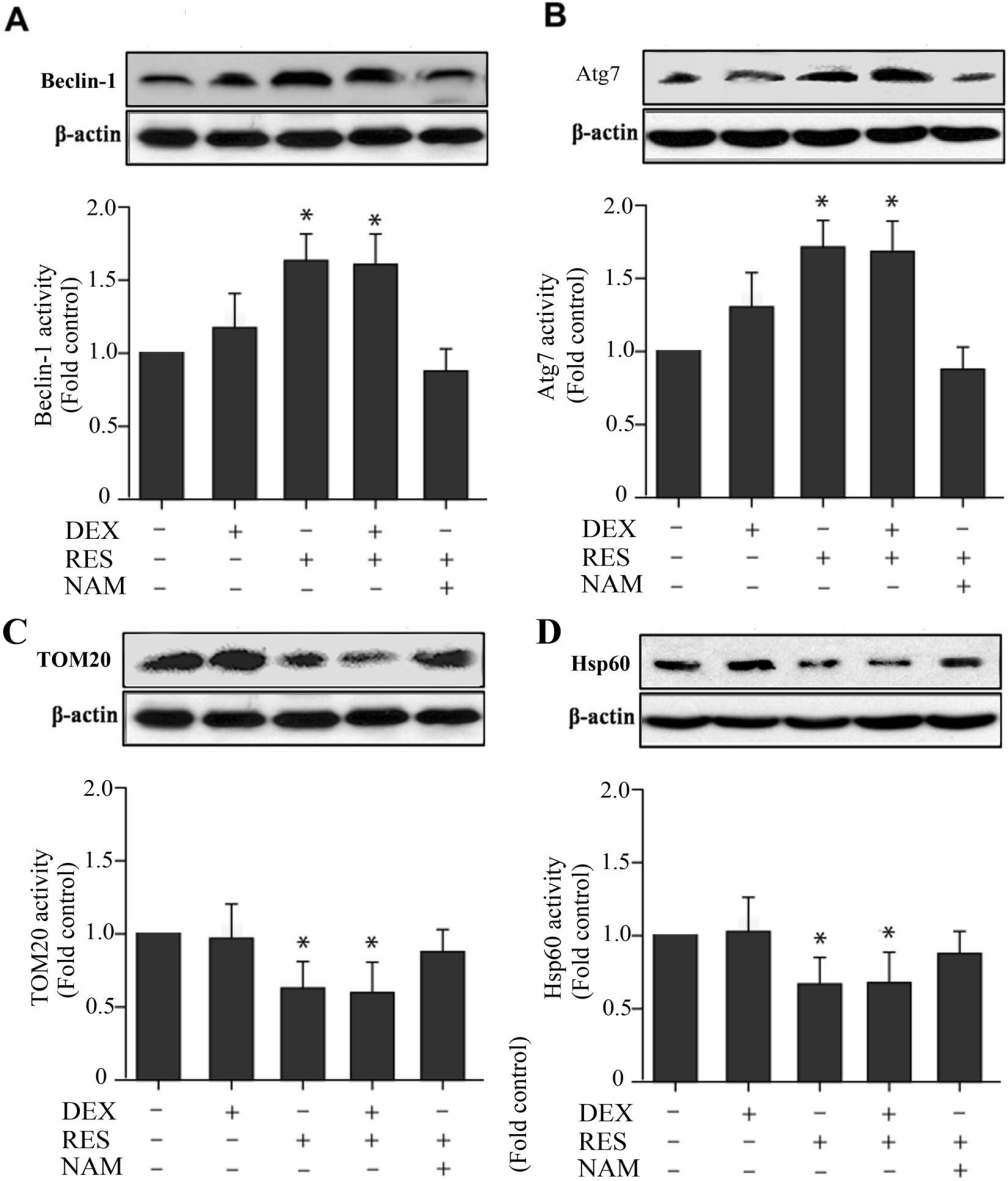
Assessment of autophagy in osteoblast induced by DEX and RES. The Beclin-1 **(A)**, Atg7 **(B)**, TOM20 **(C)** and Hsp60 **(D)** protein levels of treated cells were detected using Western blot assay.

Autophagy was assessed by morphology of the formation of autophagic vacuoles, also known as autophagosomes. TEM (Fig. 7) illustrated that there was more mitophagomes vacuoles in the DEX + RES group than the Dex group. We detected Beclin-1 and Atg7 protein levels of the treated cells by using Western blot assay. The expression level of Beclin-1 and Atg7 were significantly increased in the RES group with or without exposure to DEX when compared with the DEX group (Fig. 8A,B). For further validation, we detected TOM20 (translocase of outer mitochondrial membrane 20, a marker of mitophagy) and Hsp60 (mitochondria matrix, a marker of mitophagy) protein levels of treated cells by using Western blot assay. The levels of TOM20 and Hsp60 in the group of DEX + RES treated were lower when compared to DEX group (Fig. 8C,D). The data of this study reveals that resveratrol protect DEX-treated osteoblasts through regulate mitophagy.

**Figure 9:**
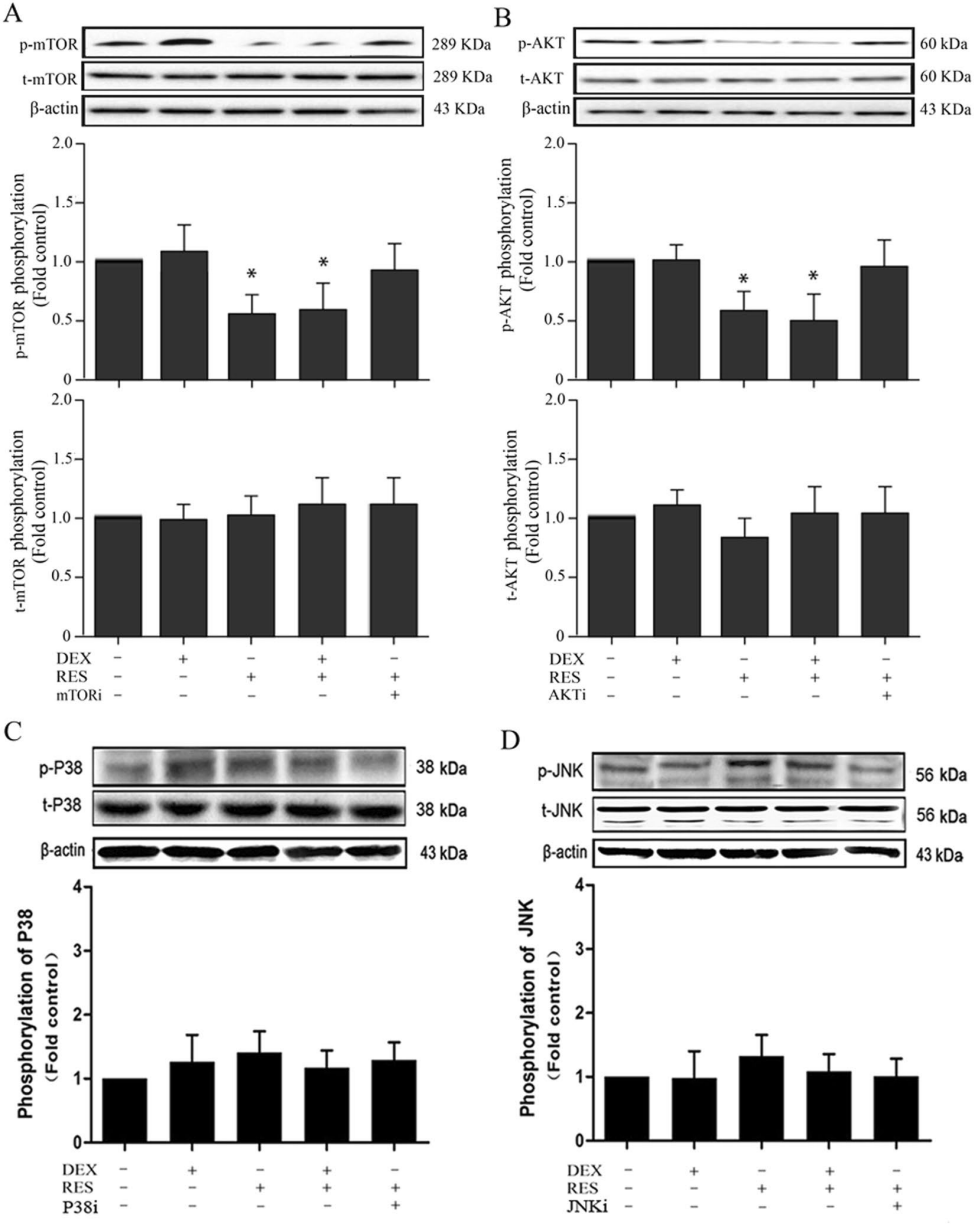
Western blot shows expression of *p*-mTOR, *p*-AKT, *p*-p38, and *p*-JNK in osteoblasts with different treatments. **(A)** Western blot shows the expression of *p*-mTOR in osteoblasts among different groups. **(B)** Western blot shows the expression of *p*-AKT in osteoblasts among different groups. **(C)** Western blot shows the expression of *p*-p38 in osteoblasts among different groups. **(D)** Western blot shows the expression of *p*-JNK in osteoblasts among different groups. Asterisks indicate significant differences compared to control (*P < 0.05).

To investigate which pathway resveratrol mediated SIRT1-induced mitophagy activation with DEX-treated of osteoblasts, we concentrated on the PI3K/Akt/mTOR signaling pathway because of its essential role in regulating autophagy. The phosphorylation of Akt has been widely accepted as one of the indicator of PI3K/Akt pathway activation. The western blot results (Fig. 9A,B) illustrated that Akt phosphorylation and mTOR phosphorylation were down-regulated in the RES treated groups with or without exposure to DEX when compared to the DEX group. However the expression of *t*-Akt and *t*-mTOR did not significantly change among the groups. Rapamycin (mTOR inhibitor) and LY294002 (PI3K inhibitor) attenuated the effects of resveratrol on both Akt phosphorylation and mTOR phosphorylation in osteoblasts, which suggested that autophagic activity was blocked. Mitogen-activated protein kinases (MAPKs), including those of the JNK and p38 signaling pathways, have also been linked to the regulation of autophagy. To further determine whether resveratrol could protect DEX-treated osteoblast through the JNK/MAPK or p38/MAPK pathways, serum-free osteoblasts were treated with DEX with or without resveratrol, with or without SP600125 (the JNK inhibitor), and with or without SB203580 (the p38 inhibitor) for 24 hours. The data indicated no changes in p38 or JNK phosphorylation among the groups (Fig. 9C,D). These results demonstrate that the resveratrol enhances the SIRT1 expression of DEX-treated osteoblasts and up-regulates mitophagy through mediate the PI3K/Akt/mTOR pathway.

## Discussion

The data indicate that resveratrol possess numerous beneficial effects, such as extending the lifespan, improving mitochondrial function, and reducing the risk of numerous degenerative diseases.

It is noticeable that SIRT1 is one of the important regulators in both cellular defense against oxidative stress and the cellular survival, and can also be activated by resveratrol to maintain mitochondrial function. Osteoporosis associated with aging can be characterized by consistent changes in bone metabolism with inhibition of bone formation, and increased bone resorption^40^. The increased oxidative stress related to aging is considered to be another key factor causing osteoporosis. Osteoblasts are the important functional cells for bone formation. What’s more, osteoblasts can produce extracellular matrix proteins and matrix mineralization regulators that are involved in early bone formation and late bone remodeling. *In vitro* studies show that pharmacological induction of autophagy in osteoblast-like cells reduces their oxidative stress and inhibits apoptosis in these cells^41^. Moreover, the age-related bone mass loss, which related to a progressive increase in the bone level of ROS, is also related to the SIRT1 activity in peripheral blood of the osteoporotic patients^42^.

Postmenopausal osteoporosis is characterized by osteolysis due to the activation of the number and function of osteoclasts, while the glucocorticoid-induced osteoporosis is characterized by reduced bone formation due to impaired osteoblast function. So in the vivo experiments, the dexamethasone-treated rats were done to simulated osteoporosis status *in vivo*, and the significant reduction of BMD was observed in the osteoporosis rats, which indicating a successful osteoporosis model. Meanwhile, it was verified that treatment with resveratrol markedly increased the BMD in osteoporosis rats, as well as the expression of SIRT1. Our vitro results showed that SIRT1 was expressed in osteoblasts, and resveratrol treatment enhanced SIRT1 expression of osteoblasts in a dose-dependent and time-dependent way. What’s more, treatment of resveratrol increased the SIRT1 expression of DEX-treated osteoblasts, while the NAM abolished the effect of resveratrol of SIRT1 expression on the osteoblasts. This study reveals that the beneficial function of resveratrol might enhance the SIRT1 expression to play its physiological regulatory role in DEX-treated osteoblasts.

We further tested the changes of serum markers ALP and OC expression of the rats in different groups. ALP is a serum marker of bone formation, associated with changes in osteoblast and osteoclast activity. OC is a protein, which is positively correlated with bone formation. Both ALP and OC can be the marker proteins for bone transformation, which are closely associated with bone resorption, bone formation and bone mineralization. SIRT1 is also closely associated with bone metabolism and bone mass. SIRT1 is also involved in deacetylation between histone and non-histone lysine residues, in which histone inhibits bone formation, reduces the expression of ALP and OC in osteoblasts, and reduces the proliferation and differentiation of osteoblasts. It has been reported that resveratrol, as an activator of SIRT1, can promote the differentiation of osteoblasts, and reduce the number of bone marrow fat cells and osteoclasts. In our vivo experiment part, ALP and OC level were increased in osteoporosis rats, while significantly decreased in resveratrol treated group when compared with osteoporosis group. It implied that resveratrol may activate the expression of SIRT1 protein in osteoblasts of the osteoporosis rats to prevent bone loss via decreased bone transformation. A previous research has indicated resveratrol inhibit T_3_-induced osteocalcin synthesis in osteoblasts, which is similar with our above results^43^.

It is recognized that osteoporosis is a major cause of morbidity and mortality associated with age-related fractures. During aging, the increased oxidative stress, which autophagy can alleviate, seems to be an important factor in the major age-related bone disease osteoporosis^44–46^. The decreased of autophagy seems to promote increased oxidative stress, which leaded to bone loss, while increased of autophagy inhibits this effect^47–49^. Autophagy could either improve cell survival or induce cell apoptosis^50^. In consequence, autophagy has been recognized as a ‘double-edged sword’^51^. In the vivo experiment we found that autophagy in osteoporotic rats was significantly reduced, while autophagy of osteoblasts treated with dexamethasone was slightly increased *in vivo*. This suggests that the increase of autophagy in osteoblasts may be a protective mechanism of osteoblasts against dexamethasone stress; with the progression of osteoporosis, autophagy of osteoblasts is reduced, which further affects bone formation and bone mass.

In the aging process, current theories suggest that autophagy reduced in several tissues and this change may lead to a decrease in the ability of recycling cellular components, including defective mitochondria. Mitochondria have been the central focus of biomedical research, because they play an important role in the research of aging and the development of human pathologies^52^. Mitochondrial dysfunction can cause a range of metabolic changes, including increased production of reactive oxygen species (ROS), reduced consumption of ATP and oxygen, and lead to cell death. Autophagy is activated under hypoxia or stress to provide energy to the cells, and is an important regulator of cellular homeostasis for the removal the damaged macromolecules and organelles, including mitochondria^53^. In addition to three types of autophagy, namely macroautophagy, microautophagy and chaperone-mediated autophagy^54–58^, autophagy can also be divided into selective and non-selective autophagy according to its destiny. The current belief is that mitophagy is a form of selective autophagy which selectively eliminates aging or damaged mitochondria and contributes to mitochondrial quality control in response to stimulation or stress. Mitophagy facilitates to prevent accumulation of ROS by eliminating mitochondria^59^. Mitophagy can not only protect cells from apoptosis through removing the damaged mitochondria caused by oxidative stress, but also promote biosynthesis of new mitochondria, which is essential for cellular homeostasis. The existing literature has observed that mitophagy plays a vital role in physiological and pathological processes, including differentiation, inflammation, aging and apoptosis.

Considerable progress has been made in unpacking the mechanisms by which damaged mitochondria target autophagy and mitochondrial, quality control in preventing the functional significance of aging, neurodegenerative diseases and other pathologies^60–63^. In the *in vitro* part of this study, more mitophagosomes was observed in the DEX + RES group when compared with DEX group by transmission electron microscopy. In addition, mitophagy markers TOM20 and Hsp60 were lower in the DEX + RES group than DEX group as western blot results shown. Similarly, a recent study also reported that serum starvation of HeLa cells can induce mitophagy with LC3 increased and TOM20 decreased^64^. Autophagy is usually a self-limited process that protects cells from cell death through a variety of mechanisms, including maintenance of bioenergy homeostasis, recycling of misfolded and aggregate-prone proteins, and removal of uncoupled or permeable mitochondria. The intrinsic pathway of apoptosis is initiated by mitochondrial membrane permeabilization (MMP). If MMP is limited to a portion of the mitochondria, this will lead to selective autophagic removal of the polarized mitochondria and prevent cell death. Along with the increased of the DEX + RES treated-induced mitophagy, the proliferation and viability of osteoblasts were also increased. The SIRT1 inhibitor NAM was used for pretreatment in order to further investigate the effect of SIRT1 on mitophagy in osteoblasts. When SIRT1 is inhibited, resveratrol loses its ability to increase mitophagy. The results presented above demonstrate that resveratrol may protect DEX-treated osteoblasts through enhance SIRT1 activation to induce mitophagy.

The serine/threonine kinase Akt, is a downstream target of phosphatidylinositol 3-kinase (PI3K) in a variety of cells including osteoblasts, and is activated by several stimuli, in a PI3K-dependent manner. A previously published study has shown resveratrol increased the SIRT1 expression and activation by up-regulated the deacetylation of p53, thereby downregulated Akt phosphorylation^65^. As we all known, The PI3K/Akt/mTOR signaling pathway is implicated in a diverse array of physiological and pathological cell functions, including growth, apoptosis and autophagy^66,67^. In this study, resveratrol treatment increased SIRT1-induced mitophagy in the DEX-treated osteoblasts via PI3K/Akt/mTOR signaling pathway, which suggested that the PI3K/Akt/mTOR signaling pathway might be a target for osteoporosis therapy. As the western blot results shown, the *p*-Akt protein level was remarkably decreased in DEX + RES group, while the expression levels of the *t*-Akt protein among the groups remained unchanged. It has previously been reported that mTOR, which is positively regulated by PI3K, is a negative regulator of autophagy. In the present study, the expression level of *p*-mTOR was significantly decreased in the DEX + RES treated group when compared with the DEX group. In addition to incubation with resveratrol, osteoblasts or DEX-treated osteoblasts were incubated with an inhibitor of mTOR, an inhibitor of PI3Ki or an inhibitor of SIRT1. The results shown that the inhibition of mTOR or PI3Ki or NAM abolished the effect of resveratrol on SIRT1-induced mitophagy, which indicated that resveratrol improved mitophagy through the PI3K/Akt/mTOR signaling pathway via enhanced SIRT1 expression. Besides the PI3K/Akt signaling pathway, other MAPK signaling pathways, including JNK and p38, might also be involved in the regulation of autophagy. To verify whether resveratrol also regulated mitophagy through JNK or p38, cells were pretreated with a p38i and a JNKi for 30 min in this study. The *p*-p38 and *p*-JNK levels did not show significant differences between the experimental groups. These results indicated that SIRT1 did not regulate osteoblasts mitophagy through the JNK or p38 signaling pathways.

However, there are several remaining questions requiring further research, for example, whether the inhibition of osteoclasts was associated in the beneficial effects of resveratrol via SIRT1 activation. As the increasing prevalence of osteoporosis with an aging population, further experimental research is needed on the treatment of resveratrol on osteoporosis.

## Conclusions

Based on experimental data collected *in vivo* and vitro studies, this study reveals that resveratrol treatment protected osteoblasts in osteoporosis rats by activating the PI3K/Akt/mTOR signaling pathway through enhancing mitophagy via up-regulating the expression of SIRT1. Moreover, there seems to be a need for further clinical trials in humans which seeks to confirm the possible relationship between autophagic dysfunction and osteoporosis in order to develop future therapies for the prevention and/or treatment of osteoporosis.

## Supplementary information

Supplementary file
