# Supplementary material for: The Role and Mechanism of SIRT1 in Resveratrol-regulated Osteoblast Autophagy in Osteoporosis Rats
Source: Sci Rep. 2019 Dec 5;9:18424. doi: 10.1038/s41598-019-44766-3 (PMC6895060; doi:10.1038/s41598-019-44766-3)
Supplement: Supplementary file 1 — Supplementary file [file 41598_2019_44766_MOESM1_ESM.doc]

**Supplementary Information**

**The Role and Mechanism of SIRT1 in Resveratrol-regulated Osteoblast Autophagy in the Osteoporosis Rats**

Xuhao Yang^¶^, Tianlong Jiang ^¶^, Yu Wang, and Lei Guo*

Department of Orthopedic Surgery, First Affiliated Hospital, China Medical University, Shenyang, Liaoning, 110001, P.R. China;

***Address correspondence to**: Lei Guo, First Affiliated Hospital, China Medical University, Shenyang, Liaoning, P.R. China, Phone: +86 15241818899, E-mail: [guolei@cmu.edu.cn](mailto:guolei@cmu.edu.cn)

¶ Contributed equally to this work.

Context of supplementary information

Supplementary Figure 3, 4, 6, 8 and 9.

Figure 3


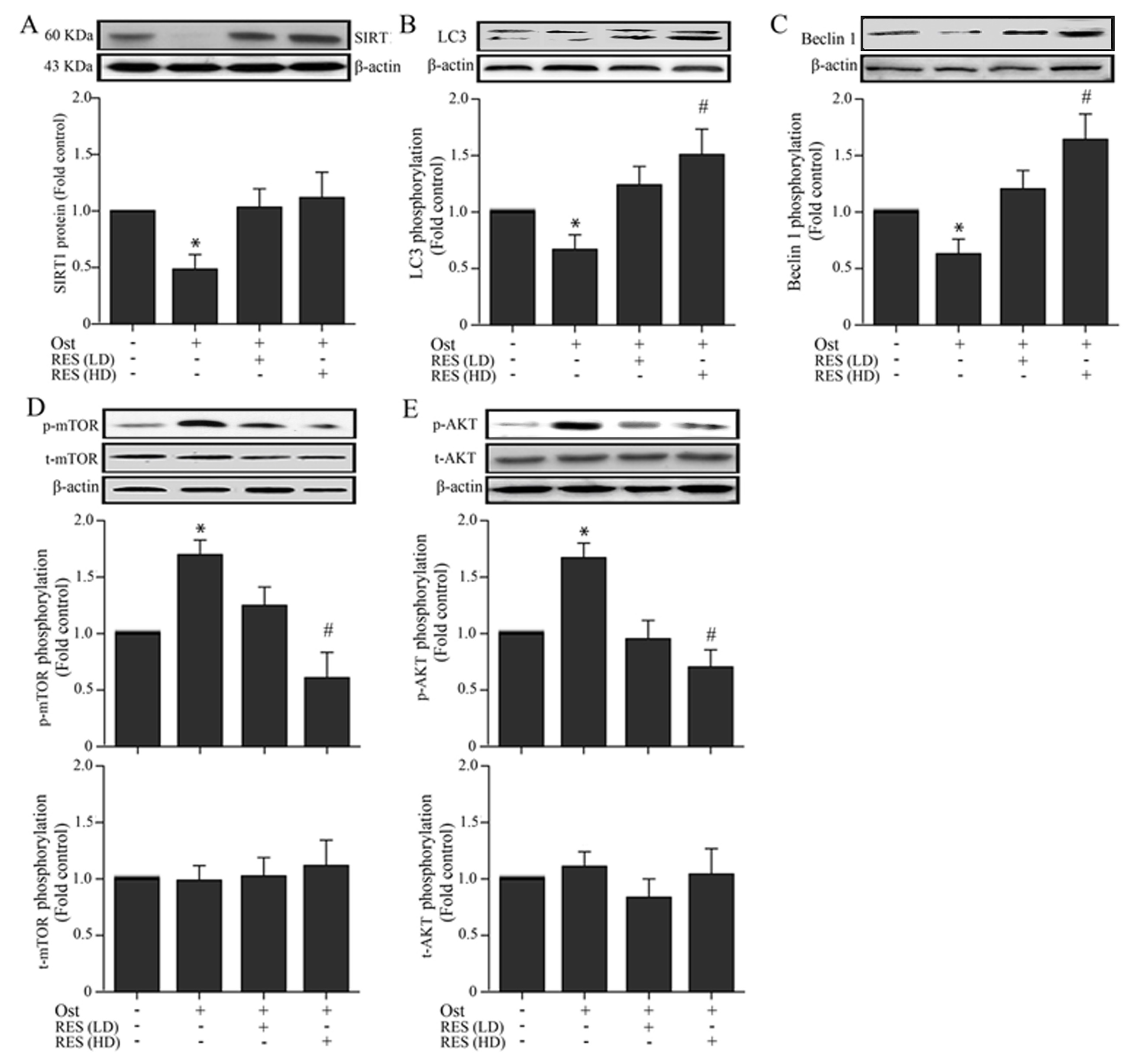


Figure 3A1


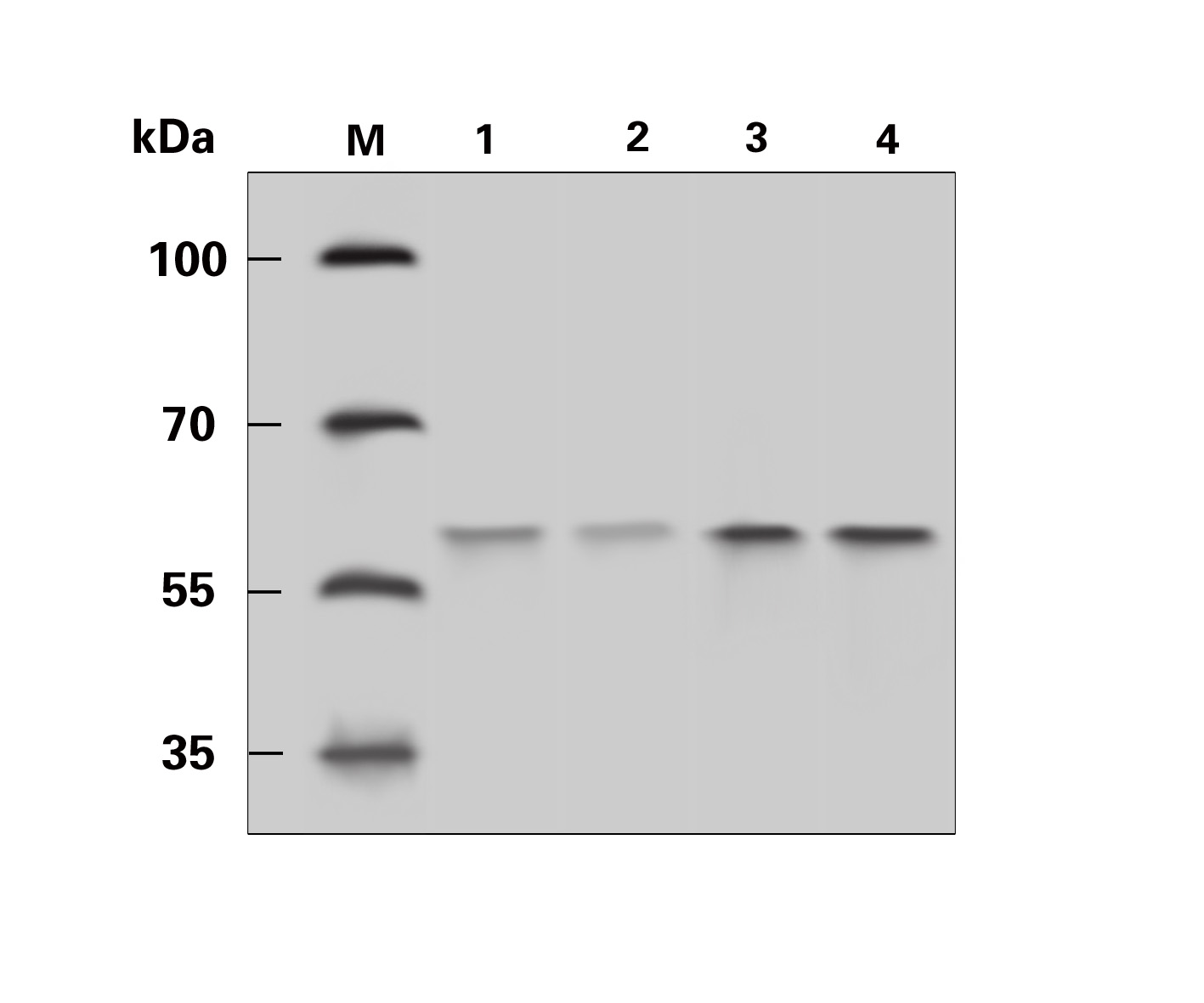


M：marker protein

1：control group

2：osteoporosis group

3: osteoporosis + resveratrol group (low dose)

4: osteoporosis + resveratrol group (high dose)

SIRT1=60kDa

Figure 3A2


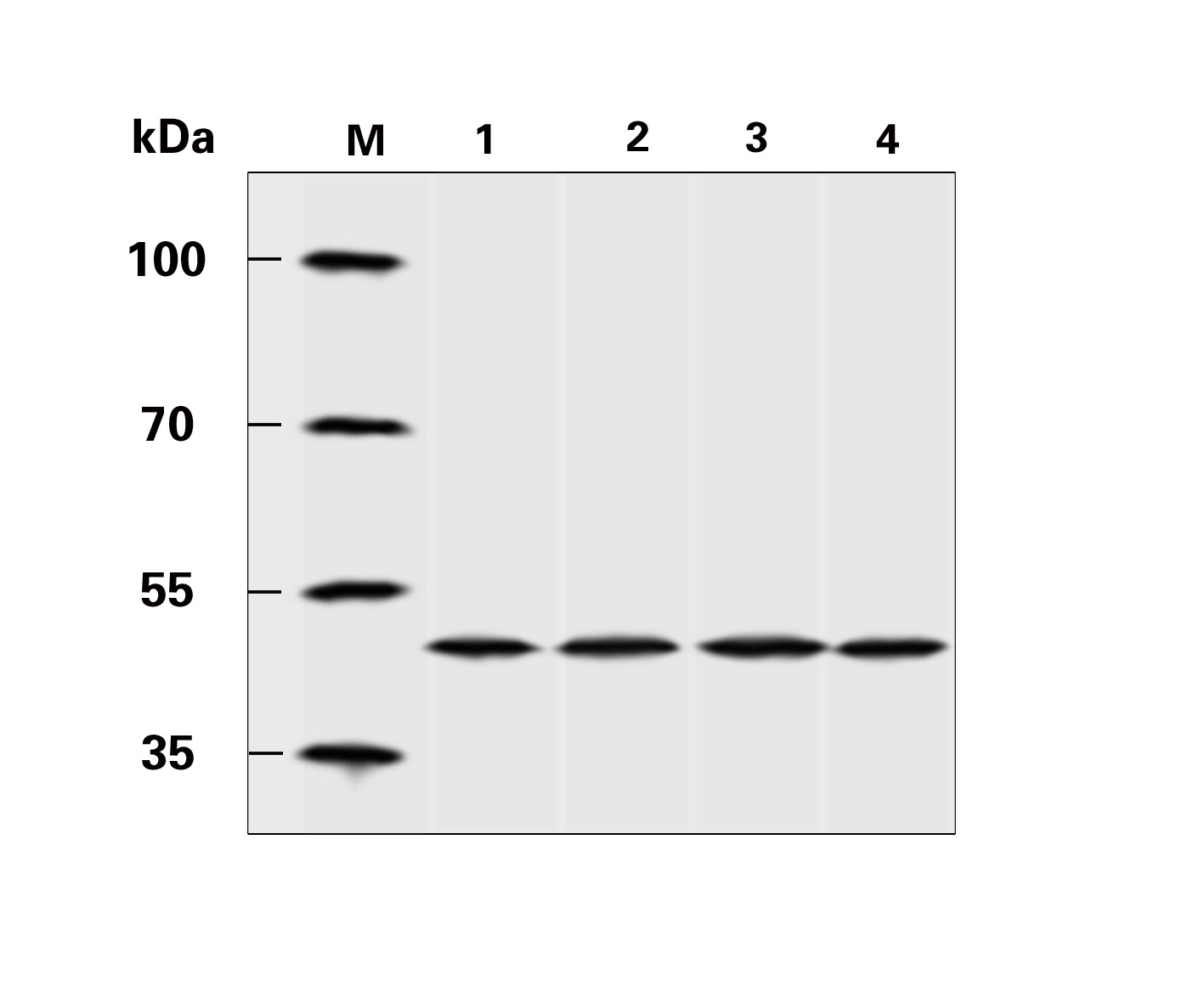


M：marker protein

1：control group

2：osteoporosis group

3: osteoporosis + resveratrol group (low dose)

4: osteoporosis + resveratrol group (high dose)

β-actin=43kDa

Figure 3B1


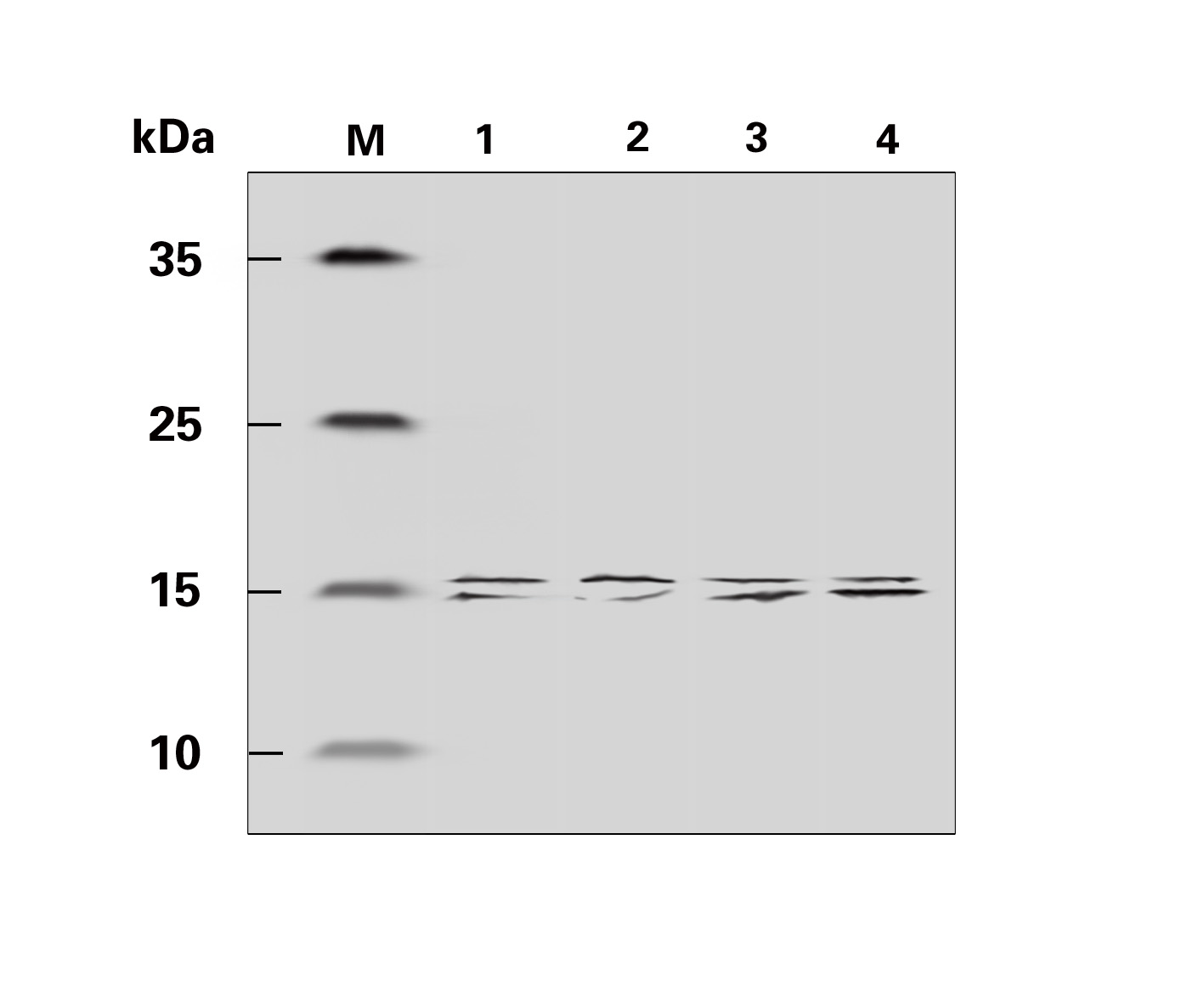


M：marker protein

1：control group

2：osteoporosis group

3: osteoporosis + resveratrol group (low dose)

4: osteoporosis + resveratrol group (high dose)

LC3-I=16kDa, LC3-II=14kDa

Figure 3B2


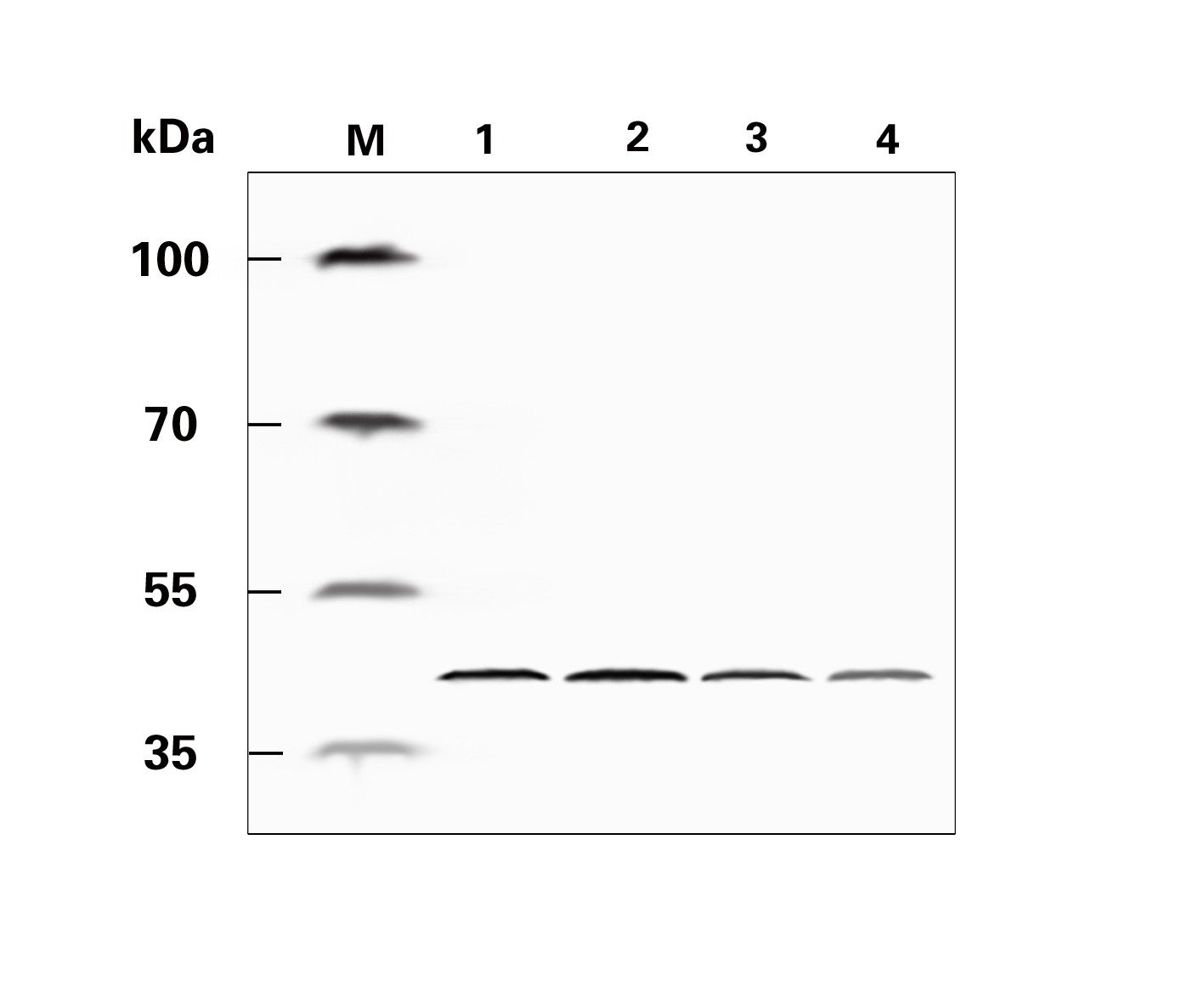


M：marker protein

1：control group

2：osteoporosis group

3: osteoporosis + resveratrol group (low dose)

4: osteoporosis + resveratrol group (high dose)

β-actin=43kDa

Figure 3C1


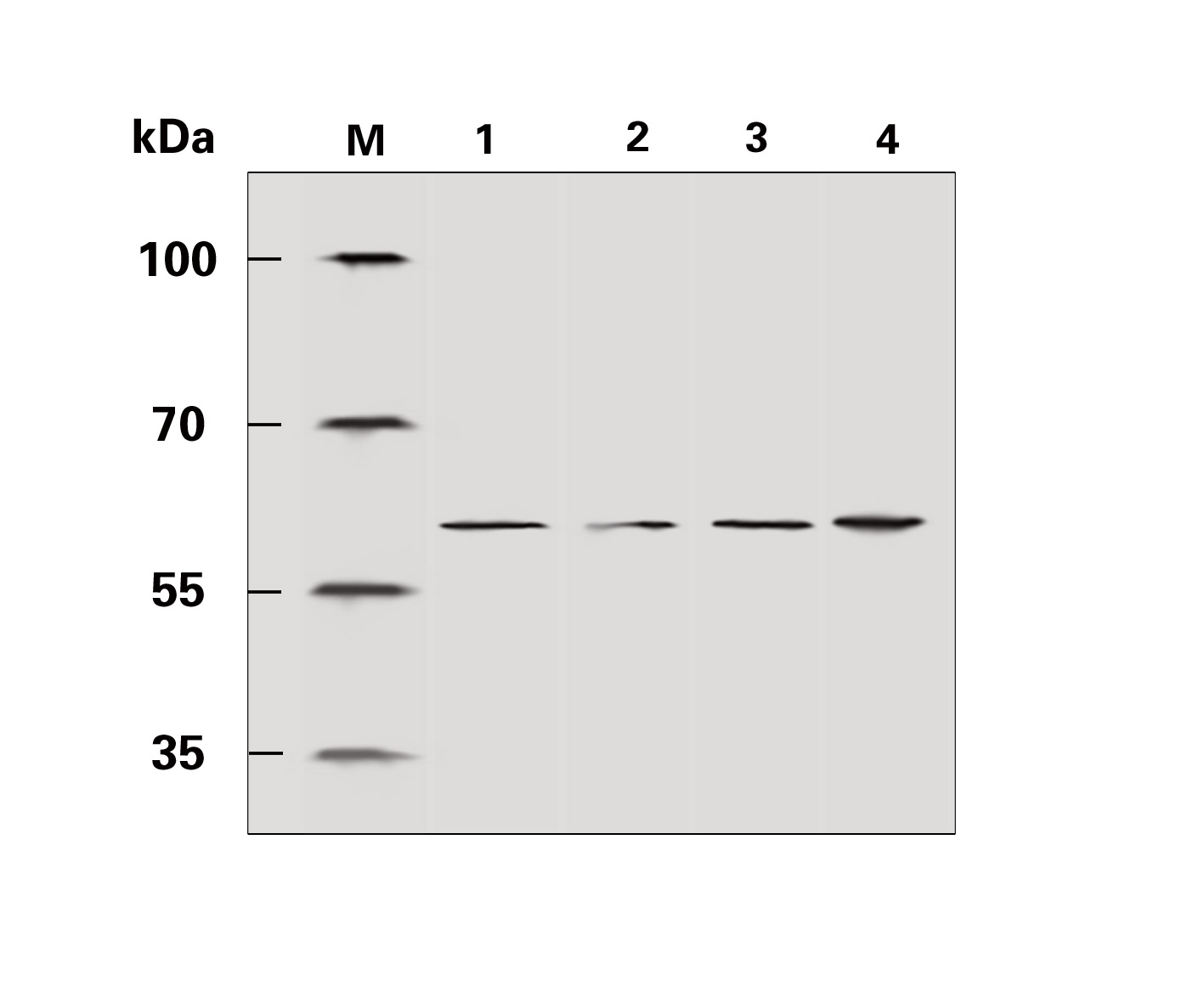


M：marker protein

1：control group

2：osteoporosis group

3: osteoporosis + resveratrol group (low dose)

4: osteoporosis + resveratrol group (high dose)

Beclin-1=60kDa

Figure 3C2


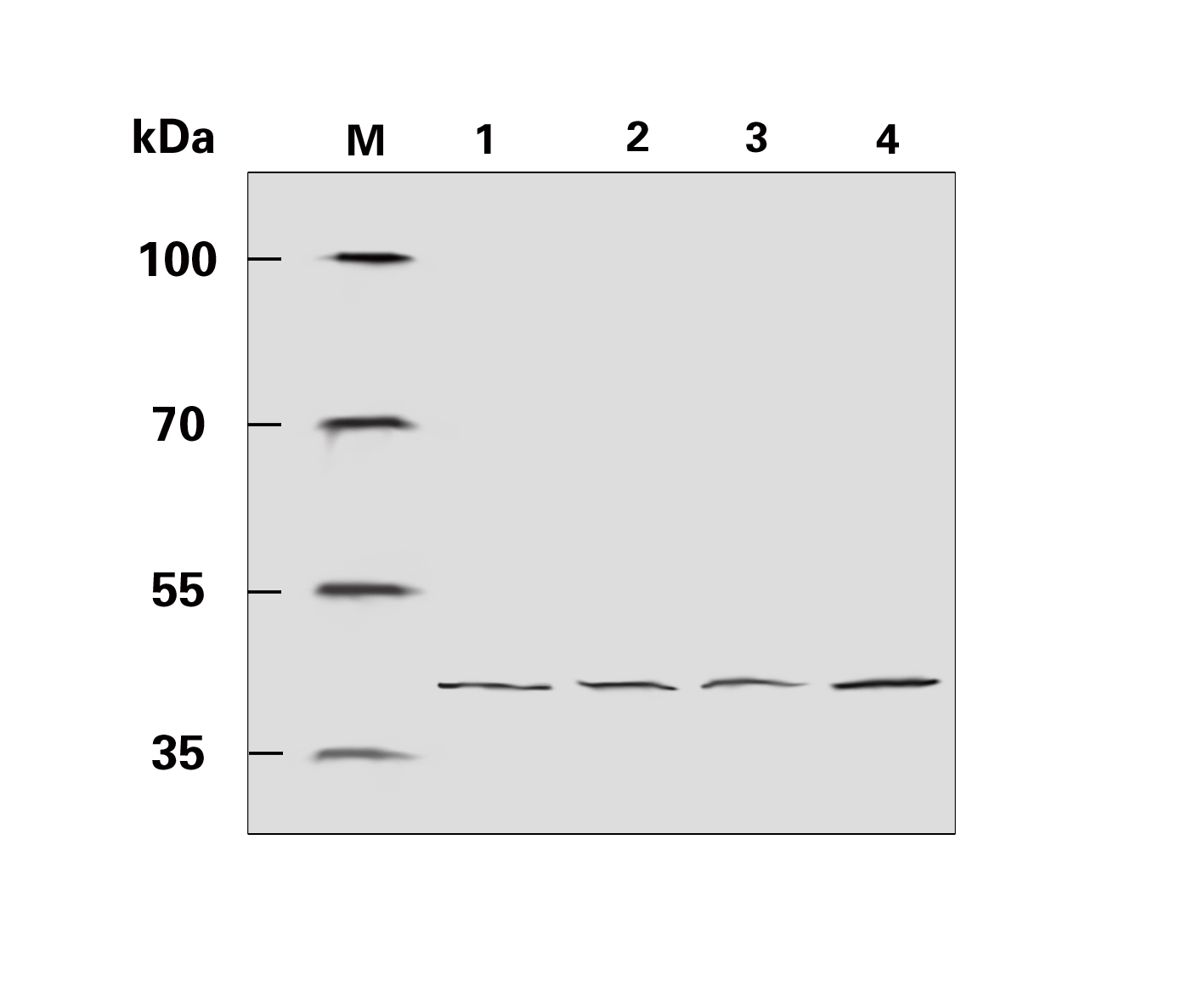


M：marker protein

1：control group

2：osteoporosis group

3: osteoporosis + resveratrol group (low dose)

4: osteoporosis + resveratrol group (high dose)

β-actin=43kDa

Figure 3D1


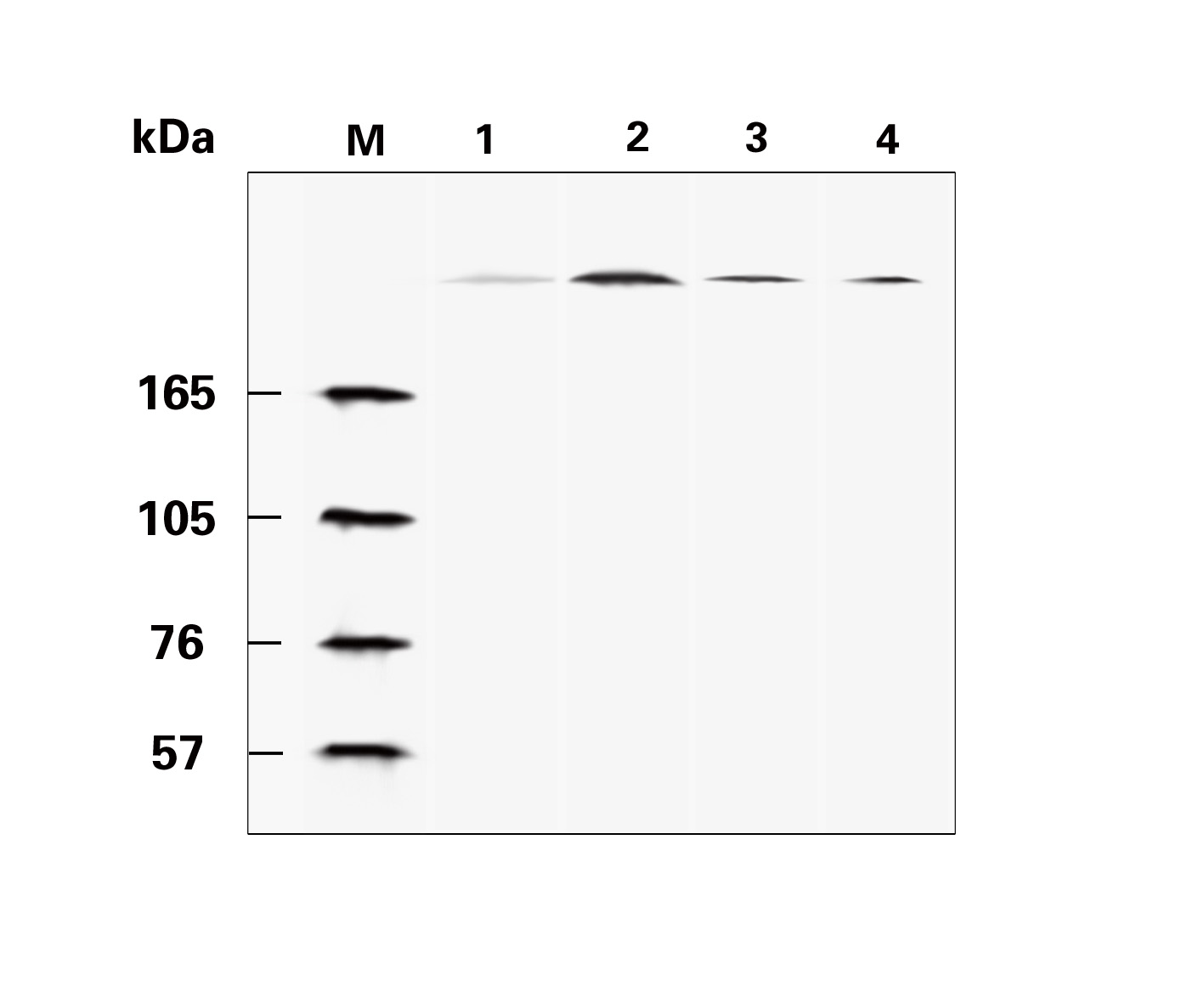


M：marker protein

1：control group

2：osteoporosis group

3: osteoporosis + resveratrol group (low dose)

4: osteoporosis + resveratrol group (high dose)

*p*-m-TOR=289kDa

Figure 3D2


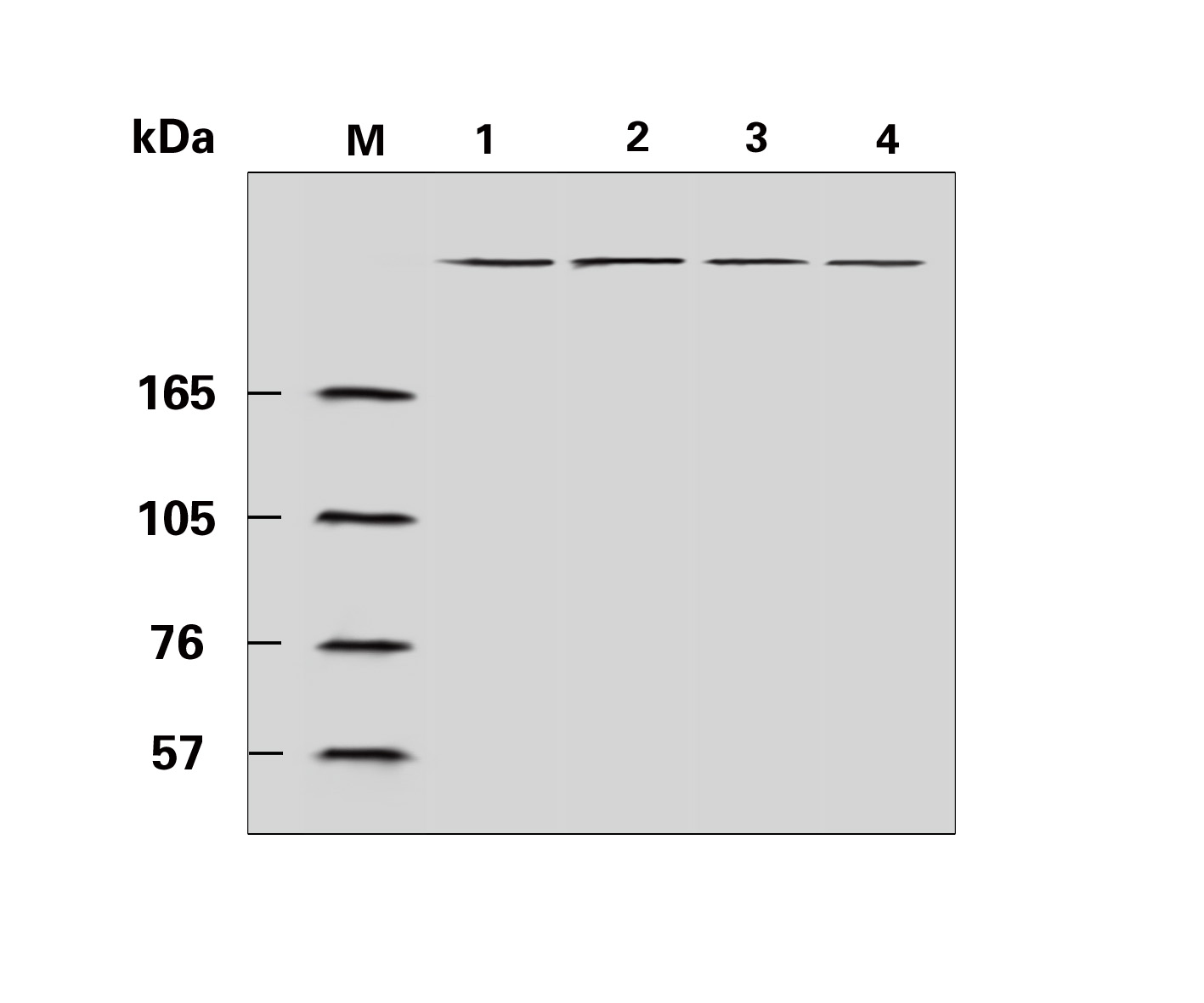


M：marker protein

1：control group

2：osteoporosis group

3: osteoporosis + resveratrol group (low dose)

4: osteoporosis + resveratrol group (high dose)

*t*-m-TOR=289kDa

Figure 3D3


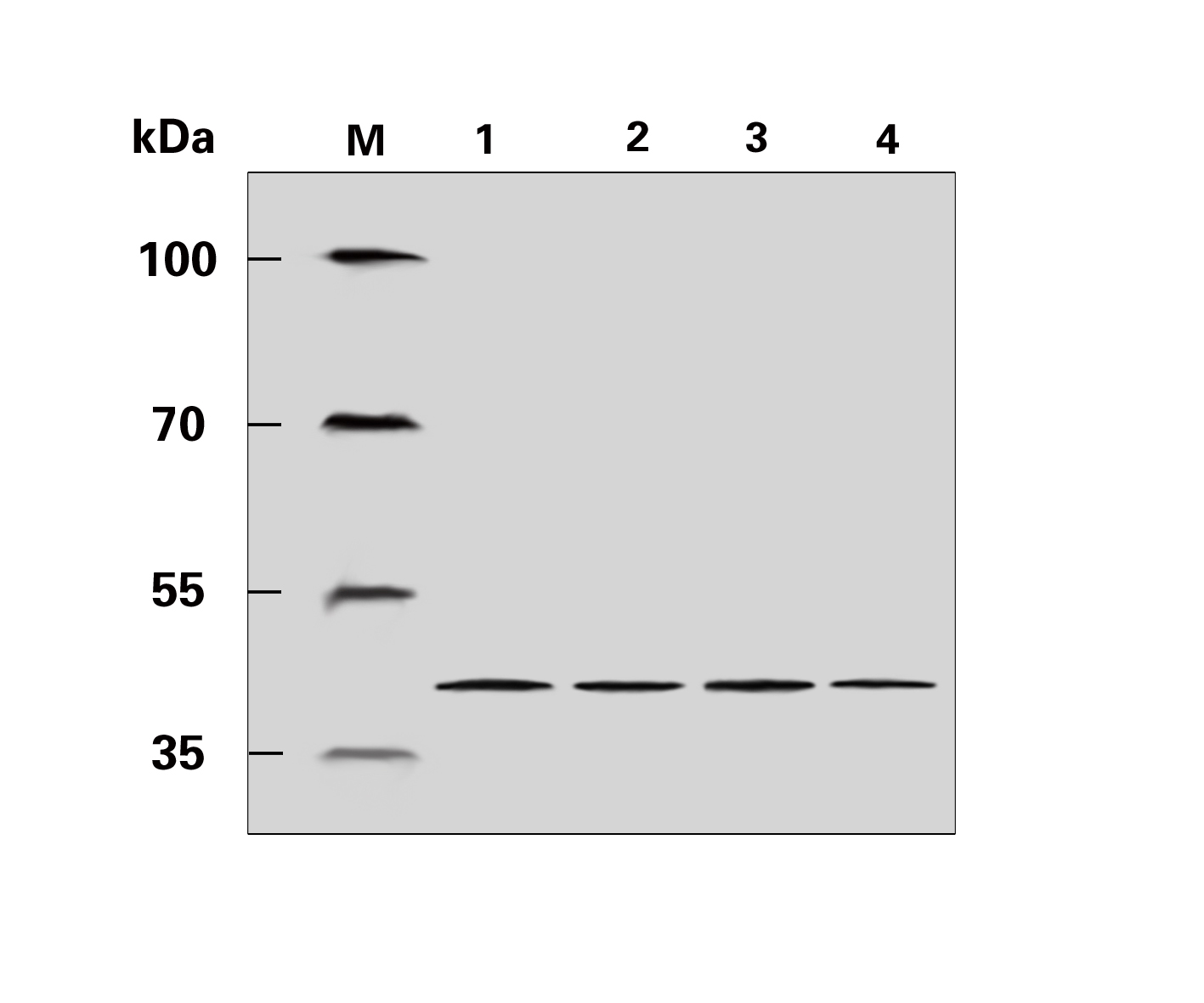


M：marker protein

1：control group

2：osteoporosis group

3: osteoporosis + resveratrol group (low dose)

4: osteoporosis + resveratrol group (high dose)

β-actin=43kDa

Figure 3E1


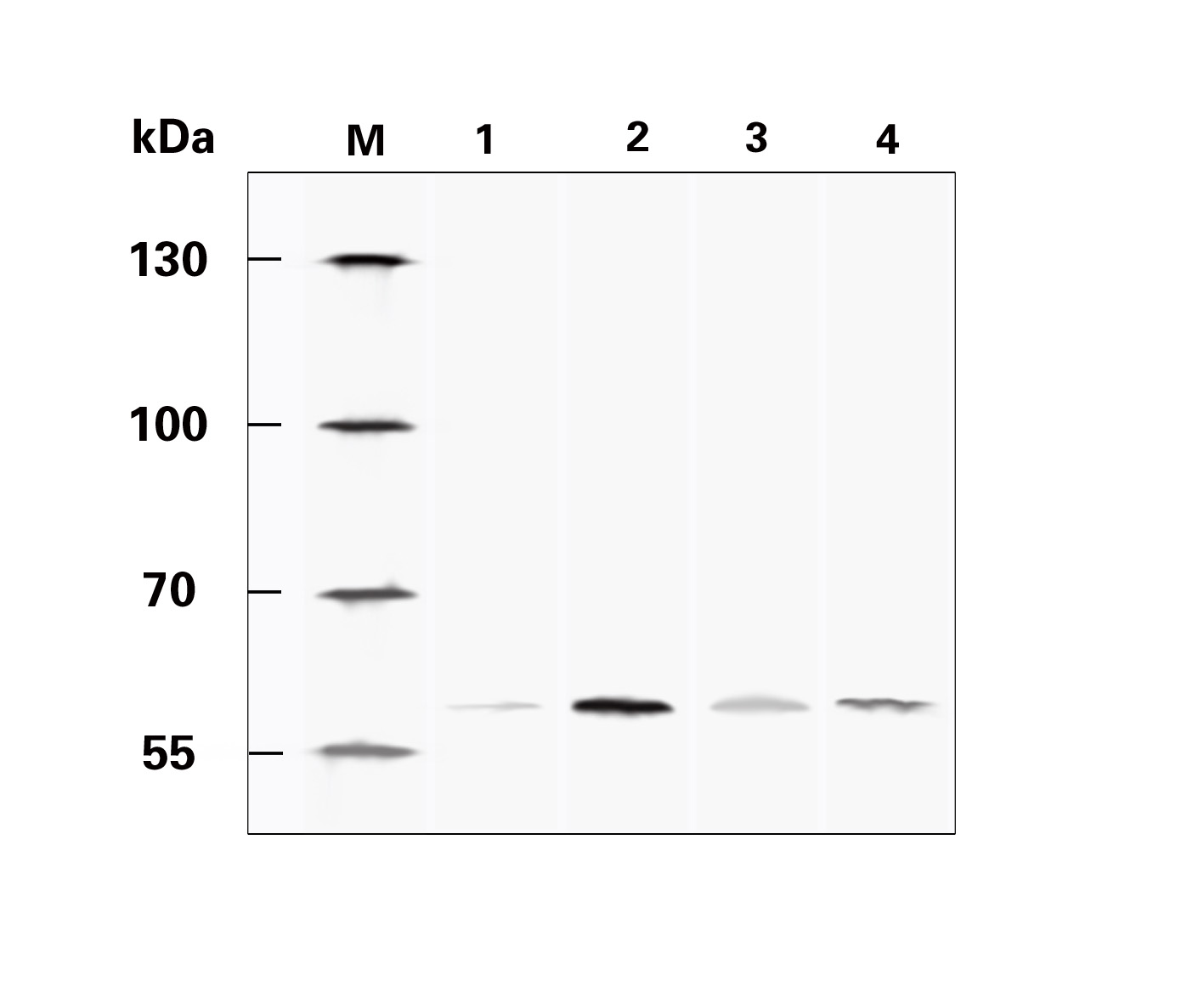


M：marker protein

1：control group

2：osteoporosis group

3: osteoporosis + resveratrol group (low dose)

4: osteoporosis + resveratrol group (high dose)

*p*-Akt=60kDa

Figure 3E2


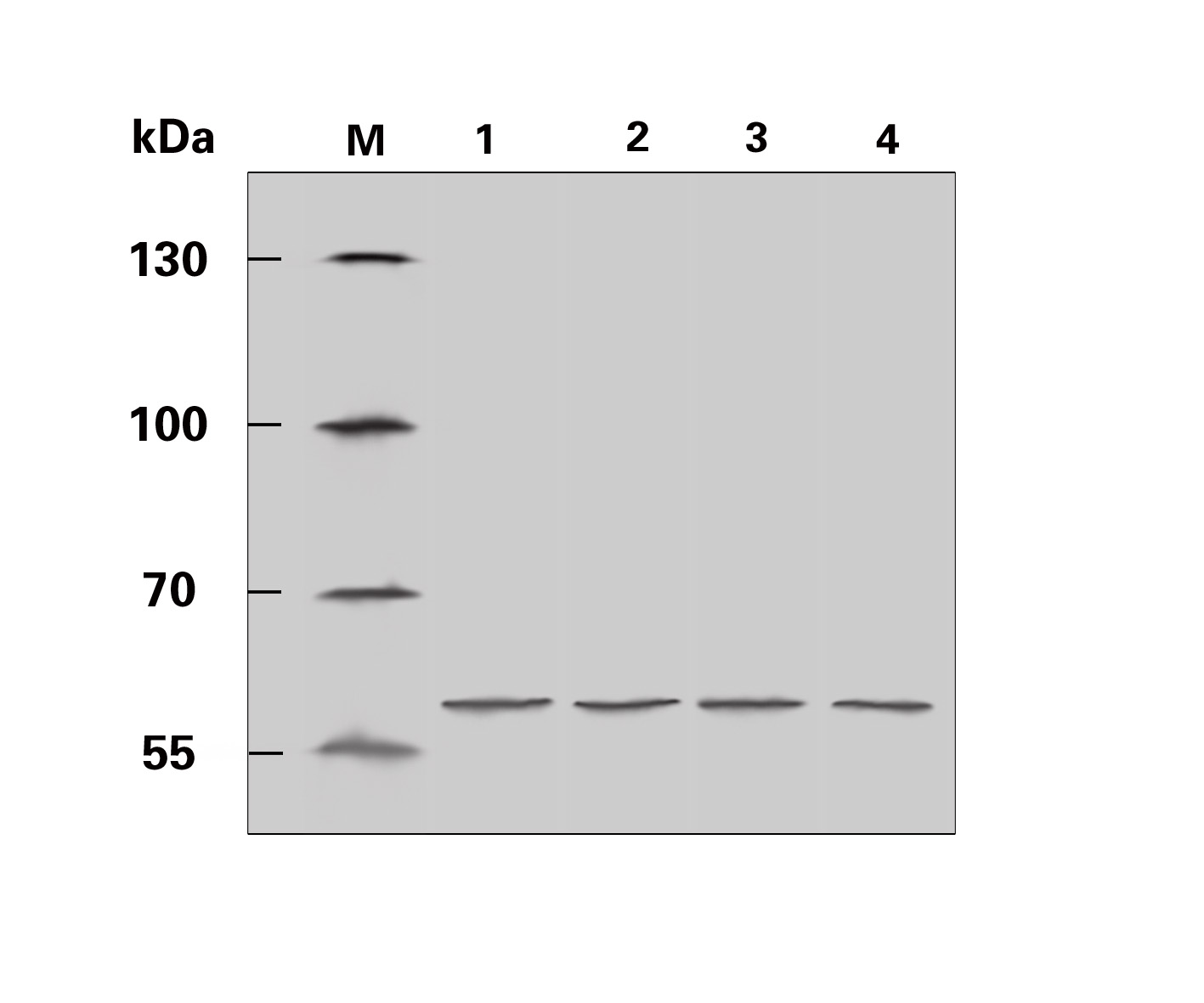


M：marker protein

1：control group

2：osteoporosis group

3: osteoporosis + resveratrol group (low dose)

4: osteoporosis + resveratrol group (high dose)

*t*-Akt=60kDa

Figure 3E3


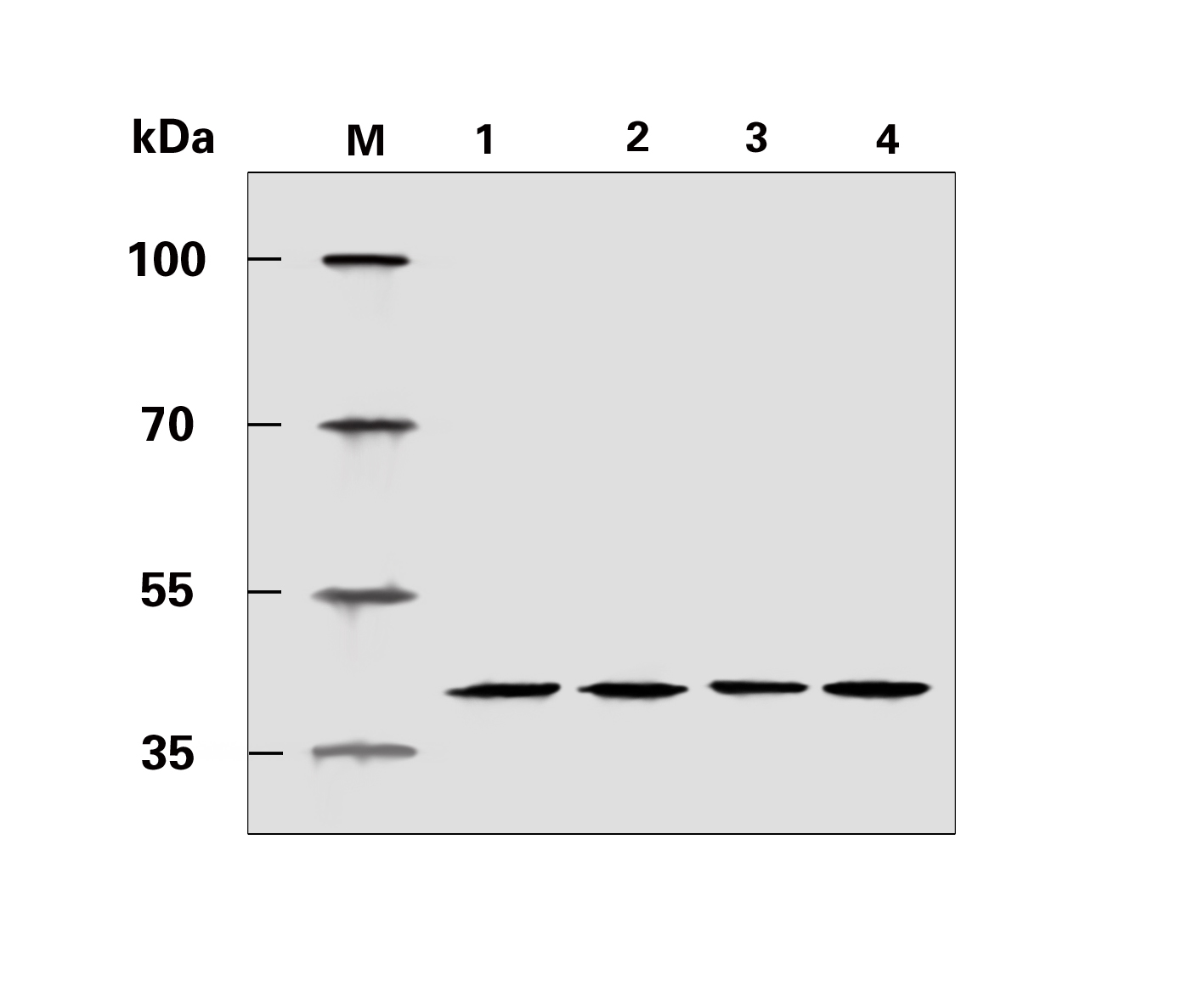


M：marker protein

1：control group

2：osteoporosis group

3: osteoporosis + resveratrol group (low dose)

4: osteoporosis + resveratrol group (high dose)

β-actin=43kDa

Figure 4


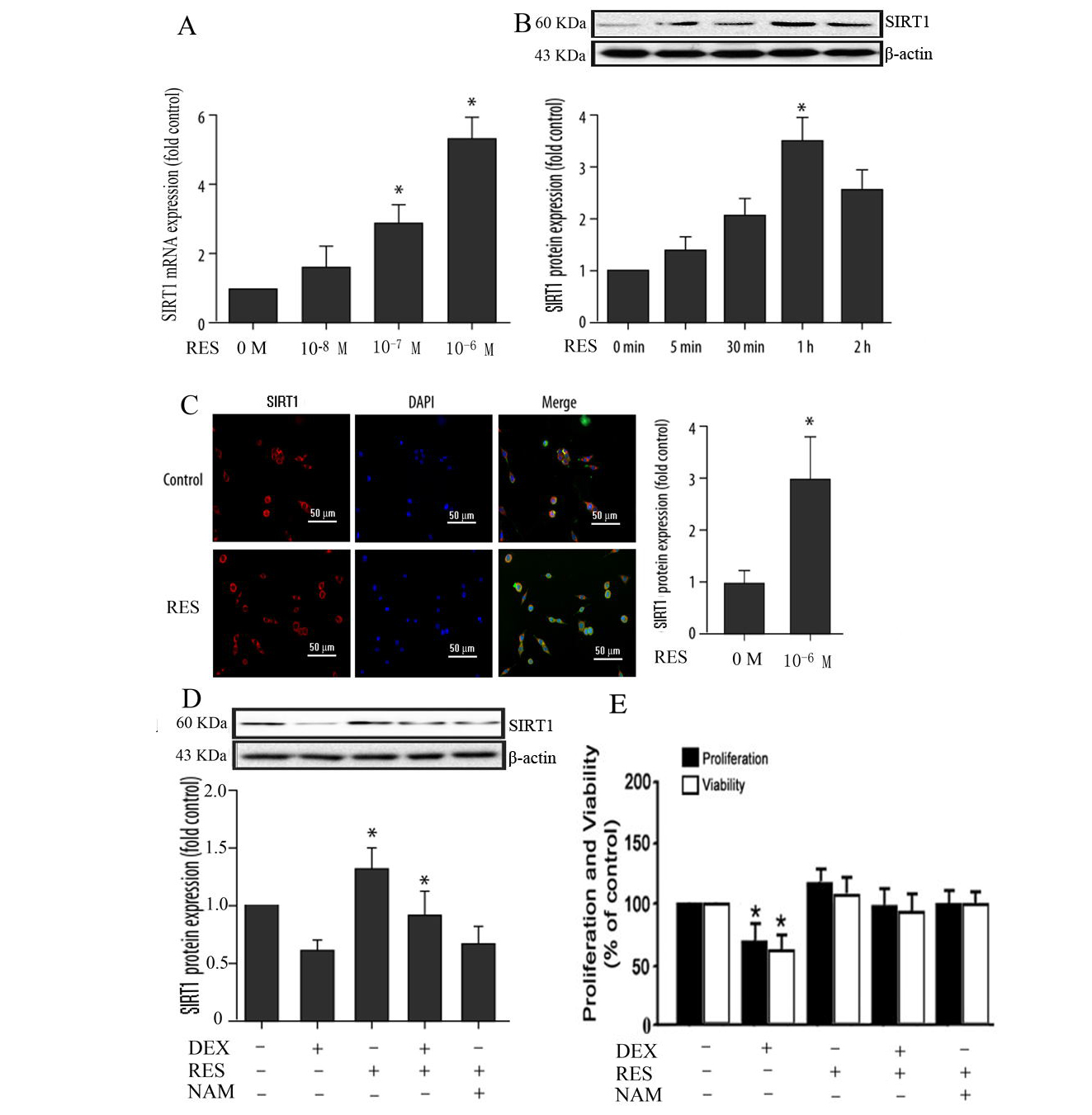


Figure 4B1


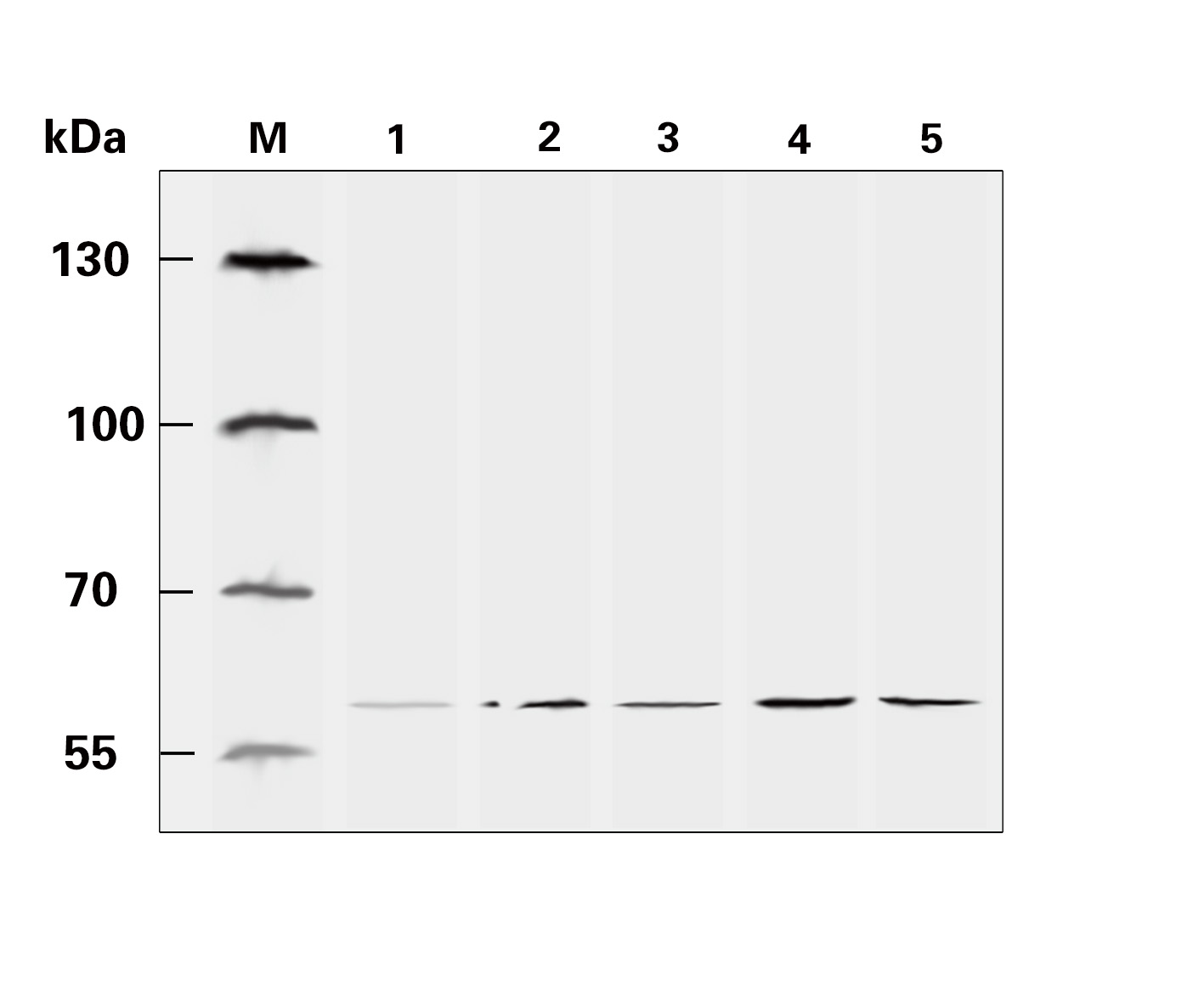


M：marker protein

1：0min group

2：5min group

3: 30min group

4: 60min group

5:120min group

SIRT1=60kDa

Figure 4B2


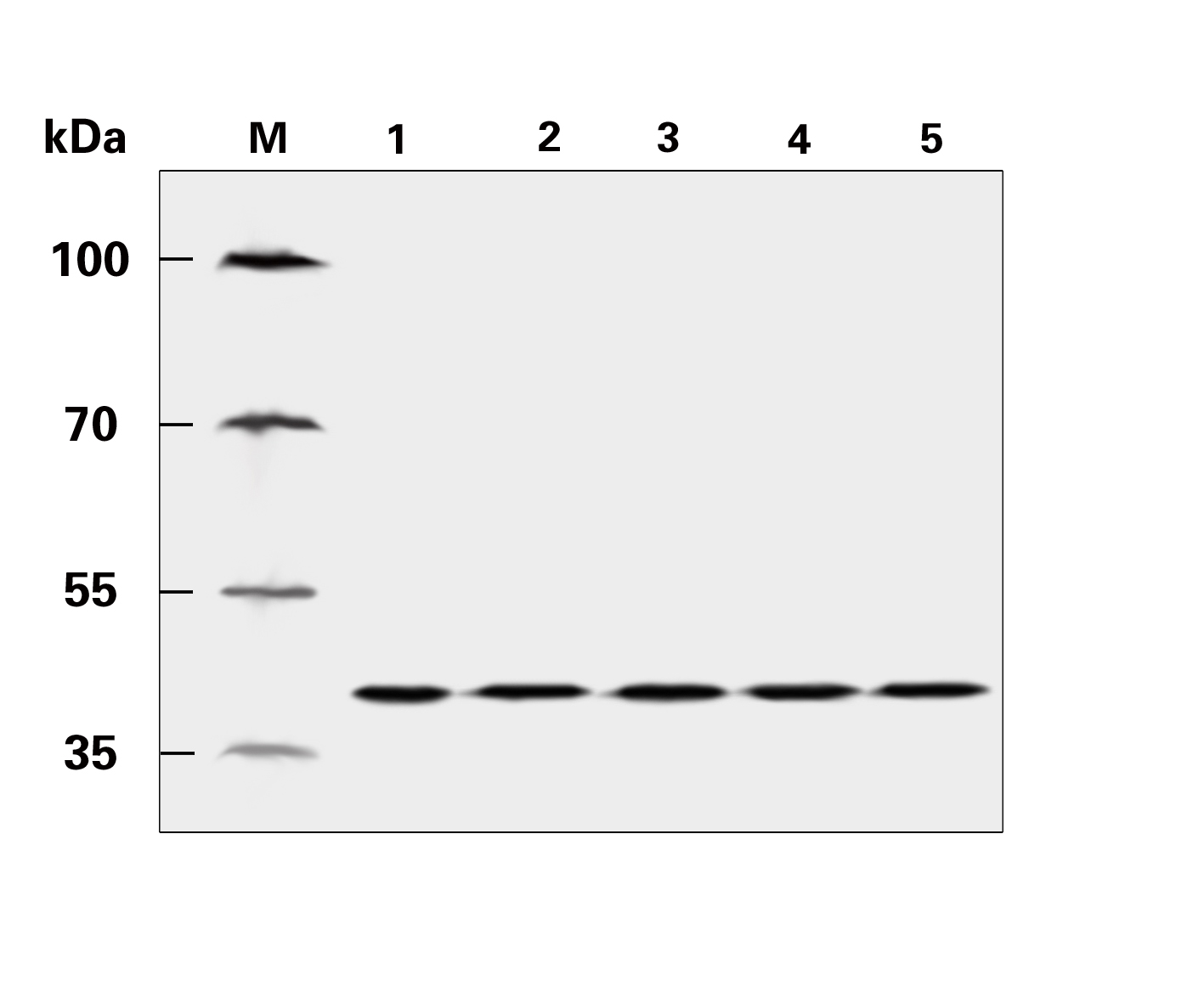


M：marker protein

1：0min group

2：5min group

3: 30min group

4: 60min group

5:120min group

β-actin=43kDa

Figure 4D1


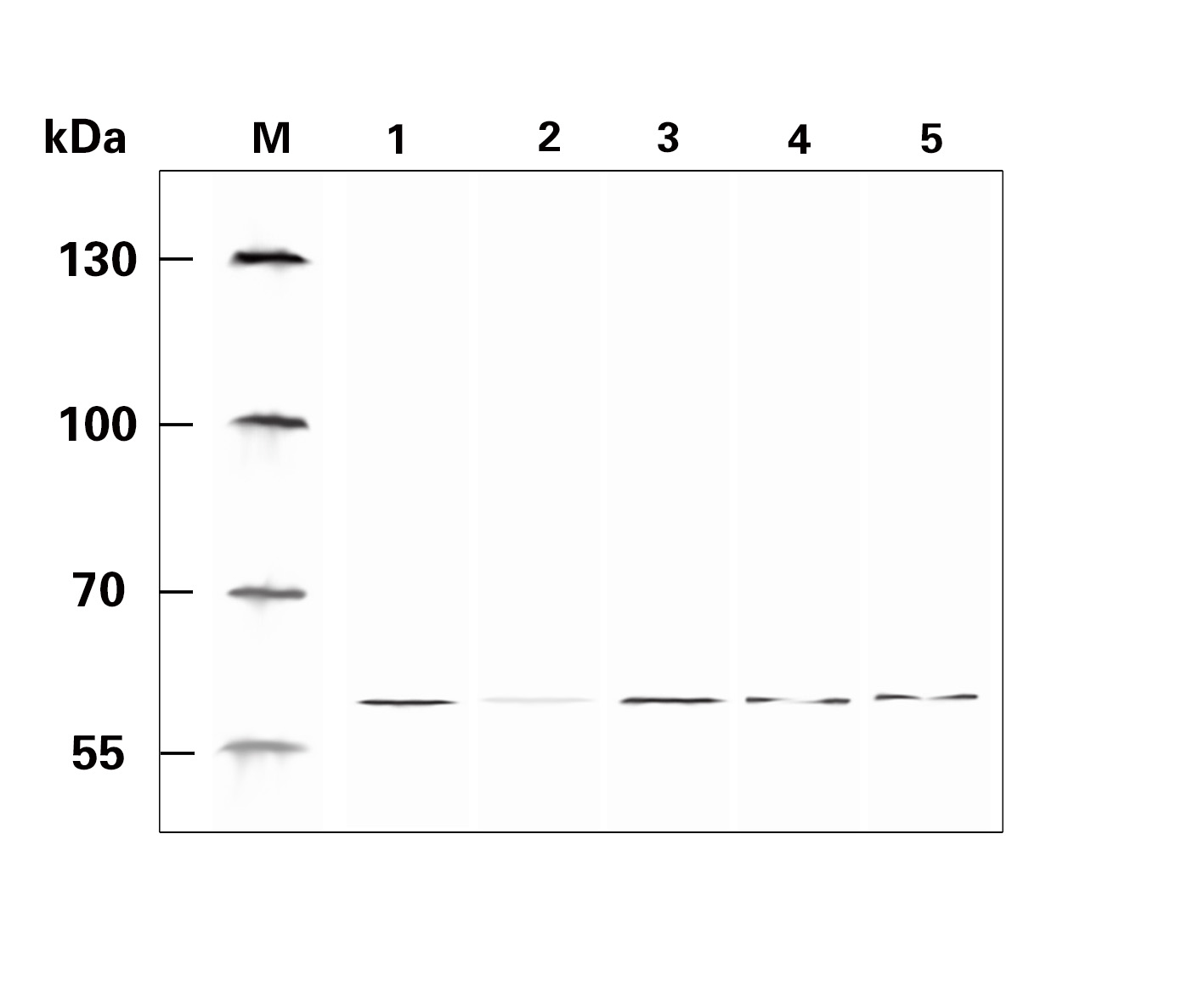


M: marker protein

1: control group

2: Dex group

3: Res group

4: Dex+Res group

5: Res+NAM group

SIRT1=60kDa

Figure 4D2


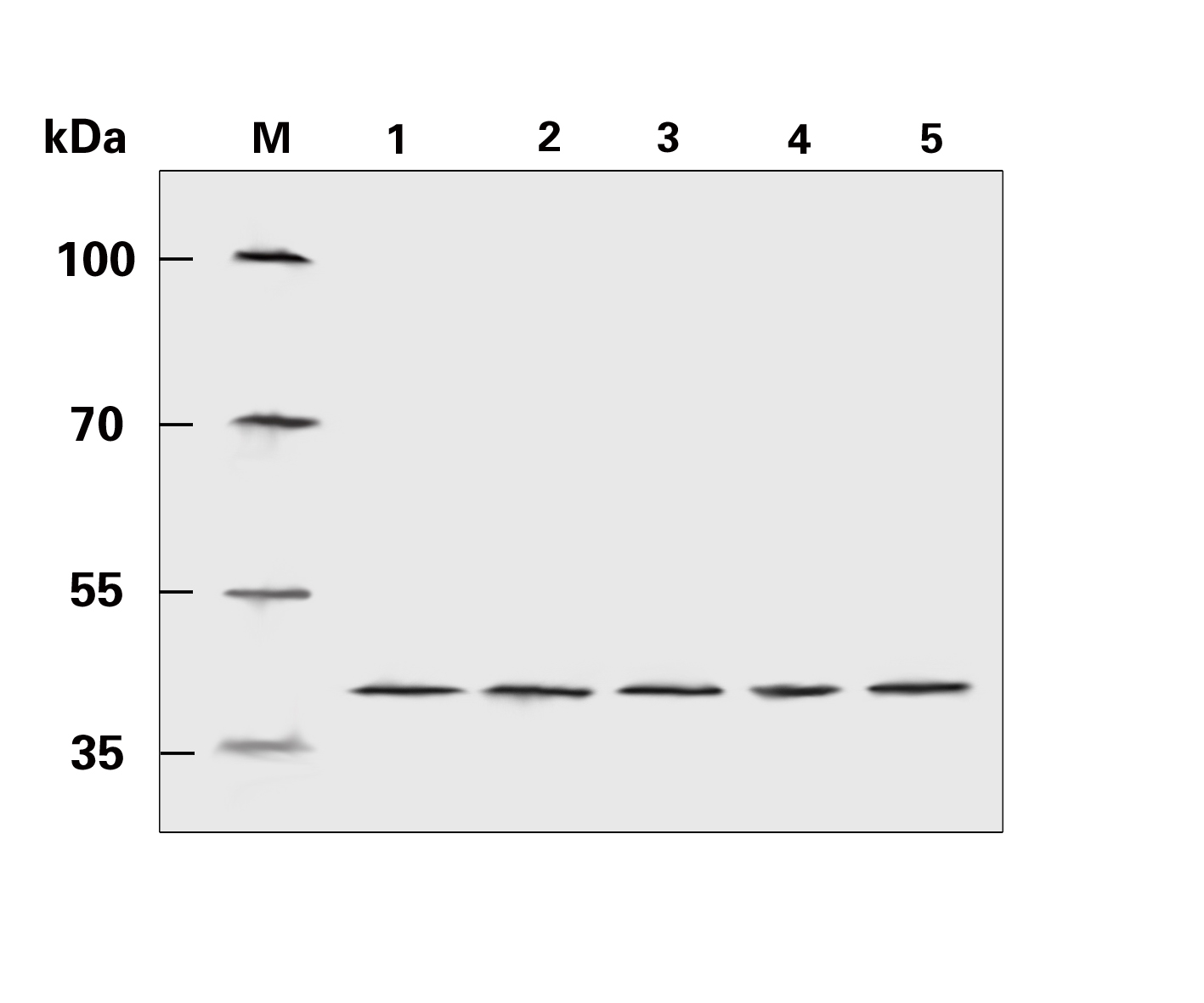


M: marker protein

1: control group

2: Dex group

3: Res group

4: Dex+Res group

5: Res+NAM group

β-actin=43kDa

Figure 6


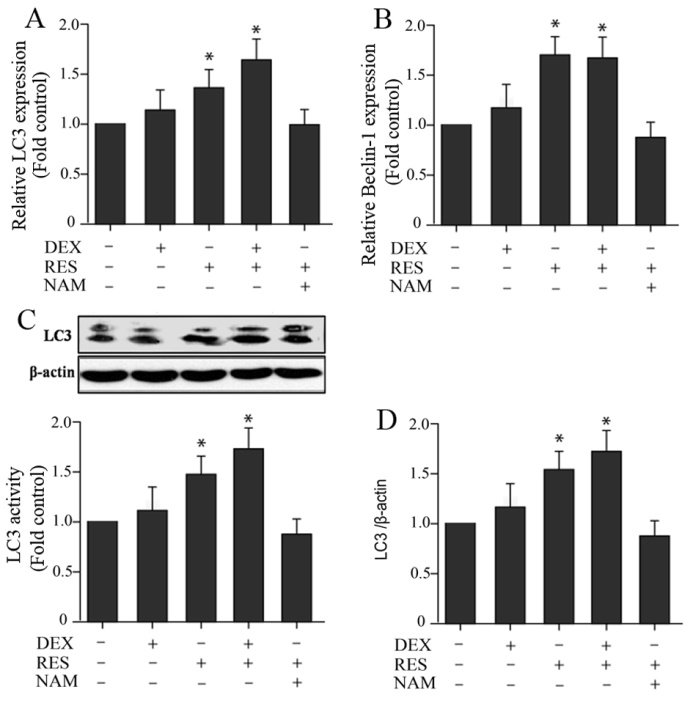


Figure 6C1


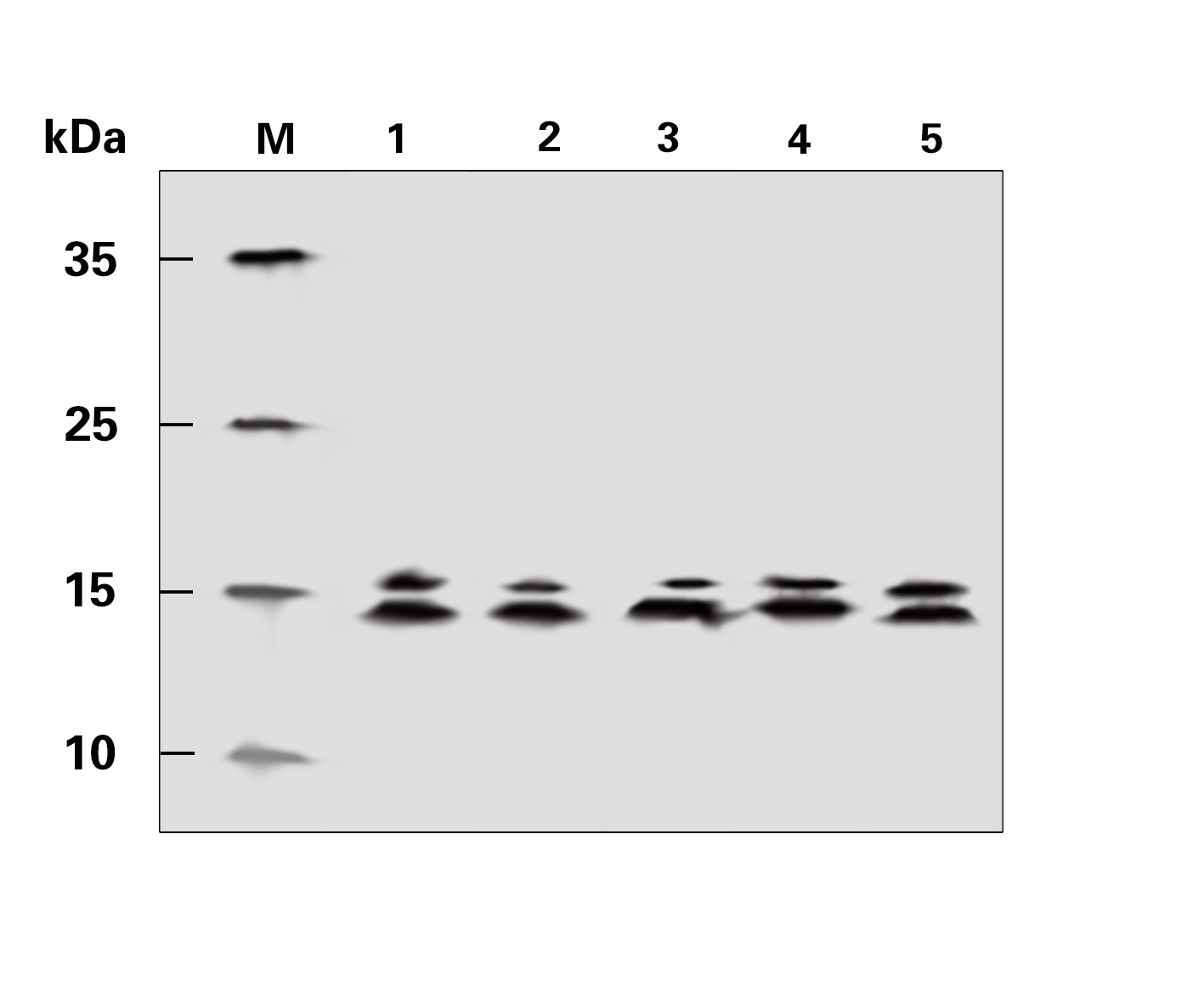


M：marker protein

1：control group

2：Dex group

3: Res group

4: Dex+Res group

5: Res+NAM group

LC3-I=16kDa, LC3-II=14kDa

Figure 6C2


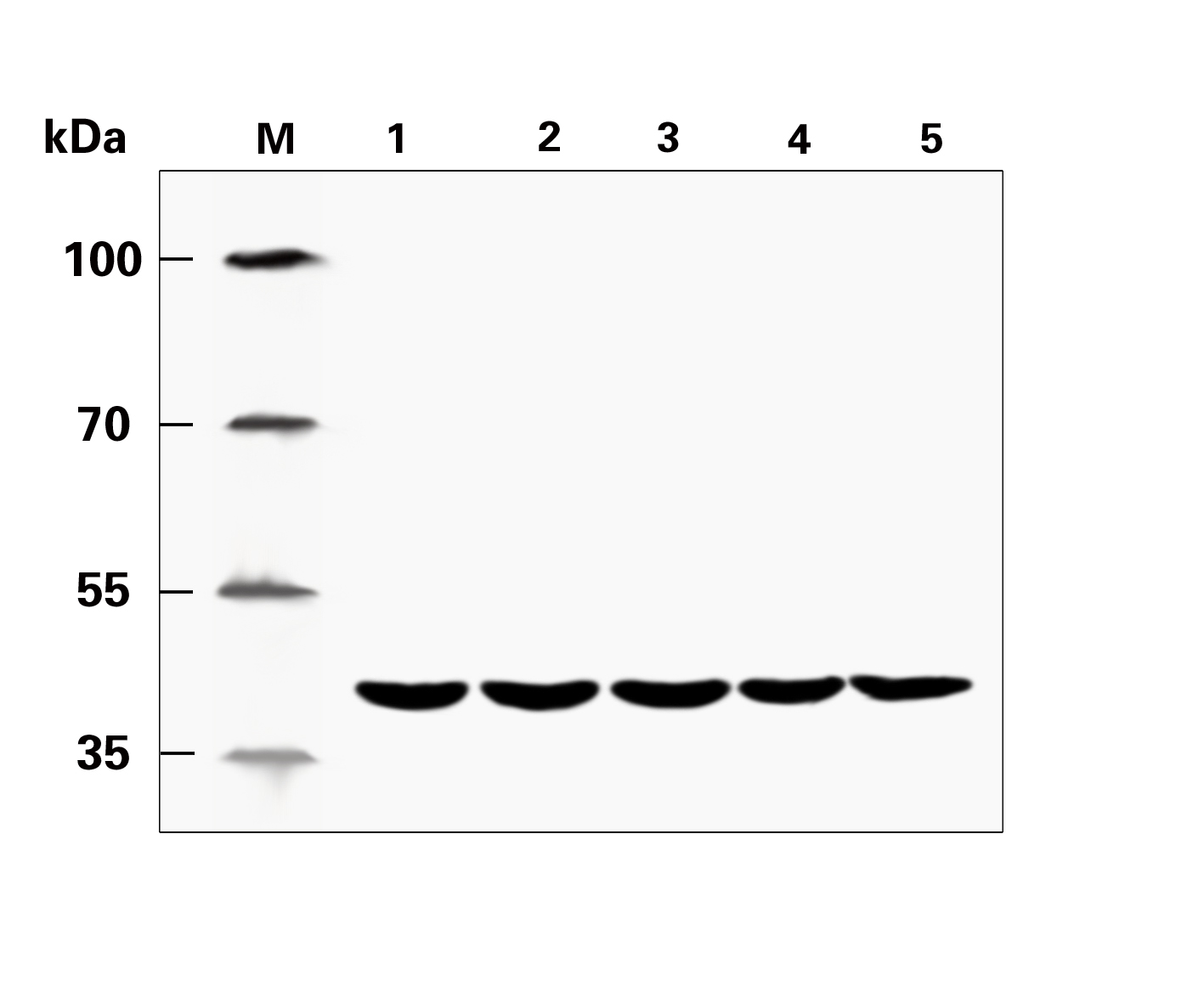


M：marker protein

1：control group

2：Dex group

3: Res group

4: Dex+Res group

5: Res+NAM group

β-actin=43kDa

Figure 8


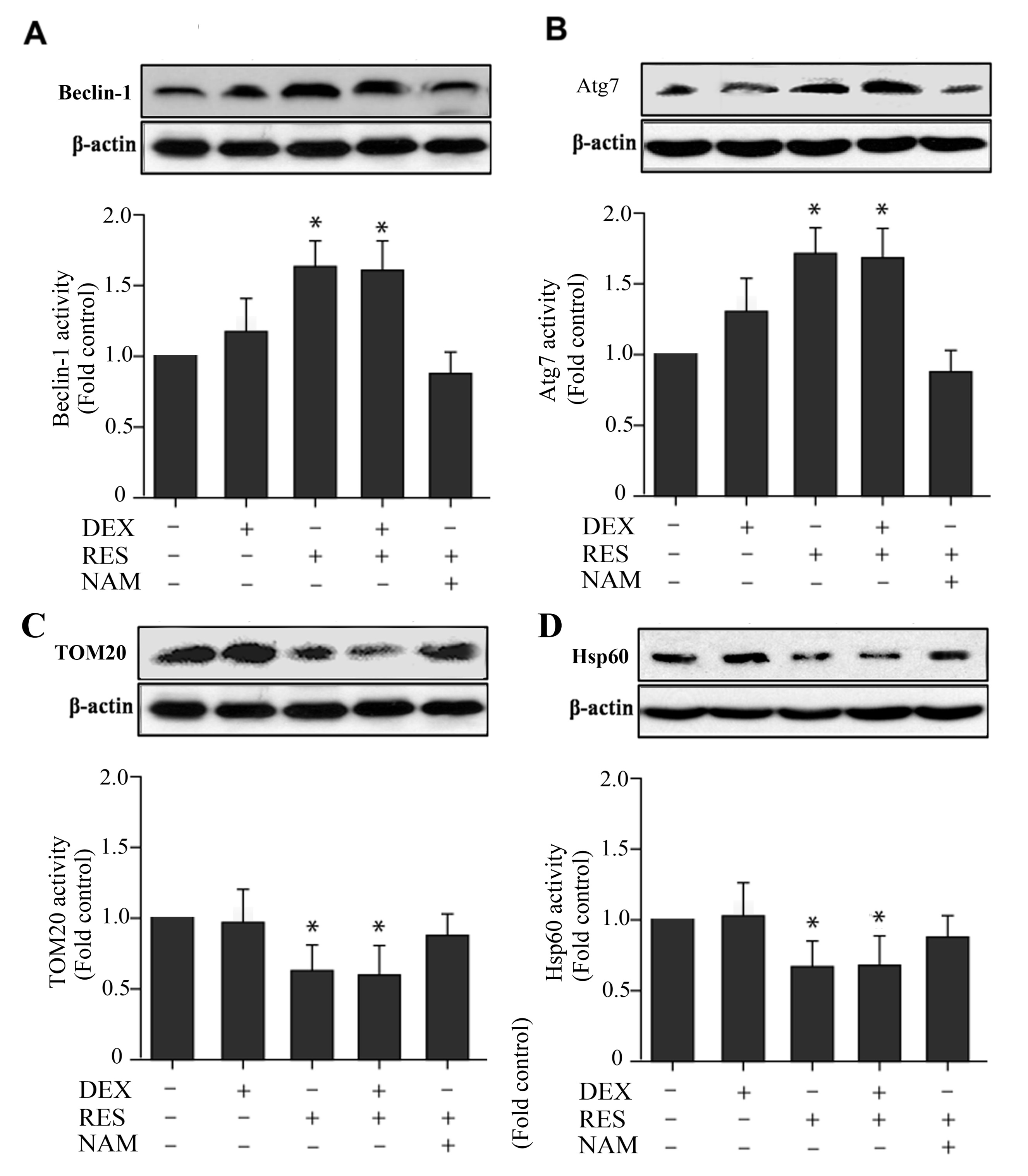


Figure 8A1


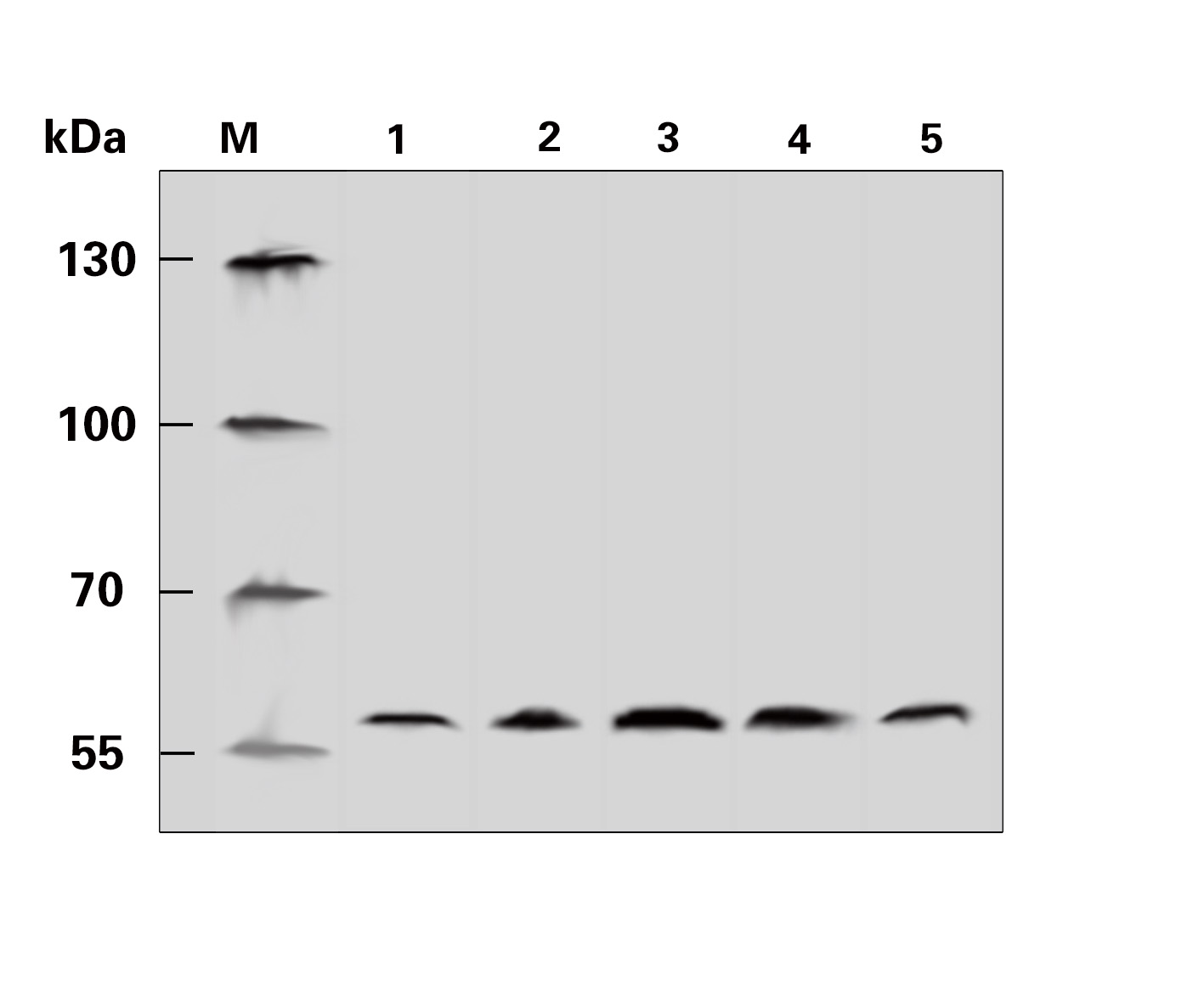


M：marker protein

1：control group

2：Dex group

3: Res group

4: Dex+Res group

5: Res+NAM group

Beclin-1=60kDa

Figure 8A2


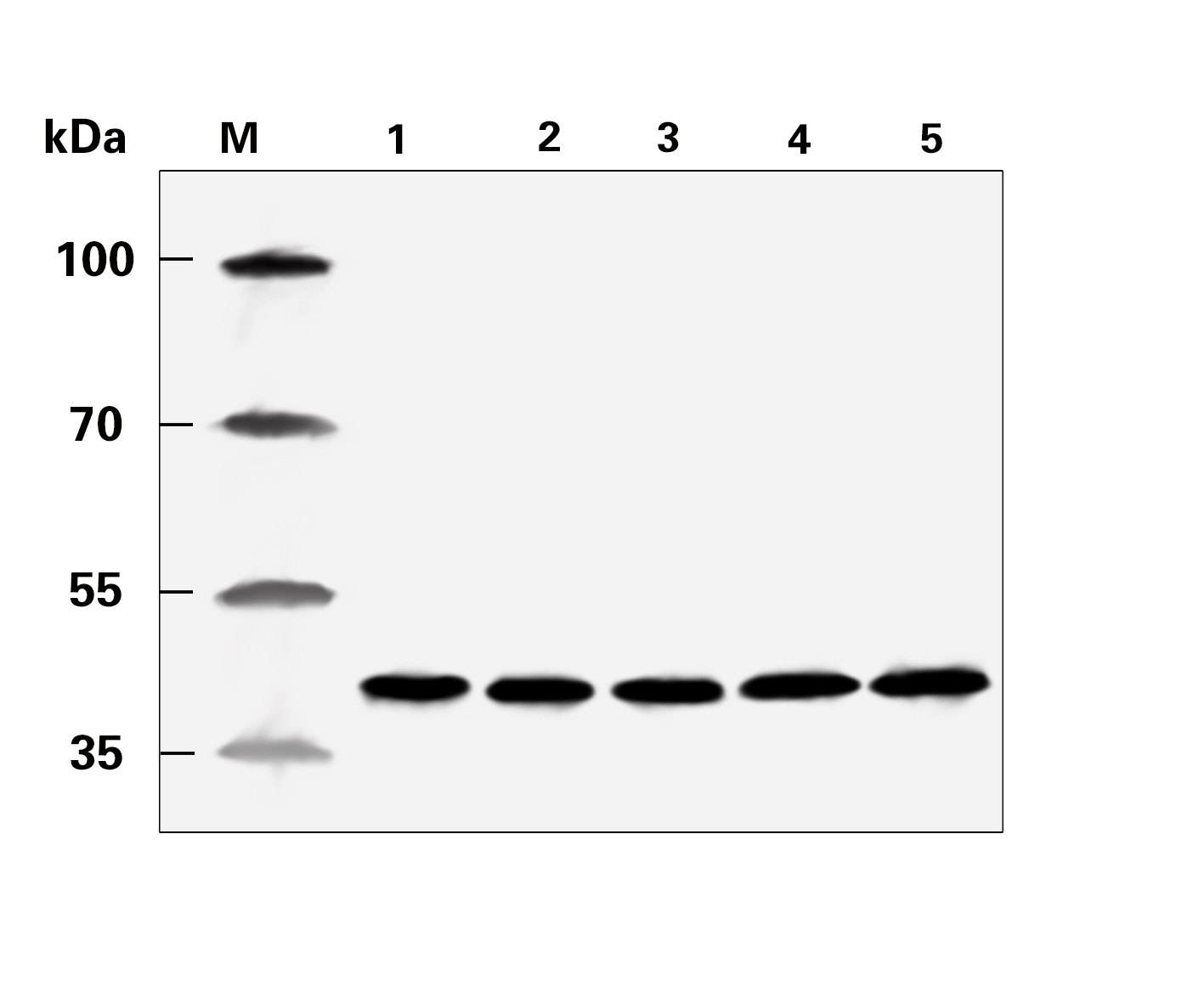


M：marker protein

1：control group

2：Dex group

3: Res group

4: Dex+Res group

5: Res+NAM group

β-actin=43kDa

Figure 8B1


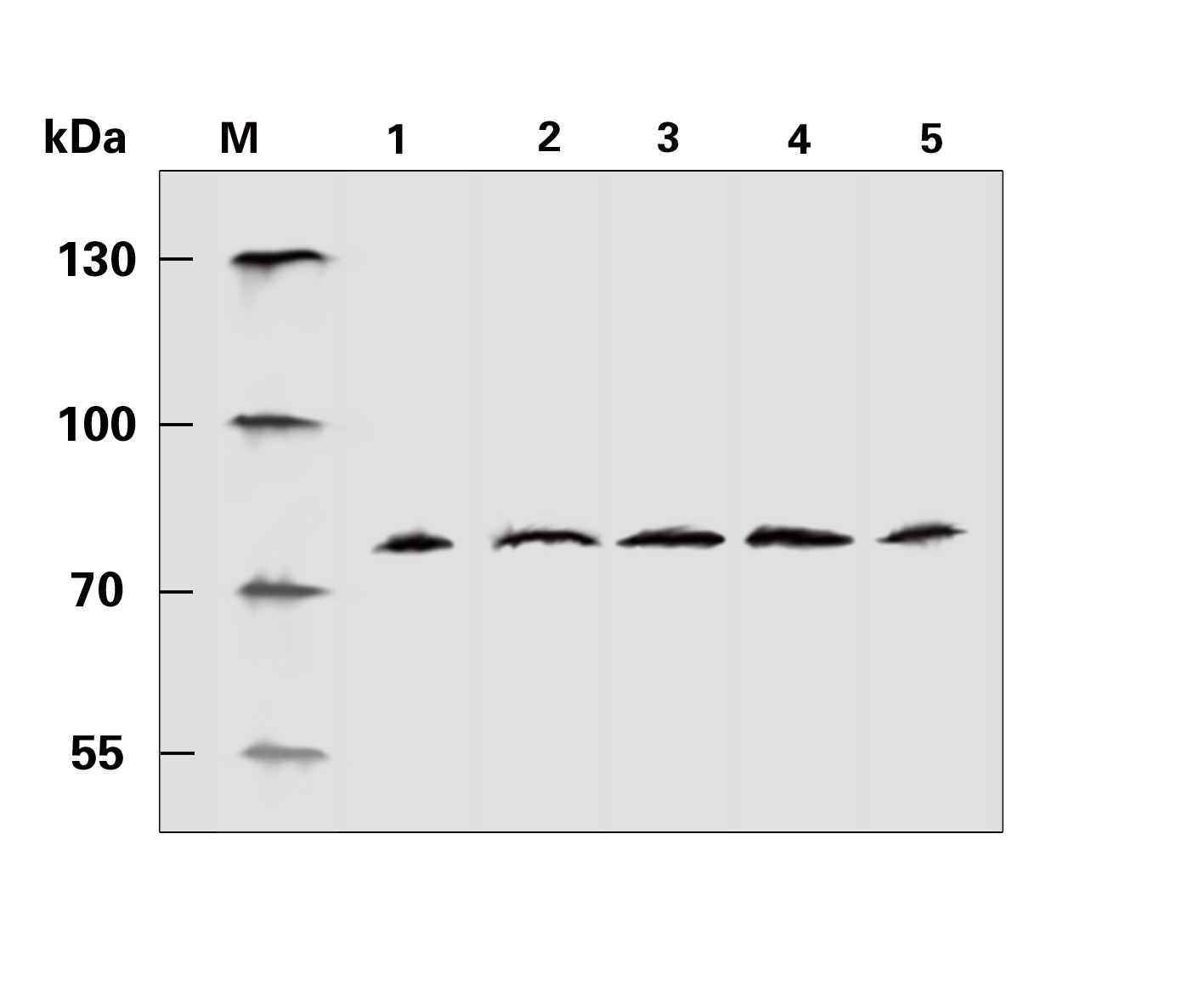


M：marker protein

1：control group

2：Dex group

3: Res group

4: Dex+Res group

5: Res+NAM group

Atg7=78kDa

Figure 8B2


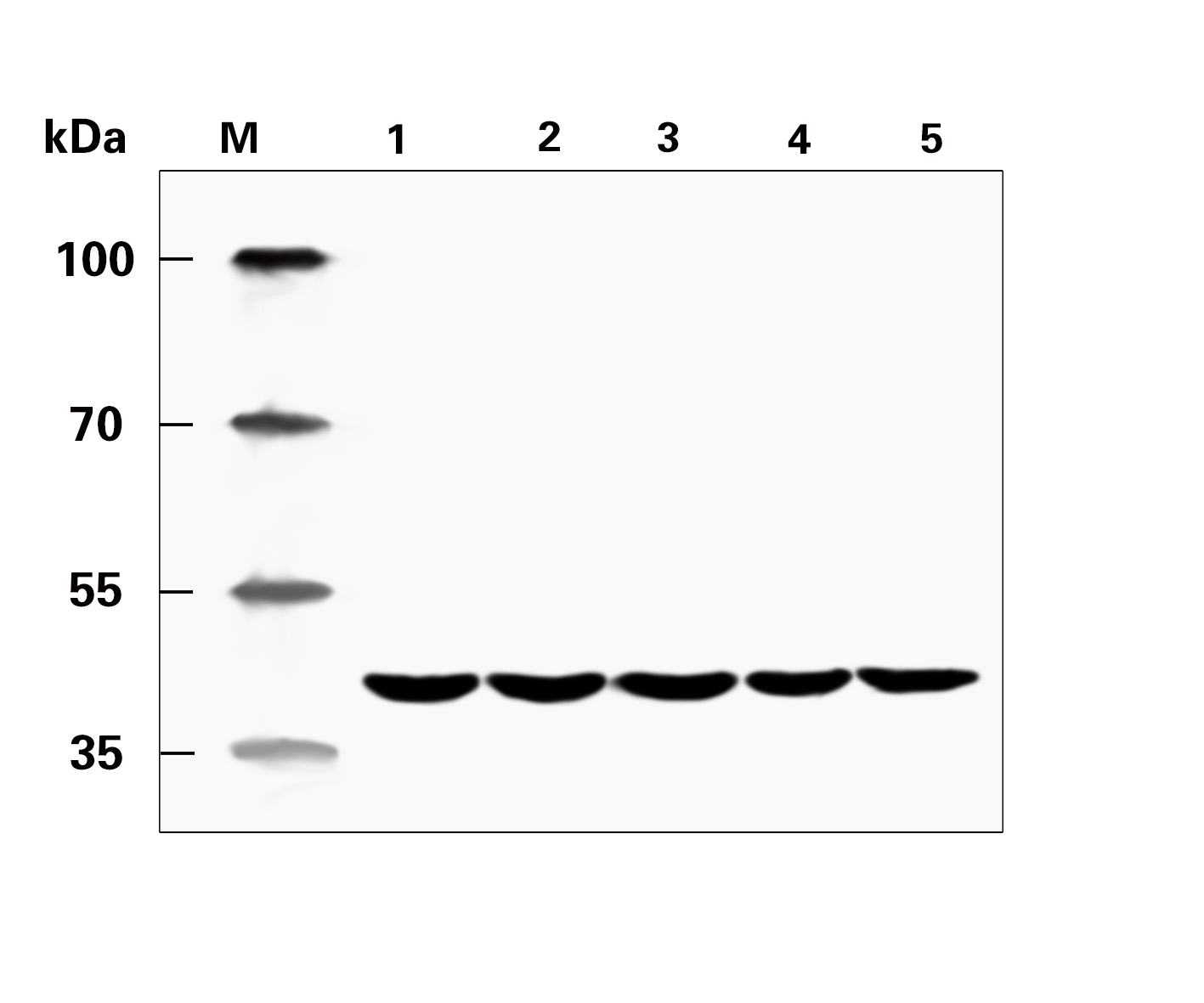


M：marker protein

1：control group

2：Dex group

3: Res group

4: Dex+Res group

5: Res+NAM group

β-actin=43kDa

Figure 8C1


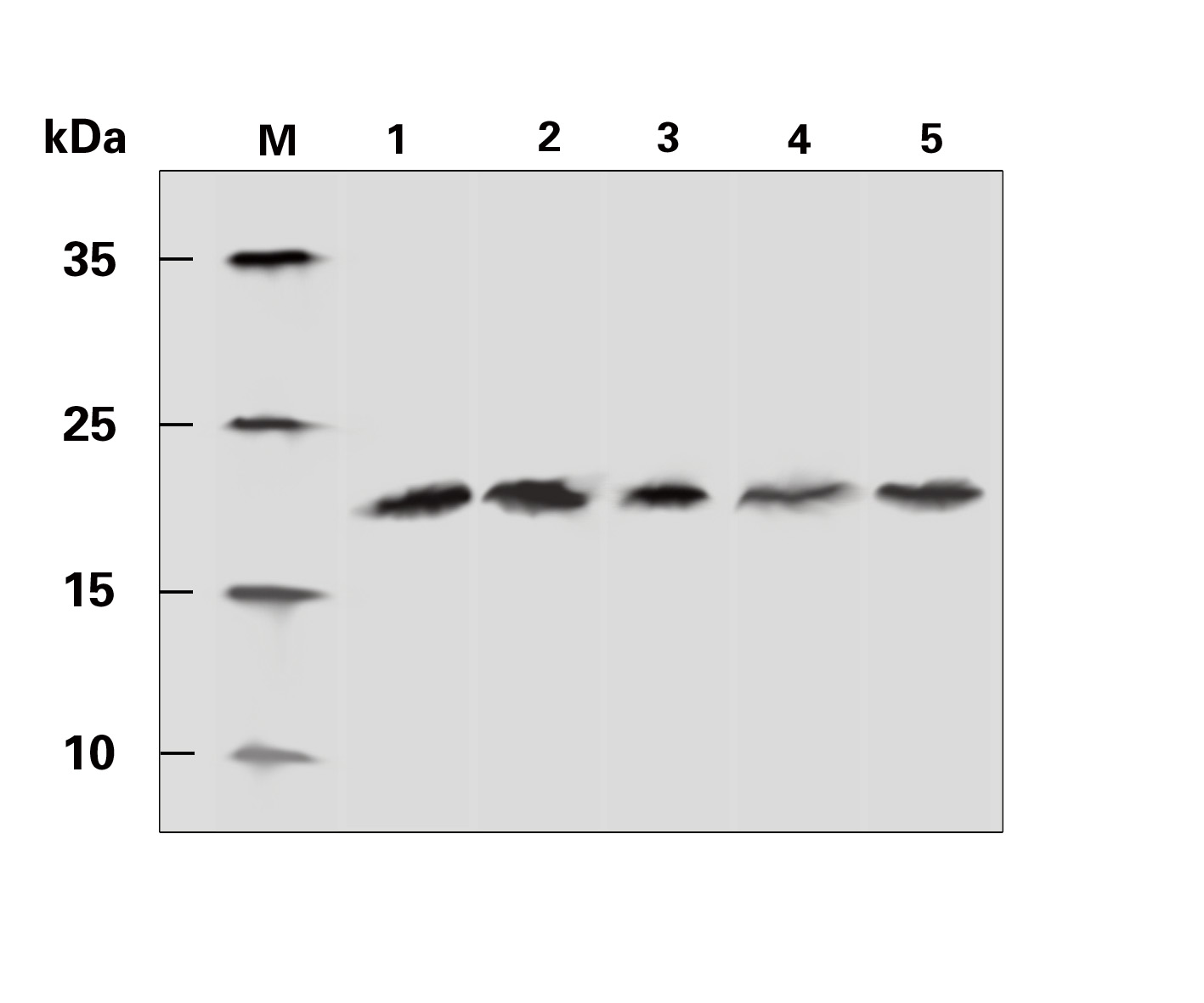


M：marker protein

1：control group

2：Dex group

3: Res group

4: Dex+Res group

5: Res+NAM group

TOM20=20kDa

Figure 8C2


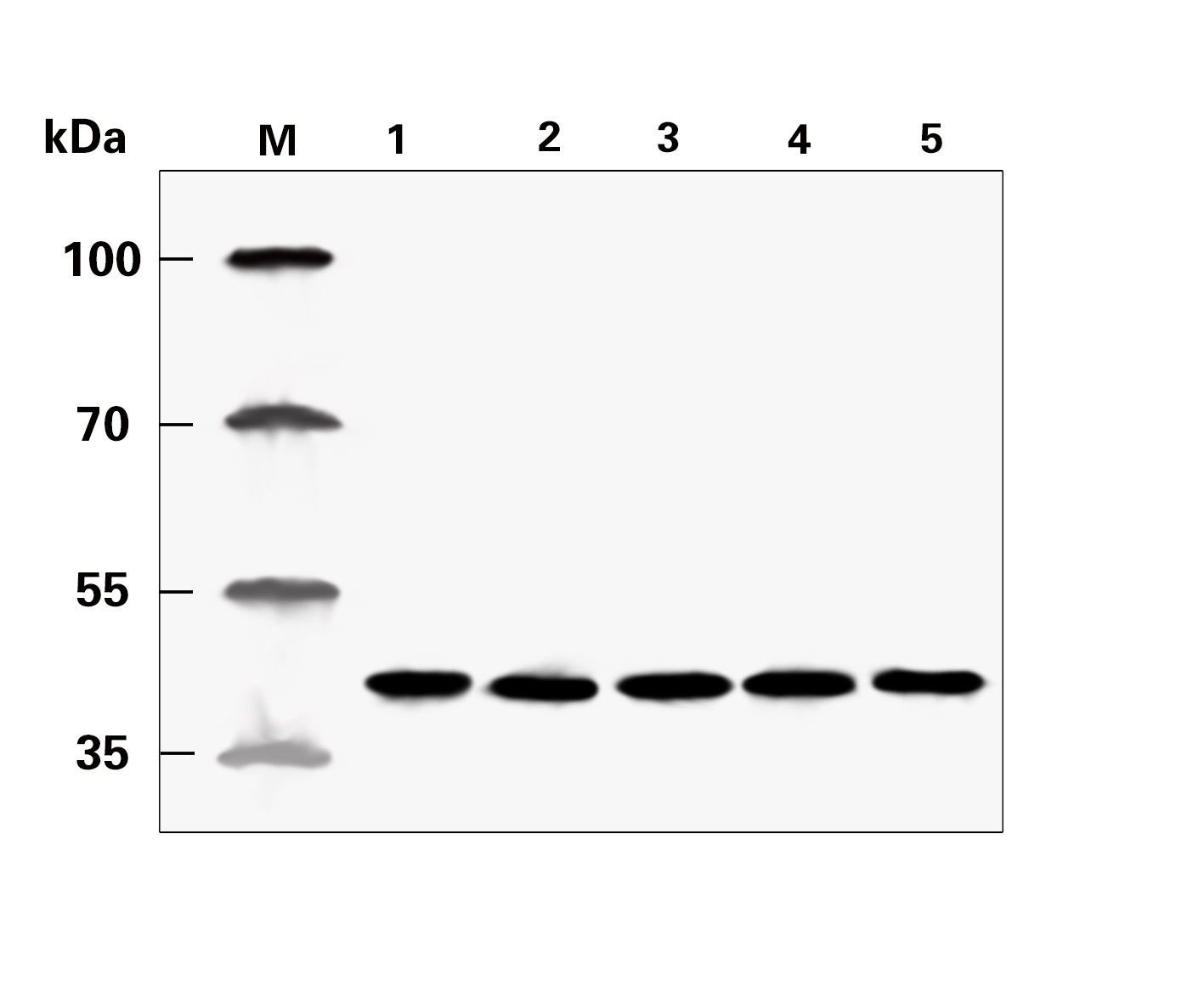


M：marker protein

1：control group

2：Dex group

3: Res group

4: Dex+Res group

5: Res+NAM group

β-actin=43kDa

Figure 8D1


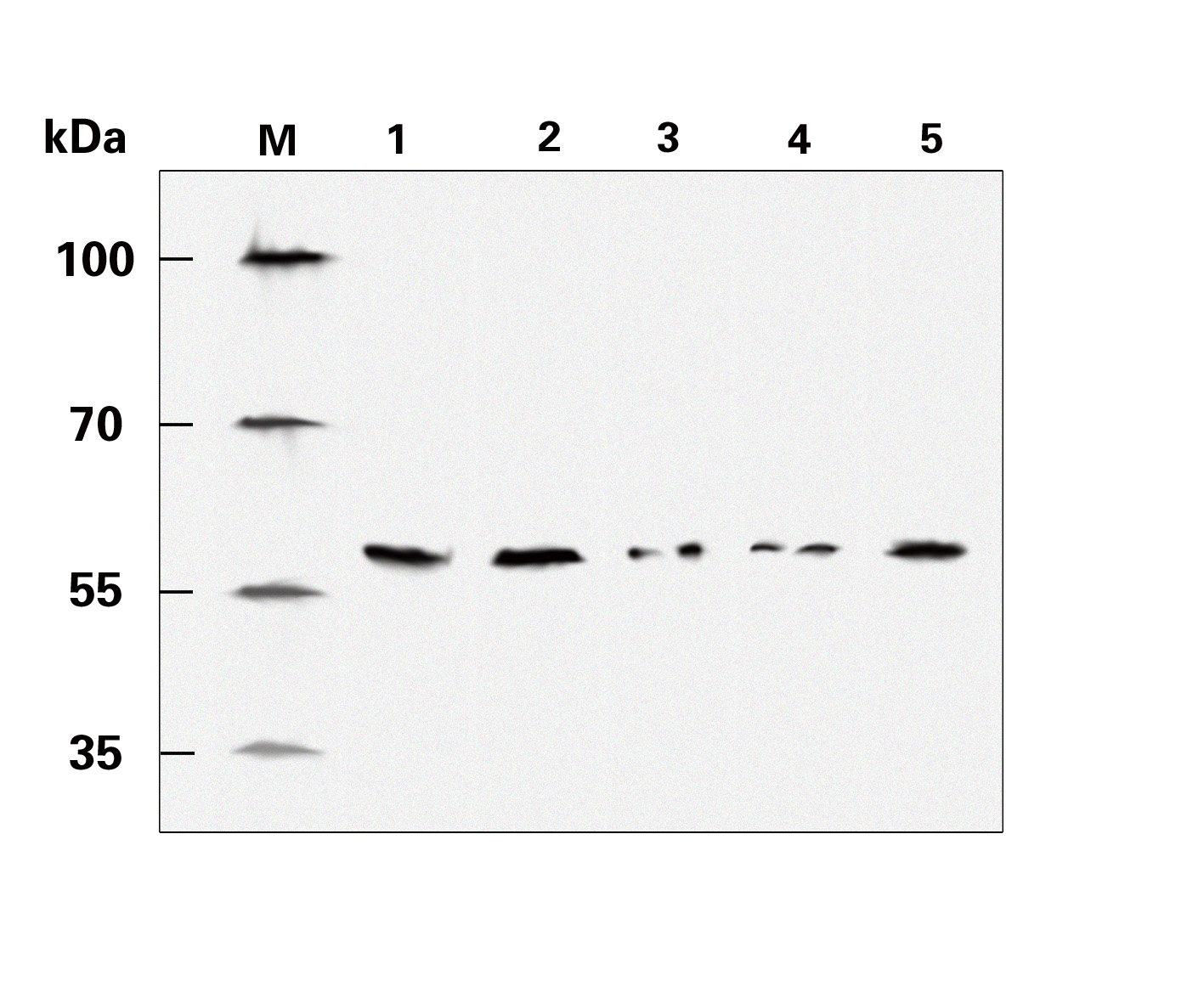


M：marker protein

1：control group

2：Dex group

3: Res group

4: Dex+Res group

5: Res+NAM group

Hsp60=60kDa

Figure 8D2


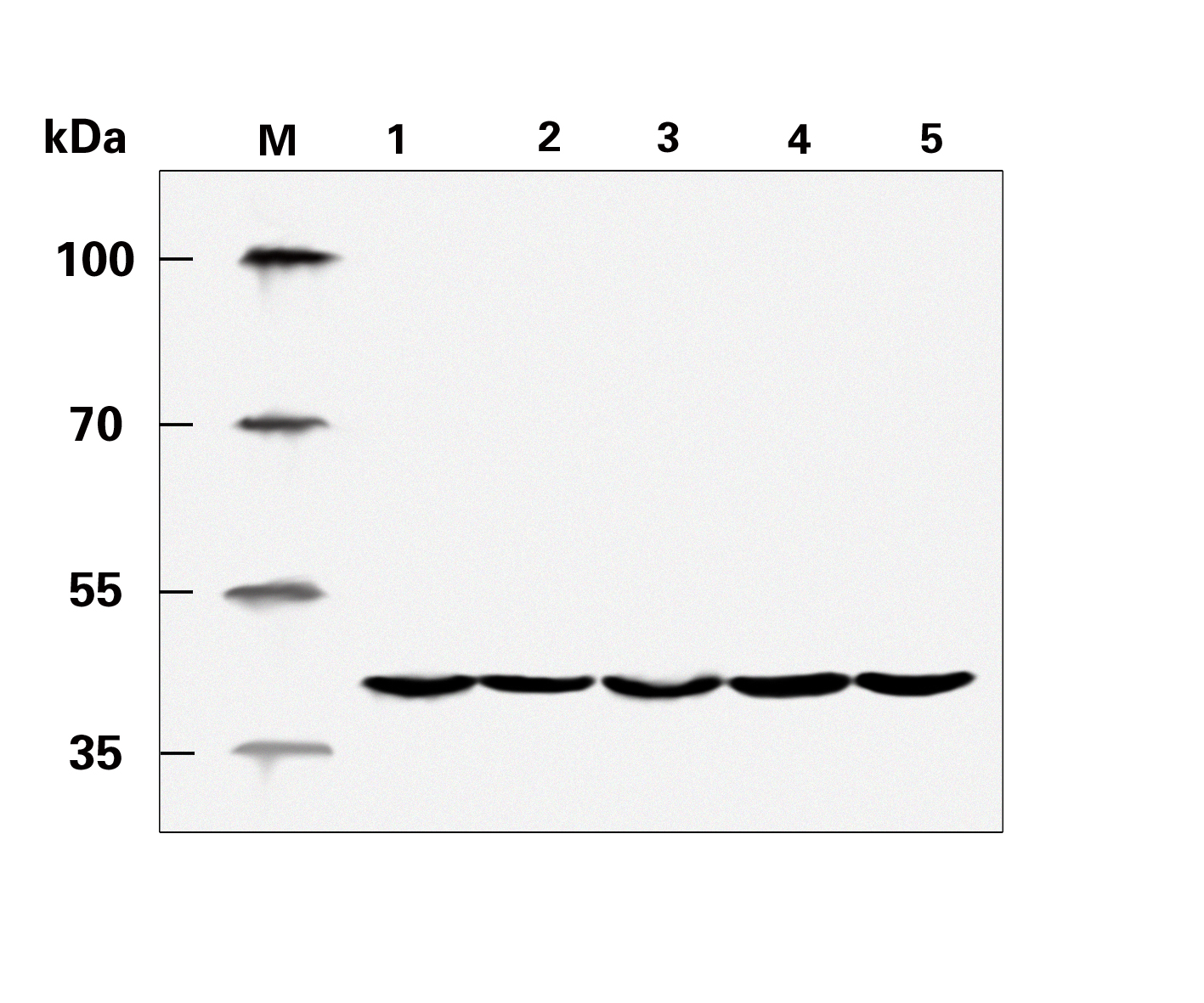


M：marker protein

1：control group

2：Dex group

3: Res group

4: Dex+Res group

5: Res+NAM group

β-actin=43kDa

Figure 9


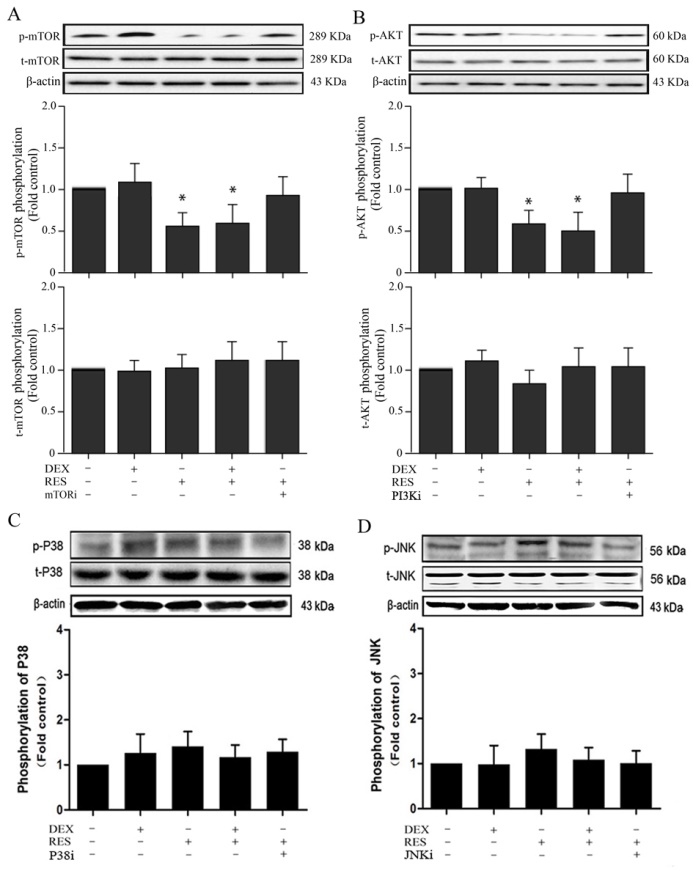


Figure 9A1


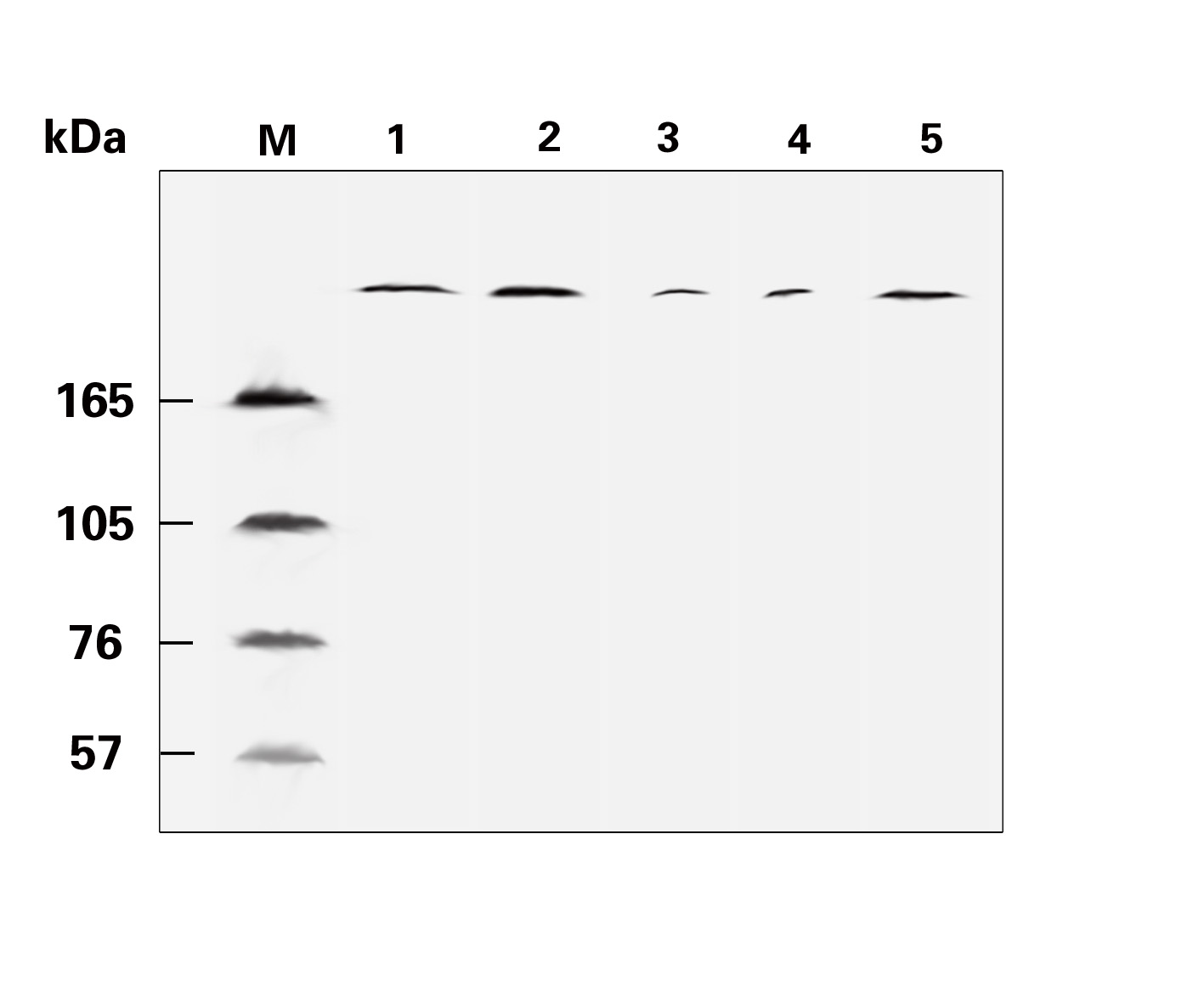


M：marker protein

1：control group

2：Dex group

3: Res group

4: Dex+Res group

5: Res+mTORi group

*p*-m-TOR=289kDa

Figure 9A2


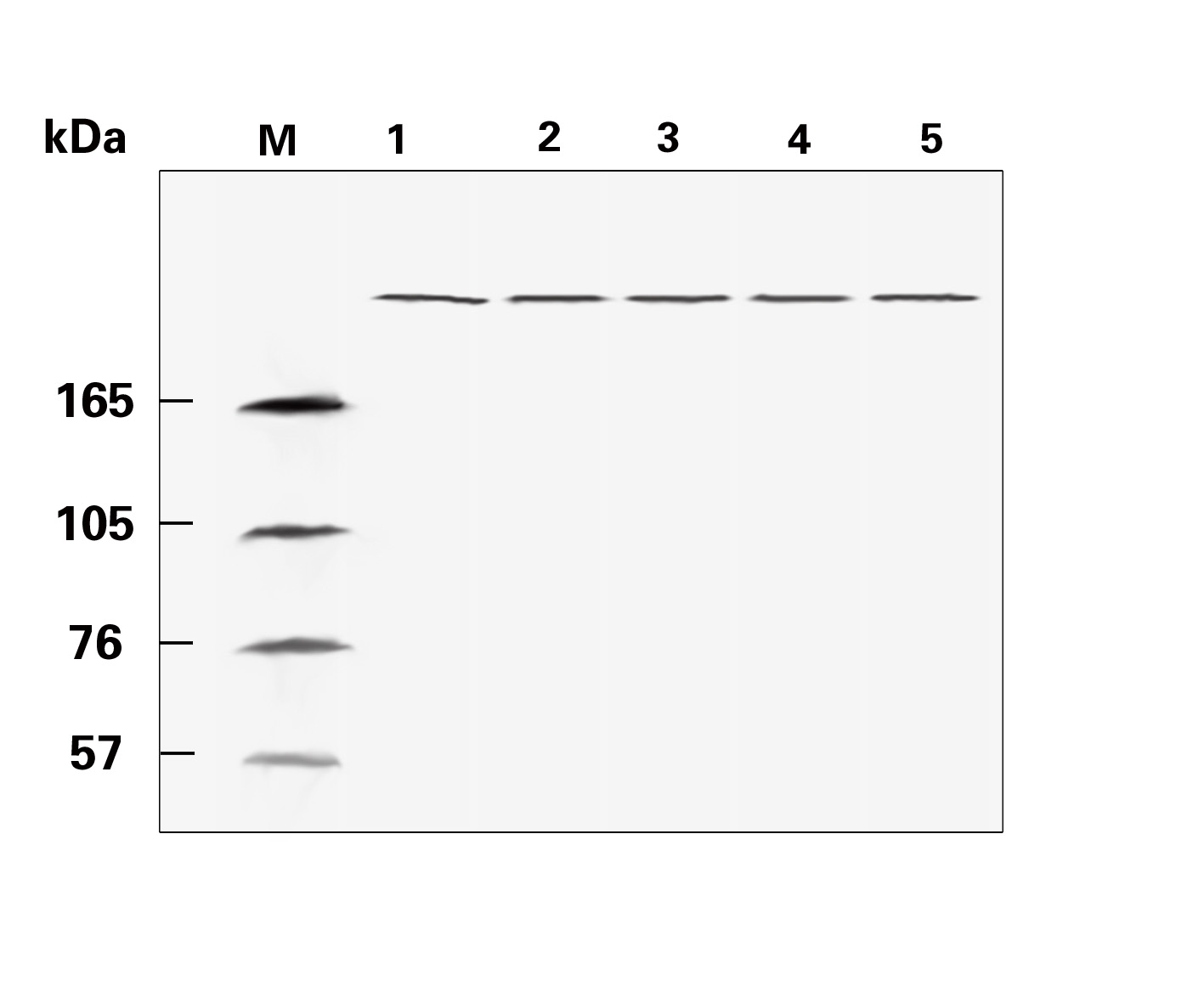


M：marker protein

1：control group

2：Dex group

3: Res group

4: Dex+Res group

5: Res+mTORi group

*t*-m-TOR=289kDa

Figure 9A3


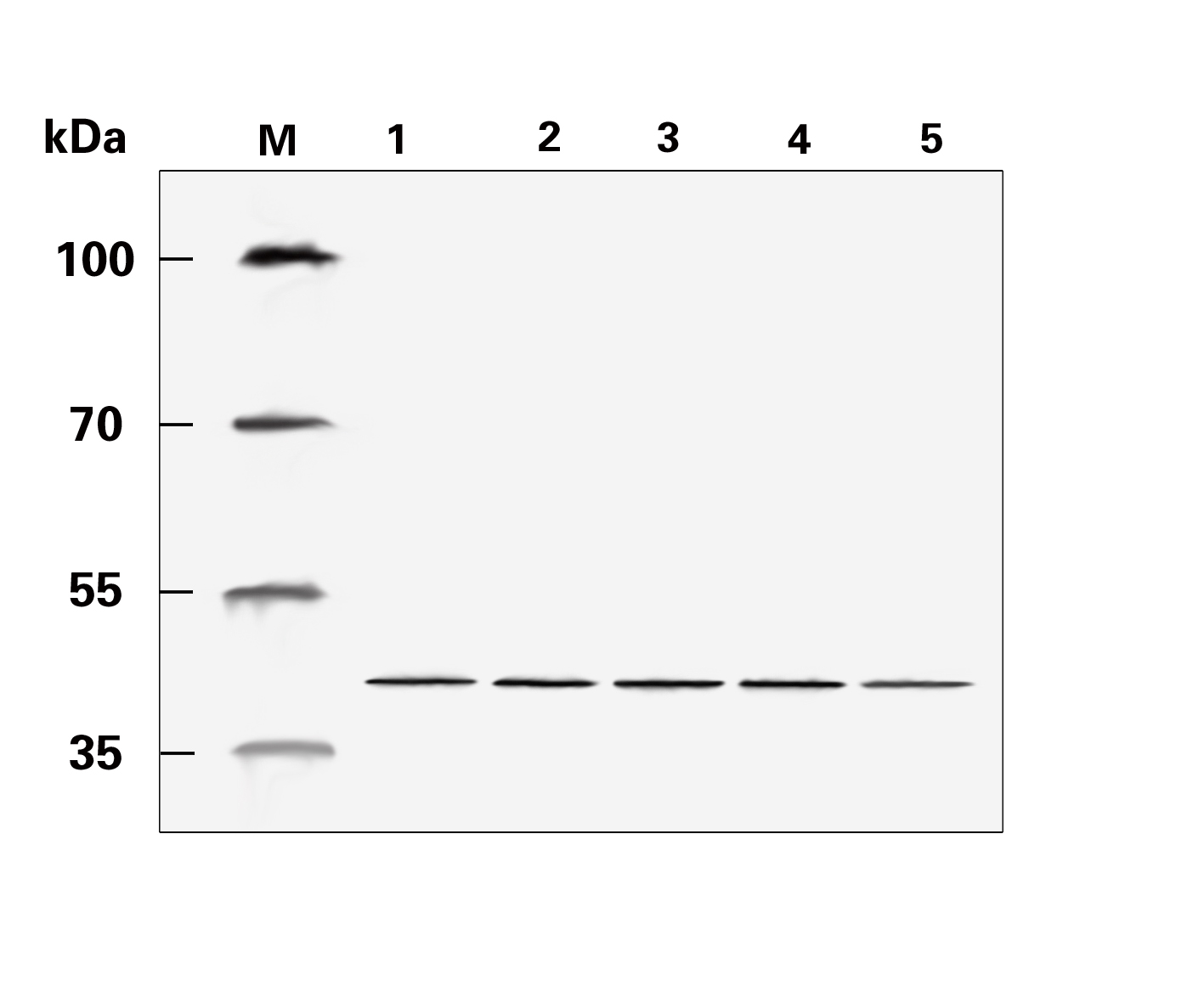


M：marker protein

1：control group

2：Dex group

3: Res group

4: Dex+Res group

5: Res+mTORi group

β-actin=43kDa

Figure 9B1


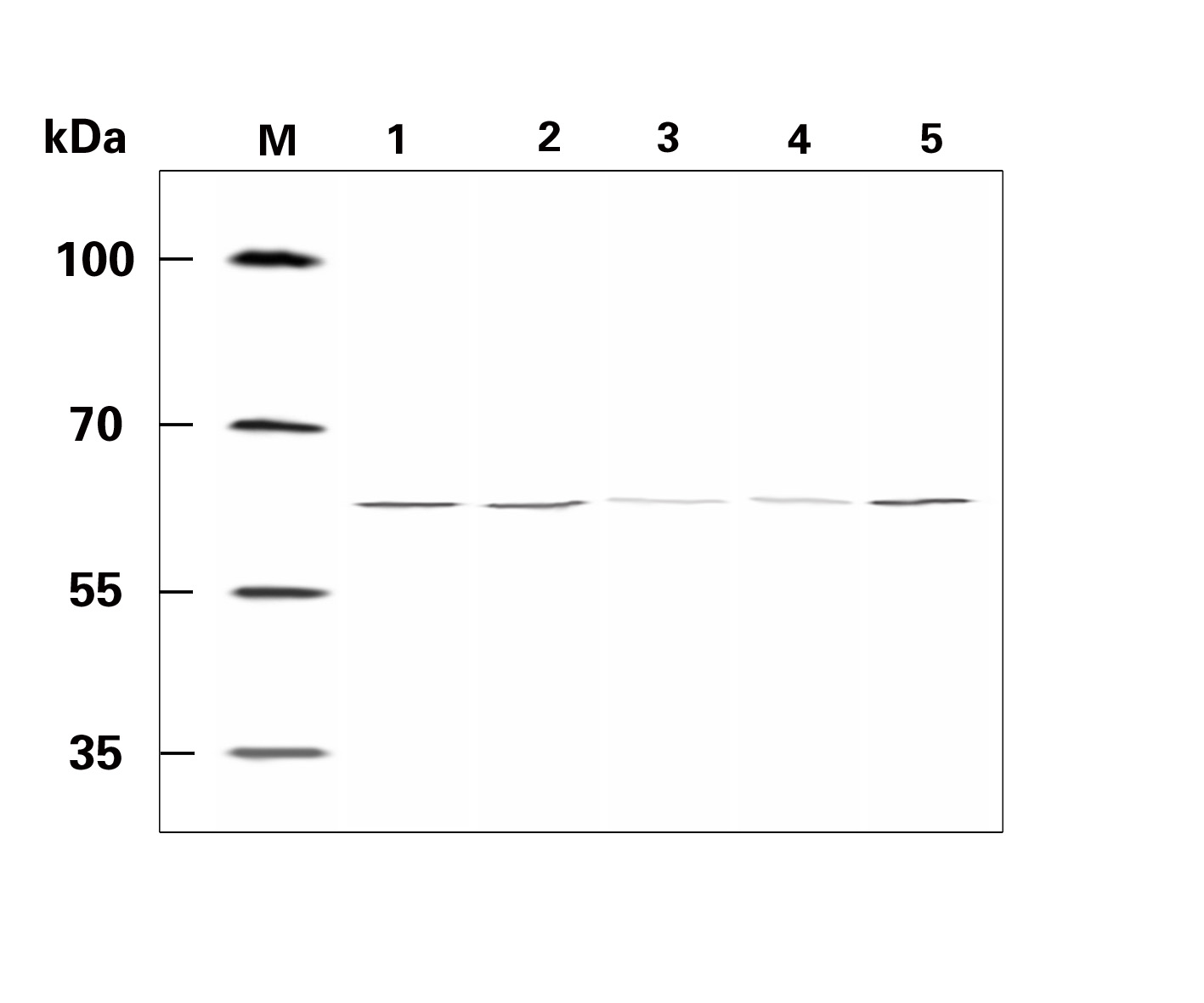


M：marker protein

1：control group

2：Dex group

3: Res group

4: Dex+Res group

5: Res+ PI3Ki group

*p*-Akt=60kDa

Figure 9B2


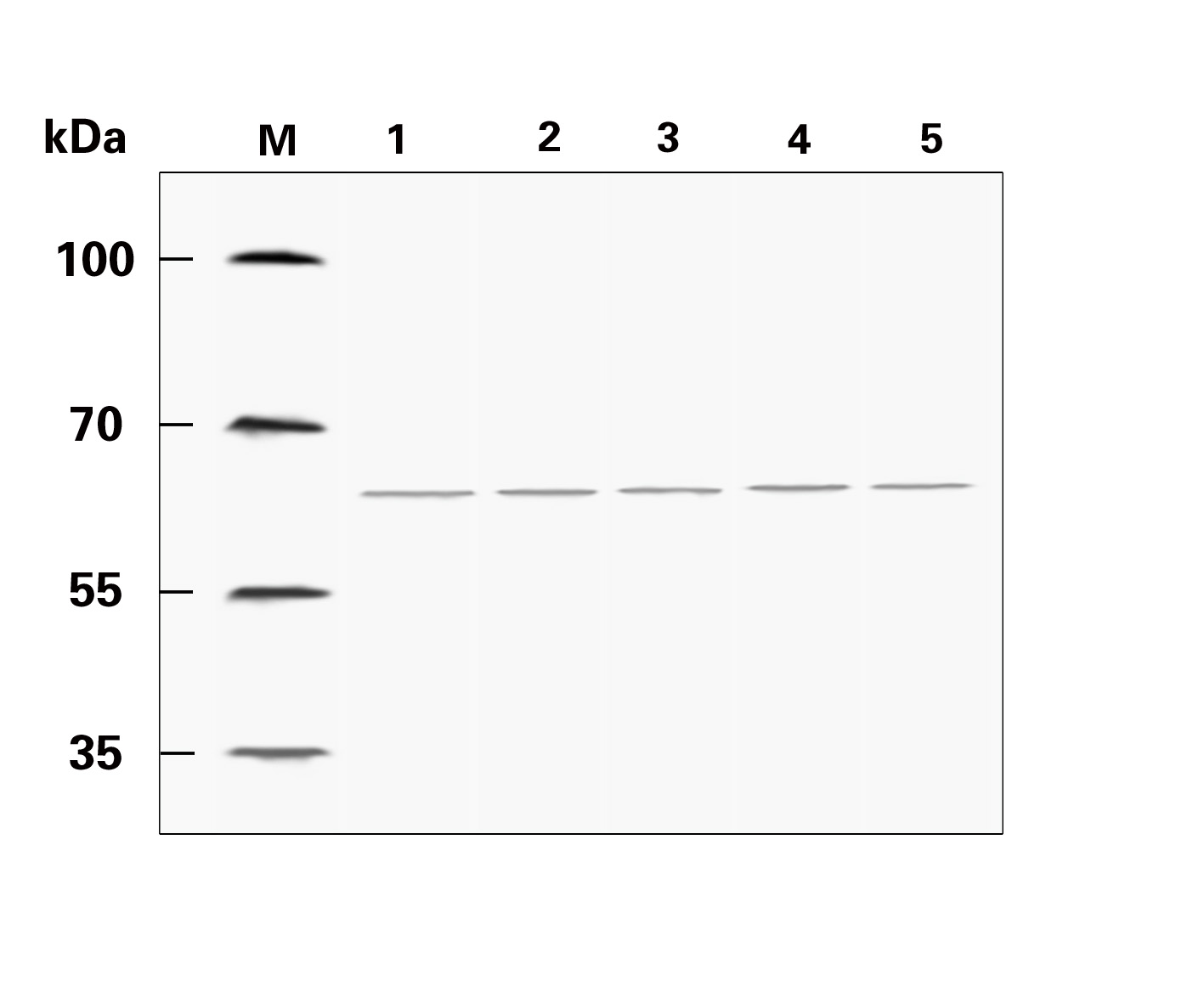


M：marker protein

1：control group

2：Dex group

3: Res group

4: Dex+Res group

5: Res+ PI3Ki group

*t*-Akt=60kDa

Figure 9B3


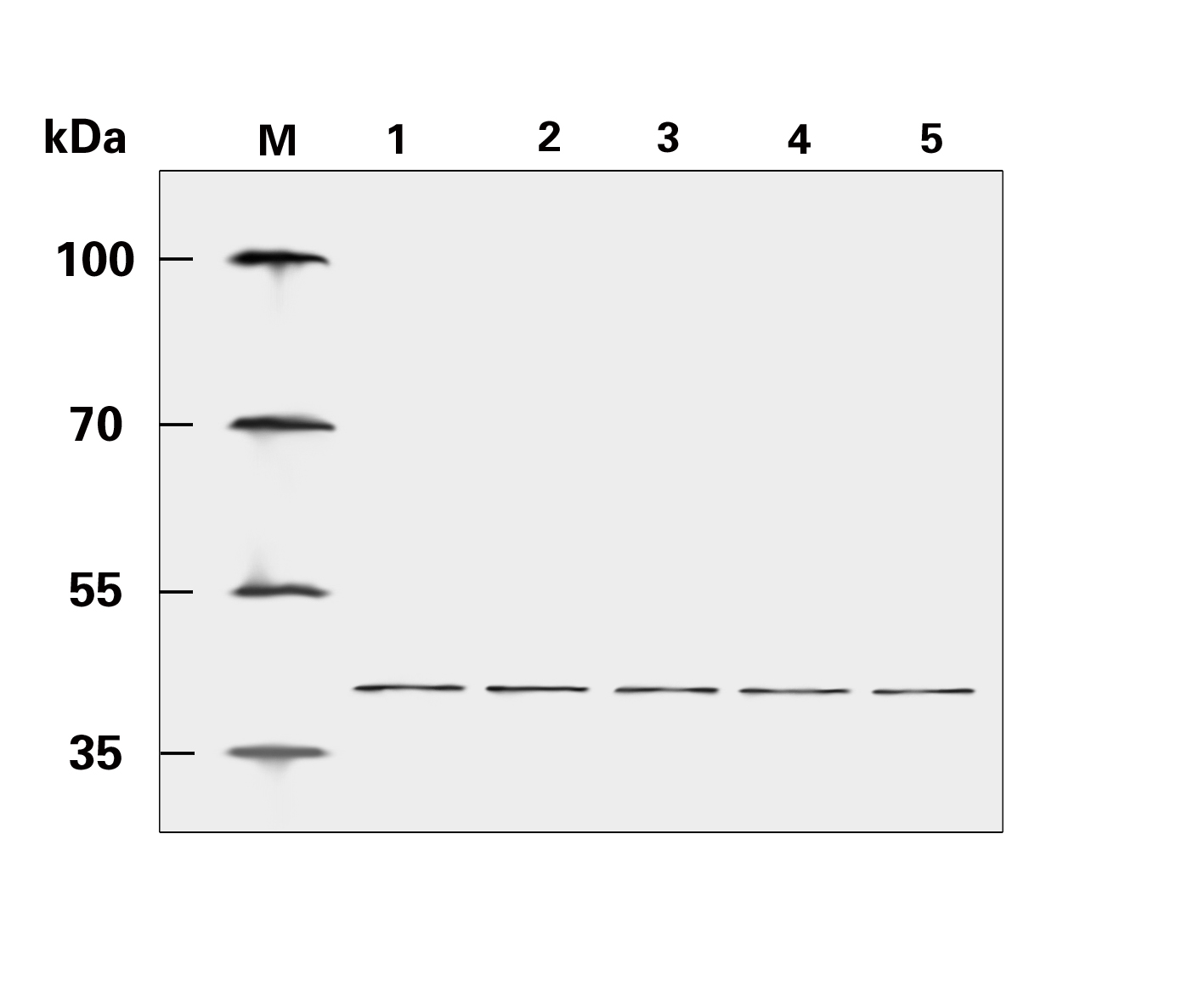


M：marker protein

1：control group

2：Dex group

3: Res group

4: Dex+Res group

5: Res+ PI3Ki group

β-actin=43kDa

Figure 9C1


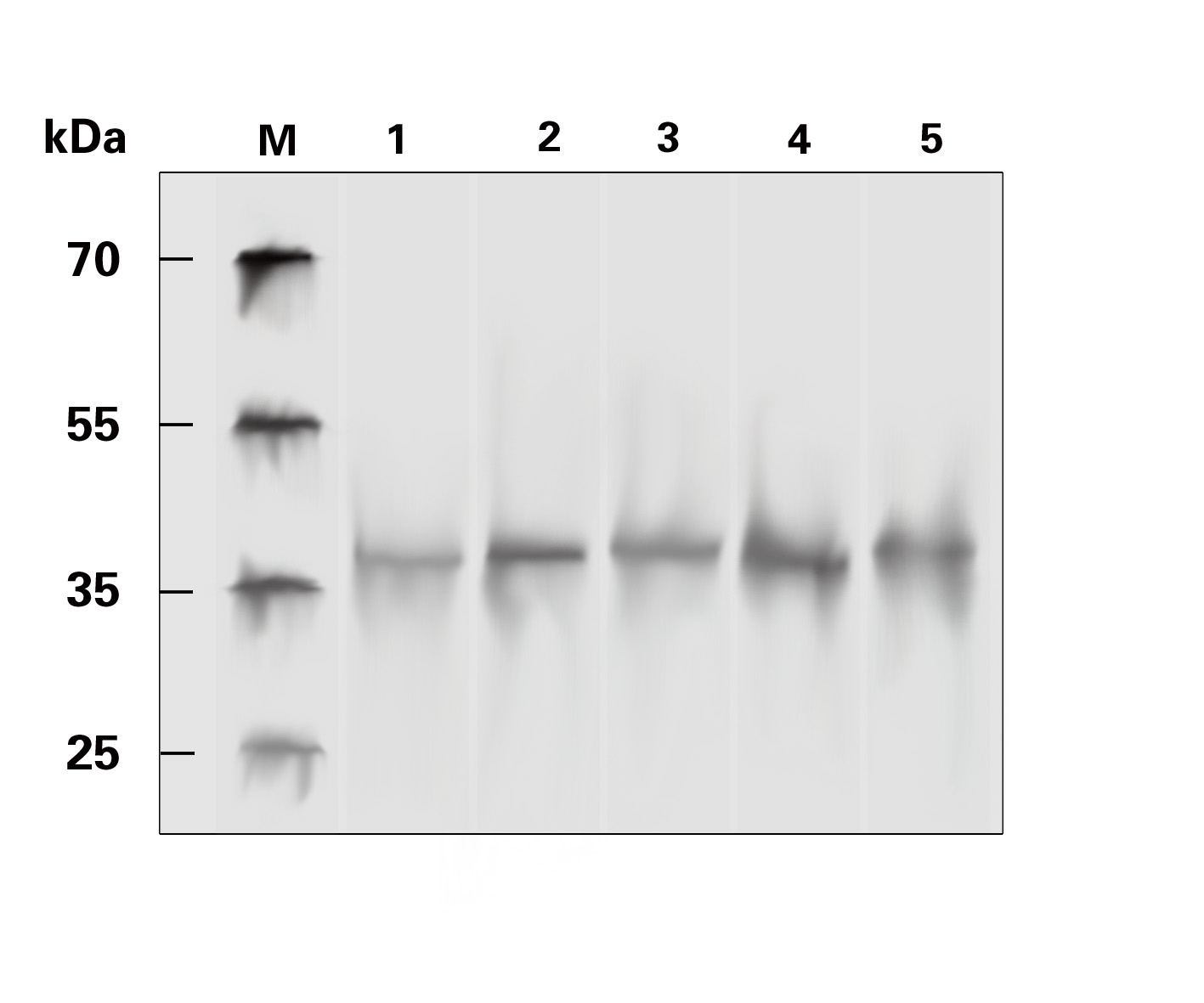


M：marker protein

1：control group

2：Dex group

3: Res group

4: Dex+Res group

5: Res+ P38i group

*p*-P38=38kDa

Figure 9C2


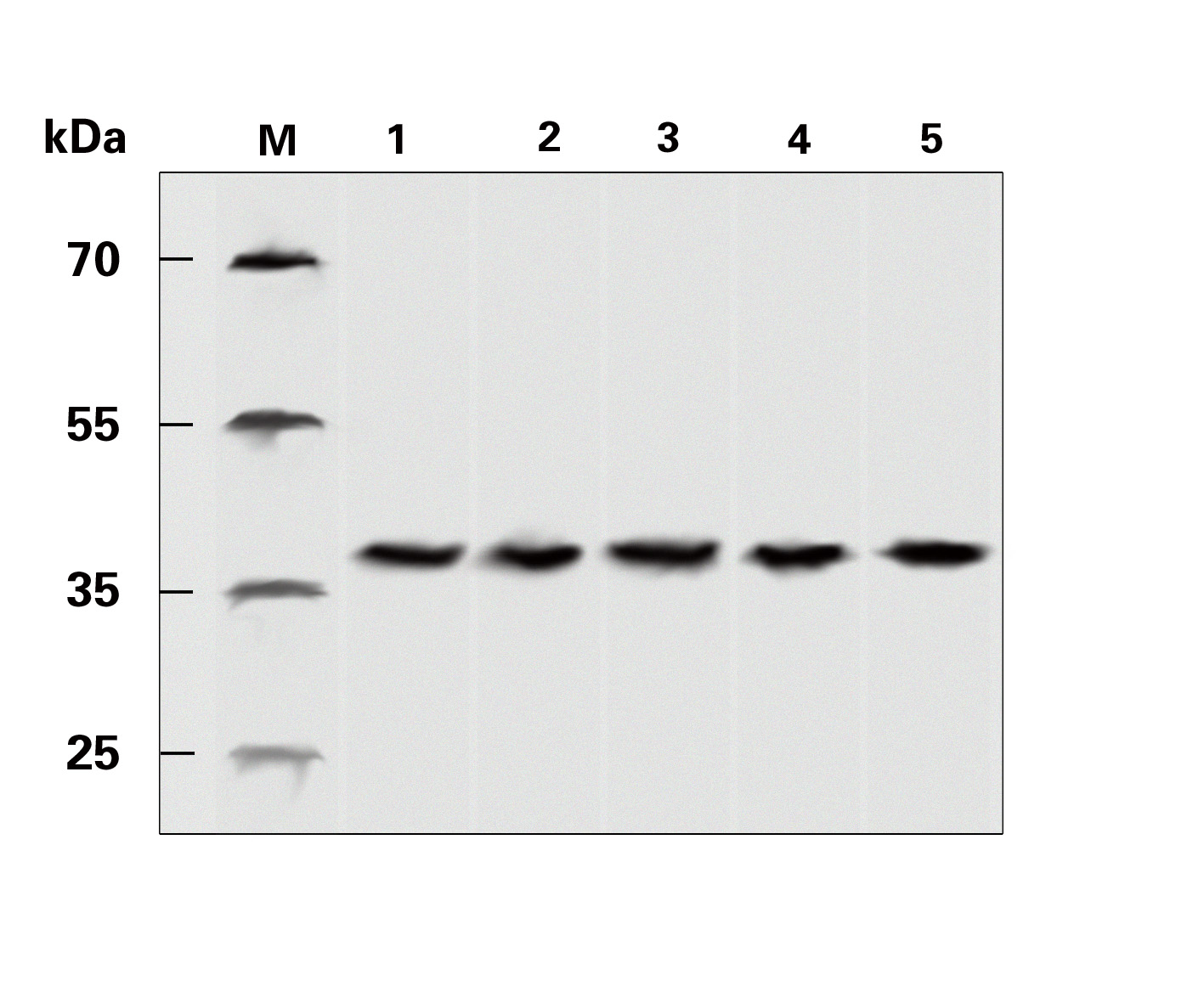


M：marker protein

1：control group

2：Dex group

3: Res group

4: Dex+Res group

5: Res+ P38i group

*t*-P38=38kDa

Figure 9C3


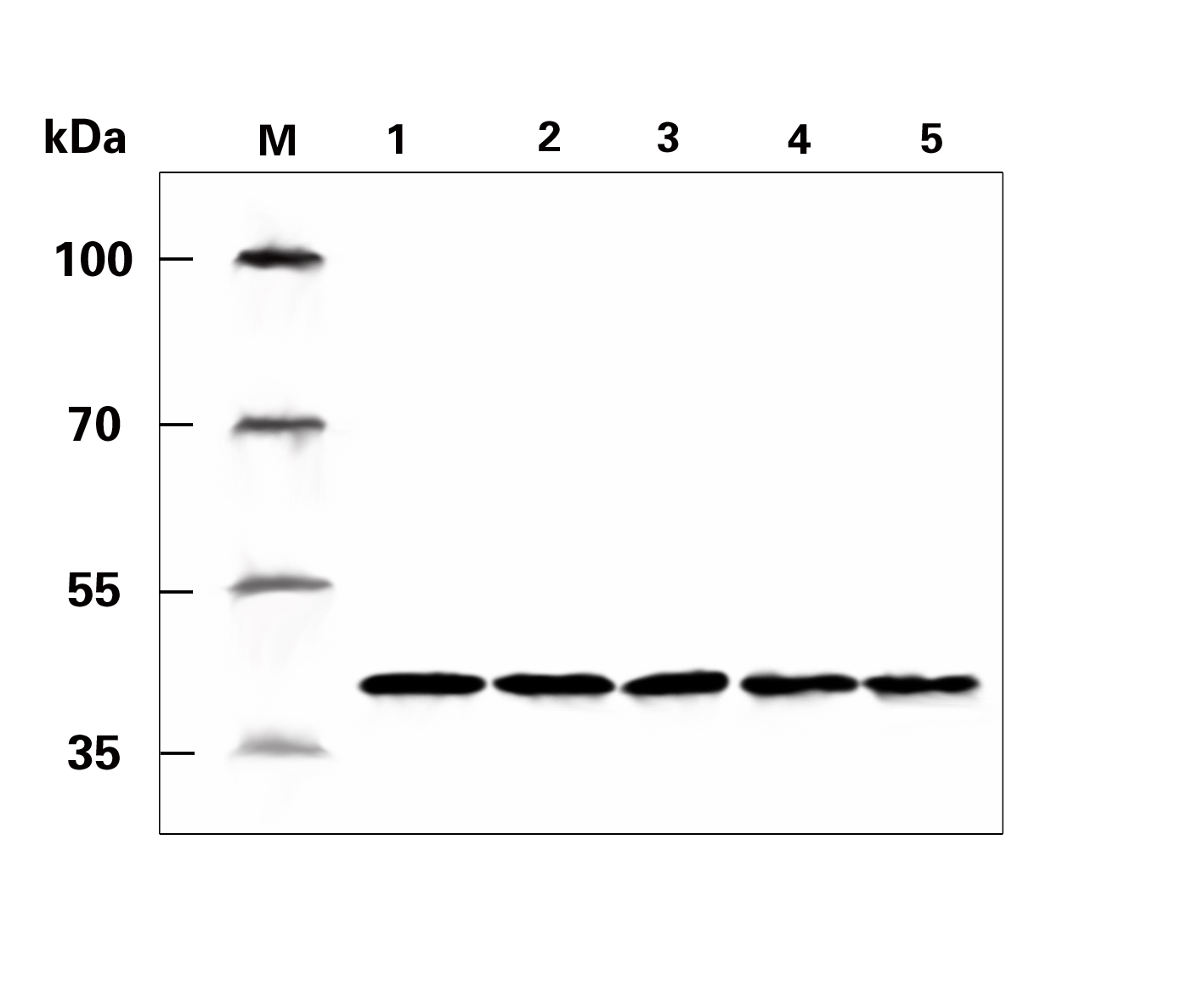


M：marker protein

1：control group

2：Dex group

3: Res group

4: Dex+Res group

5: Res+ P38i group

β-actin=43kDa

Figure 9D1


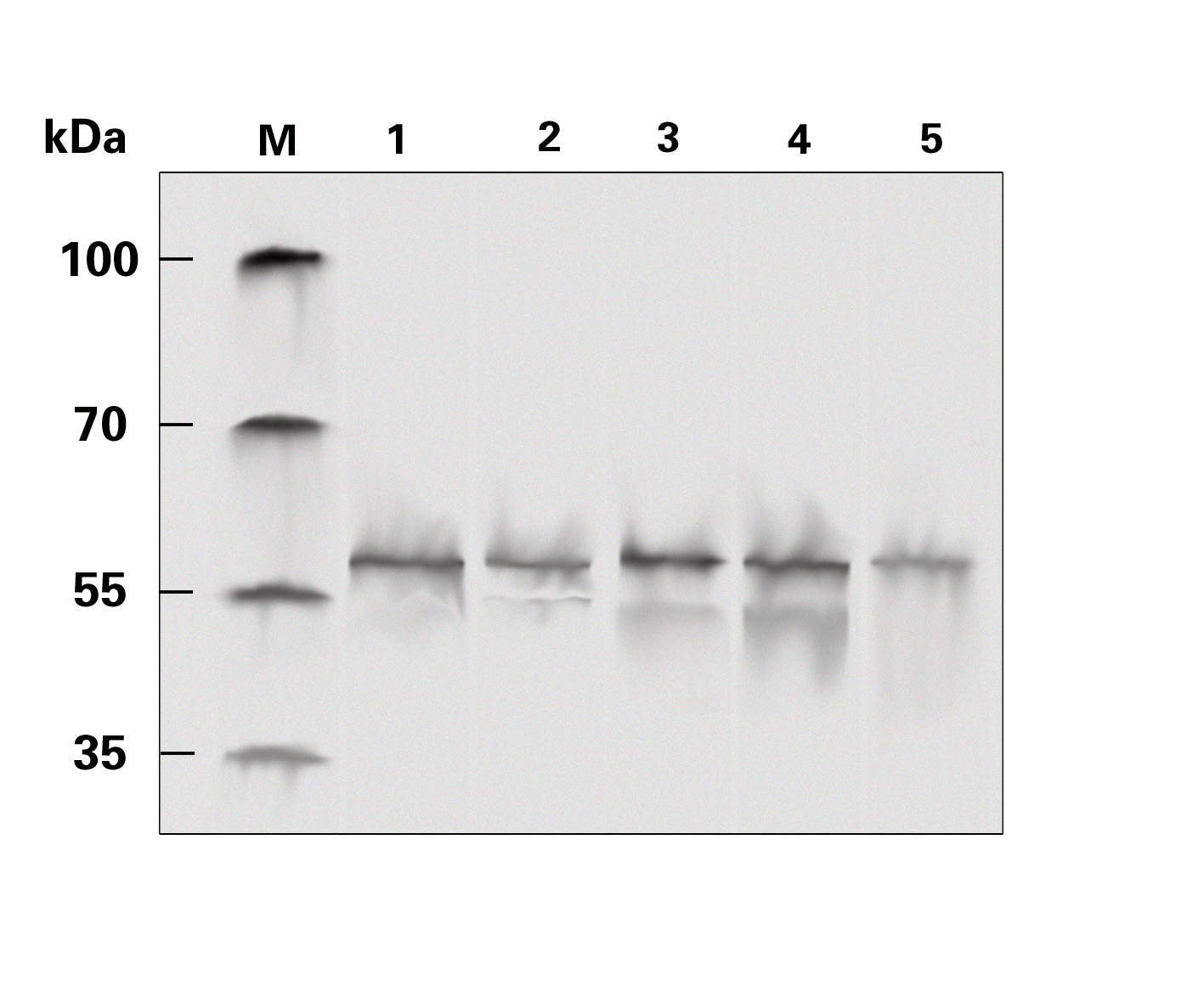


M：marker protein

1：control group

2：Dex group

3: Res group

4: Dex+Res group

5: Res+ JNKi group

*p*-JNK=56kDa

Figure 9D2


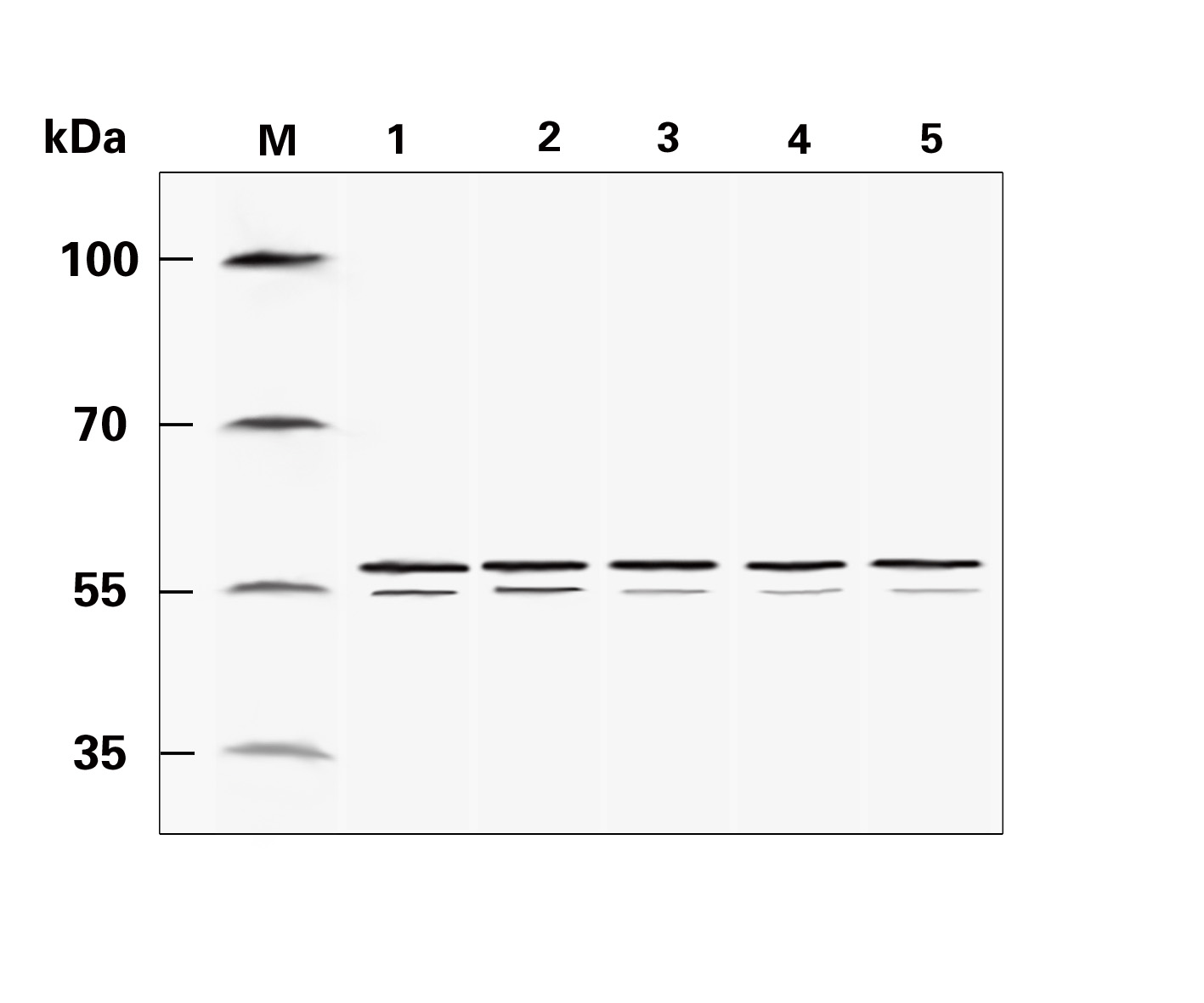


M：marker protein

1：control group

2：Dex group

3: Res group

4: Dex+Res group

5: Res+ JNKi group

*t*-JNK=56kDa

Figure 9D3


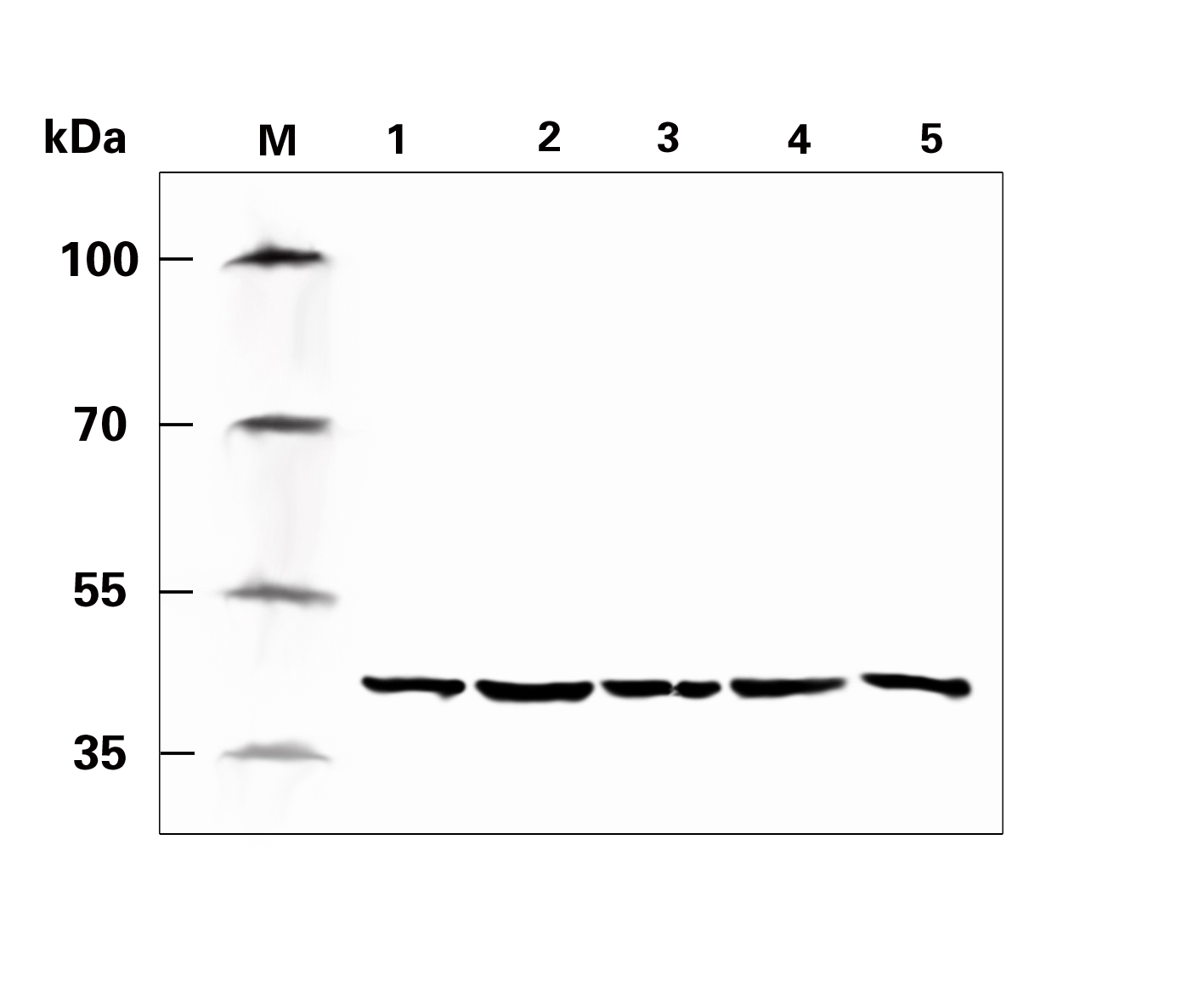


M：marker protein

1：control group

2：Dex group

3: Res group

4: Dex+Res group

5: Res+ JNKi group

β-actin=43kDa
